# NatA engages in multi-factor complexes at the ribosomal polypeptide tunnel exit

**DOI:** 10.1038/s41467-026-68787-5

**Published:** 2026-01-23

**Authors:** Marius Klein, Klemens Wild, Nina McTiernan, Thomas Arnesen, Irmgard Sinning

**Affiliations:** 1 Heidelberg University Biochemistry Center (BZH), Heidelberg, Germany; 2 Department of Biomedicine, University of Bergen, Bergen, Norway; 3 Department of Surgery, Haukeland University Hospital, Bergen, Norway

**Keywords:** Cryoelectron microscopy, Ribosomal proteins, Post-translational modifications

## Abstract

N-terminal acetylation (NTA) is the most common protein modification in eukaryotes, playing a crucial role in proteostasis. Almost 40% of the human proteome is acetylated co-translationally by the NatA complex, which requires prior N-terminal methionine excision (NME). Recently, NatA was shown to form multi-enzyme complexes with MAP1/NAC or MAP2, combining the capabilities of NME and NTA into a single complex. Here, we show that NatA can also form ribosome-independent assemblies with several ribosome associated factors (RAFs). At the ribosome, NatA can form a ternary complex with the abundant pseudoenzyme Ebp1 or a second copy of NatA, which can be coordinated from a different binding site with closer access to a potential substrate. Further, we identify a conserved binding site on NatA, which can be accessed by four RAFs - Ebp1, NAC, Naa10 and HypK, allowing the formation of different multi-factor complexes at the ribosomal tunnel exit. Therefore, our data suggest that NatA constitutes an interaction hub, and contributes to the coordination of co-translational protein maturation.

## Abstract (web summary)

N-terminal acetylation shapes protein fate during protein biosynthesis at the ribosome. Here the authors show that the NatA enzyme forms dynamic multi-factor complexes at the ribosome, acting as an interaction hub that coordinates cotranslational protein maturation.

## Introduction

Most proteins synthesized at the ribosome are co-translationally subjected to enzymatic modification as soon as the N-terminus of the nascent chain starts to protrude from the peptide tunnel exit (PTE)^1^. The N-terminal residue constitutes a crucial signal that affects a protein’s lifetime, localization, and function^2,3^. While translation is universally initiated by methionine in eukaryotes, this residue is often co-translationally removed or modified, with consequences for overall proteostasis and health^2^. The most abundant modifications that are carried out at the PTE are N-terminal methionine excision (NME) by methionine aminopeptidases (MAPs) and N-terminal acetylation (NTA) by N-terminal acetyltransferases (NATs)^1,3–5^. NME occurs on the majority of all cytosolic proteins when the initiator methionine (iMet) is followed by a small and uncharged residue^6^. Unlike bacteria and archaea, eukaryotes express two methionine aminopeptidases with remarkably similar substrate specificities^7,8^. The ancestral MAP1 enzyme is also present in bacteria, while MAP2 likely evolved from MAP1. Throughout this process, MAP2 has acquired several sequence insertions which mediate its interaction with the ribosome^9^. The largest ~60 AS insertion is referred to as the insert domain that enables a central placement and robust interaction at the PTE (Supplementary Fig. 1)^9,10^.

MAP2 and the MAP2-like ErbB3 receptor binding protein (Ebp1) share the same overall fold (Supplementary Fig. 1), but only 25% sequence identity, highlighting their functional distinction^11^. Ebp1 is involved in cancer regulation, translation initiation, apoptosis, as well as cellular growth and differentiation^12^. This rather broad list of reported processes impedes a clear functional classification. MAP2 and Ebp1 employ the same binding site on the ribosome^10,13,14^ and interact with the long rRNA expansion segment 27L (ES27L)^10,13^. The termini of Ebp1 and MAP2 differ strongly, but both confer recruitment of ES27L. While the unstructured MAP2 N-terminus dynamically associates with ES27L, the Ebp1 C-terminus is structured and conformationally fixes the expansion segment^9,10,13^. When and why Ebp1 fixes ES27L and how it is eventually released remains to be elucidated. Ebp1 has been shown to occupy the PTE preferentially during start codon recognition, but can remain associated throughout the entire elongation phase^14^. Similar to the nascent-polypeptide associated complex (NAC, αβ subunits), Ebp1 is expressed equimolar to the ribosome and between 20–60% of all ribosomes are decorated by this MAP2-like protein^14^. The high abundance of Ebp1 suggests that it is not an off-pathway component but a central player in the process of co-translational protein maturation^14^. While Ebp1 is structurally highly similar to MAP2, it is characterized as a pseudo-enzyme^15^, with mutation in the active site which renders it catalytically inactive^11^. Co-translational NME is therefore only carried out by MAP1 and MAP2, which is a prerequisite for several downstream enzymes^5^.

While only two MAPs handle the high demand for NME, the ribosome associated machinery dedicated to NTA is composed of five NATs (NatA-E), which differ in their substrate specificity^16^. Currently available structures of human NatA^17^, yeast NatE^18^ and NatB^19^ reveal distinct differences in ribosome interaction. NatB, NatC and NatE can acetylate the initiator methionine and are therefore in functional competition with MAPs^4,16^. NatA and NatD, however, require prior methionine removal, either by MAP1 or by MAP2. NatA has a broader substrate specificity than NatD and contributes much more prominently to the overall acetylome, with almost 40% of all proteins being potential substrates^20^. The NatA heterodimer consists of the large α-helical subunit Naa15, comprised of TPR motifs^21,22^, and the small Naa10 enzyme with a GNAT^23–25^ (GCN5-related-N-acetyltransferase) fold. In addition to Naa10, NatA can accommodate Naa50, which is another member of the GNAT family^18,26^. Unlike Naa10, Naa50 can acetylate the initiator methionine and does not require prior NME activity^27^.

An unknown fraction of the cellular NatA pool is complexed by the regulatory protein HypK^28^. HypK binds the NatA complex with low nanomolar affinity and inhibits the catalytic activity of the NatA subunit Naa10 (refs. ^21,22,29^), while conformationally constraining and stabilizing the large auxiliary subunit Naa15 (ref. ^17^). Structural data suggest that this stabilizing effect of HypK might prevent unspecific ribosome binding of NatA^17^. Overall, HypK is essential for proper NatA function in vivo, as its deletion entails similar phenotypes as a NatA deletion^28,30,31^.

Previously, we have determined the cryo-EM structure of NatA bound to the human ribosome, and showed that it occupies a distal site that does not interfere with binding of most other ribosome associated factors (RAFs) known to date^17^. Given that NME and subsequent NTA occur in quick succession on a rapidly translating ribosome, the enzymes involved must be tightly coordinated. We previously determined structures of NatA in complex with MAP1 or MAP2, and thereby identified two potential routes that would enable rapid and successive NME and NTA^17^. While MAP2 and NatA could bind independently of other factors, NAC was positioned in between NatA and MAP1 to coordinate their successive enzymatic functions^17,32^. Similar to Ebp1, NAC is highly abundant both on translating and non-translating ribosomes^33^. NAC comprises a central dimerization domain and four charged and mostly unstructured terminal extensions^34^ (Supplementary Fig. 1). Via a short anchoring helix on the NACβ N-terminus, the heterodimer can remain ribosome associated without interfering with most other RAFs^35^. Overall, the NAC extensions have multiple functions involving ribosome binding^35^, chaperoning^36^, nascent chain sensing^37^, as well as the coordination of enzymes^5,17,38,39^ and targeting factors^40–43^. By concurrently recruiting MAP1 via the NACβ C-terminal extension and binding NatA via the NACα C-terminal extension, a trimeric complex of NatA, NAC and MAP1 can assemble around the PTE^17,32^. The C-terminal part of NACα is homologous to HypK and occupies an overlapping binding site on the surface of Naa15 with a central contact helix (cH)^17,32^. The shared binding site enables NACα-cH to displace the HypK-cH and regulate NatA recruitment at the PTE^17,32^. However, not all ribosomes are decorated by NAC and alternative mechanisms of NatA-HypK regulation at the ribosome remain unexplored.

Since various multi-protein complexes of NatA, NAC, MAP1 and MAP2 were found to bind around the PTE, we now explore two possibilities: (1) Can such assemblies also form independent of the ribosome, and (2) can NatA form coordinated assemblies with other RAFs at the ribosome? The previously described distal binding site of NatA would allow complex formation with other RAFs beyond NAC, MAP1 and MAP2 (ref. ^17^). In vitro interaction studies elucidate the interplay between NatA and several interacting proteins, including NAC, HypK and Naa50 independent of the ribosome. Using cryo-EM we find that NatA has a proximal binding site at the PTE that would grant Naa10 immediate access to potential substrates. A conformational change in the Naa10 C-terminal helix establishes a direct contact with NatA at the distal site. In addition, we determined the cryo-EM structure of the ternary NatA-Ebp1-80S complex. In this multi-protein assembly, the Ebp1 C-terminal extension that was previously found to fix ES27L^10,13^ is remodeled, resulting in the displacement of the expansion segment. Surprisingly, the Naa10 and Ebp1 C-terminal helices can also both bind to the previously described HypK/NACα-cH contact site on Naa15 (refs. ^17,32^), putting all of these RAFs into competition at the ribosome.

## Results

### The interplay between NatA binding proteins NAC, HypK and Naa50

**Fig. 1:**
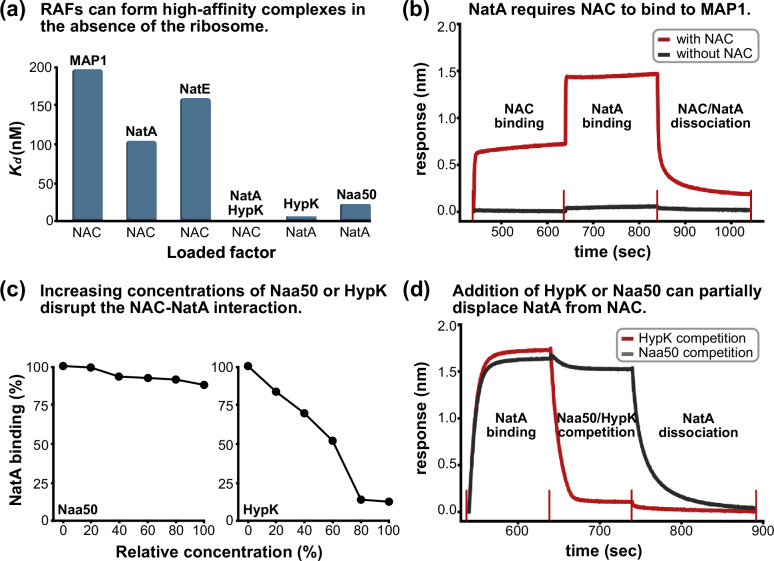
Biolayer interferometry interaction studies of NAC and NatA. **a** Binding affinities determined by BLI. The dissociation constant (*K*_*d*_) is shown as a bar plot for different interactions. The factor that was loaded to the biosensor is labeled below the *x*-axis. HypK binding to NatA appears to abolish NAC binding and a *K*_*d*_ could not be determined. All affinity measurements were done in duplicates on two distinct samples (see Supplementary Figs. 4, 6, 7). **b** BLI ‘order-of-addition’ experiment. NatA strongly associates to MAP1 loaded biosensors (640–840 s) only if NAC is associated first (440–640 s) to mediate the interaction between NatA and MAP1 (red curve). The start- and end-points of subsequent steps are marked. **c** BLI ‘spiking’ experiment. NatA association to NAC loaded biosensors is affected by the presence of Naa50 or HypK. The concentration of Naa50 and HypK is given in % relative to NatA (100% corresponds to an equimolar concentration of NatA and Naa50/HypK). While Naa50 only has a slight negative effect on NatA binding to NAC (left graph), HypK severely diminishes NAC binding to NatA (right graph). Replicates were not done for the ‘spiking experiments’ (*n* = 1). **d** BLI ‘competition’ experiment. NatA was associated to NAC loaded biosensors (540–640 s). The NAC-NatA loaded sensor was then moved into a new well containing either Naa50 (black curve) or HypK (red curve), in addition to NatA. The presence of Naa50 displaced a small amount of NatA from the biosensors, while HypK drastically diminished NatA binding to NAC, consistent with the observations made in (**a**) and (**c**). The start- and end-points of subsequent steps are marked. Source data are provided as a source data file.

N-termini destined for subsequent NME and NTA can be co-translationally processed in quick succession by MAP1 and NatA^17,32^. To study the protein-protein interactions of these enzymes with NAC, we set up in vitro binding experiments in the absence of the ribosome. In addition, we set out to investigate how NatA binding proteins HypK and Naa50 might influence the interaction with NAC. By utilizing size-exclusion chromatography coupled to multi-angle light scattering (SEC-MALS), we observed the formation of a SEC-stable 1:1 complex between NAC and MAP1, as well as NAC and NatA, indicating high-affinity interactions independent of the ribosome (Supplementary Figs. 2 and 3). We then examined the binding kinetics that govern these interactions with biolayer interferometry (BLI). To mimic the immobilization of NAC on the ribosome, we loaded NAC to the sensor tip and titrated either MAP1 or NatA. MAP1 and NAC formed a high affinity complex on the biosensor (*k*_*a*_ = 697,000 M^−1^ s^−1^, *k*_*dis*_ = 0.137 s^−1^, *K*_*d*_ = 196 nM) (Fig. 1a and Supplementary Fig. 4). This interaction has been reported to occur between the MAP1 N-terminal extension and the unstructured NACβ^39^ C-terminus. NatA binding to the NAC-loaded biosensors revealed a ~2-fold slower binding rate but also a ~3.6-fold decrease in the dissociation rate, resulting in an overall higher affinity interaction (*k*_*a*_ = 363,000 M^−1^ s^−1^, *k*_*dis*_ = 0.0379 s^−1^, *K*_*d*_ = 104 nM) (Fig. 1a and Supplementary Fig. 4). To showcase the importance of NAC as a mediator of the MAP1-NatA interaction, we set up an ‘order-of-addition’ experiment. Herein, we immobilized MAP1 on the sensor tip and attempted to associate NatA, resulting only in a weak, and barely detectable interaction (Fig. 1b and Supplementary Fig. 5). In contrast, NatA was able to rapidly bind the MAP1 loaded biosensors if NAC was associated first. NAC can therefore form a trimeric complex with MAP1 and NatA independent of the ribosome.

In human cells, ~20% of Naa50 is associated with NatA to form the NatE complex^44^, while an unknown fraction of NatA or NatE is bound to the regulatory protein HypK^21,28,29^. Using BLI with immobilized NatA, we determined dissociation constants of 0.85 nM and 23.7 nM for MBP-HypK and Naa50 binding, respectively (Fig. 1a and Supplementary Fig. 6), consistent with previous reports^21,22,29,32^. To examine how NatA binding of Naa50 or HypK might affect the recruitment by NAC, we repeated the BLI kinetics experiments between NAC and NatA. While NatA was still able to bind to NAC in the presence of Naa50, the affinity decreased due to a marginally slower on- and faster off-rate (*k*_*a*_ = 322,000 M^−1^ s^−1^, *k*_*dis*_ = 0.0502 s^−1^, *K*_*d*_ = 159 nM) (Fig. 1a and Supplementary Fig. 7), even though the binding sites of Naa50 and NAC do not overlap on the surface of Naa15 (refs. ^17,29,32^). In contrast, the binding sites of HypK and its homolog NACα overlap at the interaction site to Naa15 helices α8, α9 and α10 (refs. ^17,21,22,29,32^). In the presence of HypK, we could no longer measure binding kinetics between NAC and NatA in the absence of the ribosome (Fig. 1a and Supplementary Fig. 7). To validate the slight negative effect of Naa50, and the detrimental effect of HypK on the NAC-NatA interaction, we performed a BLI ‘spiking’ experiment. Herein, increasing concentrations of Naa50 showed a mild decrease of NatA binding to NAC, while HypK much more drastically affected this interaction, consistent with the previous observation (Fig. 1c and Supplementary Fig. 8). Finally, we show that NatA is displaced from NAC loaded biosensors by the addition of HypK, while the addition of Naa50 displaces a small fraction of NatA from NAC (Fig. 1d and Supplementary Fig. 9). Taken together, NAC can form ribosome independent complexes with MAP1, NatA and NatE. Strikingly, in this in vitro setting, NAC does not bind to NatA-HypK, even though the binding sites of NACα and HypK on Naa15 only overlap for the cH^17,32^. This suggests that the ribosome plays a crucial role in mediating the NAC-NatA-HypK interaction.

### NatA forms a ternary complex with Ebp1 and the ribosome

**Fig. 2:**
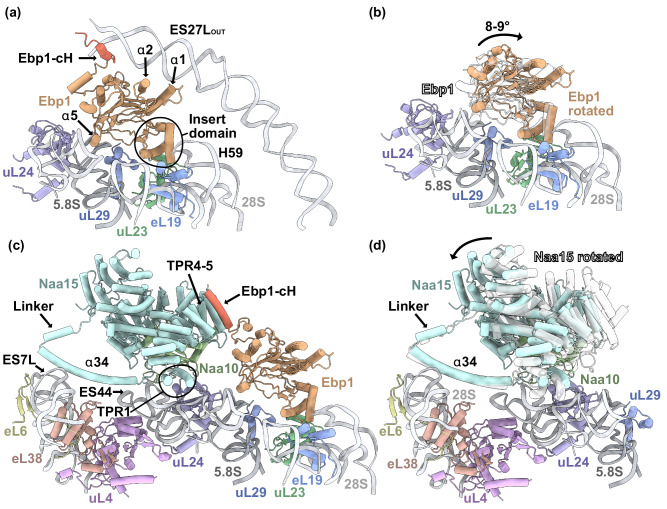
Ebp1 and NatA form a ternary complex with the 80S ribosome. **a** Without NatA, Ebp1 robustly associates with the PTE via interactions with the insert domain (encircled) and helix α5. The long B-arm of ES27L is fixed in the ES27L_OUT_ position through interactions with Ebp1. This protein-RNA interaction is mediated by three contacts, involving the Ebp1-cH (red), as well as helices α1 and α2 (Figure based on PDB: 6SXO^10^). **b** In complex with NatA, Ebp1 is rotated ~8–9° further away from the PTE (orange) compared to an earlier structure in the absence of NatA^10^ (transparent). NatA is not shown in this panel. **c** Upon NatA binding, the Ebp1-cH (red) is remodeled and binds parallel to Naa15 TPR4-5. In complex with NatA, ES27L is released from Ebp1. Binding of NatA at the distal site is mediated by the long α34 anchoring helix and TPR1. **d** In the quaternary NatA-NAC-MAP1-80S complex (PDB: 9FQ0 (ref. ^17^)), NatA is rotated further towards the PTE (transparent). Ebp1 binding to NatA does not result in such a prominent NatA rotation (teal). NAC, MAP1 and Ebp1 are not shown in this panel.

Similar to NAC, Ebp1 is also expressed stoichiometrically with the ribosome and is bound on a large fraction of actively translating and non-translating ribosomes^14,45^. We previously determined the structure of Ebp1 at the human ribosome and found it robustly bound on top of the PTE, where it can fix one of the longest rRNA expansion segments ES27L^10^ (Fig. 2a). Even at high salt conditions (0.5 M KOAc), Ebp1 is still present at the PTE, indicating the robustness of its ribosome interaction (Supplementary Fig. 10).

While the binding site of Ebp1 overlaps with MAP1, MAP2, NAC, NatE, RAC and SRP, the recently identified NatA distal site^17^ might allow coincident binding of NatA. To investigate whether Ebp1 and NatA can form a ternary complex at the ribosome, we mixed these proteins with purified 80S ribosomes and performed single particle cryo-EM.

Indeed, data processing revealed that both Ebp1 and NatA can still bind at their respective sites at the 80S ribosome without steric clash (Fig. 2, Supplementary Figs. 11, 12a, b and Supplementary Table 1). Ebp1 binds in the same way as in our previous structure^10^. However, it is now slightly tilted away from the ribosome by ~8–9° (Fig. 2b), consistent with another reported Ebp1-80S structure^13^. To our surprise, the prominent ES27L interaction is lost when NatA joins the Ebp1-80S complex (Fig. 2a, c). Upon NatA binding, the Ebp1-specific C-terminal extension is recruited by Naa15, and ES27L is released. Strikingly, a helical segment of the Ebp1 C-terminus binds in exactly the same position on Naa15 as observed for the NACα and HypK contact helices (cH)^17,21,22,29,32^ (Fig. 2c). The interaction of this Ebp1-cH with Naa15 will be described and compared with other factors later. Co-Immunoprecipitation Mass Spectrometry (Co-IP-MS) experiments further indicated that endogenous Naa15 interacts not only with Naa10, NACα and HypK, but also with Ebp1 in human cells (Supplementary Fig. 13a). Co-IP-MS was performed without RNAse treatment of the lysates, meaning the Naa15-Ebp1 interaction may be polysome-dependent (Naa15 and Ebp1 bound to separate ribosomes). To determine whether this interaction also occurs polysome-independent, Co-IP was repeated using lysates treated with and without RNAse I or T1 to dissolve polysomes (Supplementary Fig. 13b). Co-IP Western blotting experiments confirmed that Naa15 still interacts with Ebp1 after RNAse treatment, although at reduced levels compared to untreated cells. Interestingly, RNase I digestion resulted in an increased Naa10, but not Naa15, co-precipitating with Ebp1 compared to the untreated control, suggesting that Naa10 may also associate with Ebp1 through Naa16. However, because RNAse T1 treatment led to reduced co-elution of Naa10, this interpretation remains inconclusive (Supplementary Fig. 13b). While these IP experiment confirmed the presence of NatA-Ebp1 complexes in human cells, further in vivo experiments would be required to shed light on the biological significance of this complex.

As recently described, the distal site interaction between NatA and the ribosome is inherently dynamic^17^. While the invariant anchoring helix α34 keeps NatA firmly positioned at the ribosome via interactions with ES7L(A) and ES44A (Fig. 2c), the remaining protein is free to rotate towards the PTE. We showed that this rotating motion is strongly restrained in the presence of MAP2^17^. Further, in a quaternary NatA-NAC-MAP1-80S complex, NAC was shown to form a direct interaction with Naa15 via the NACα C-terminus and pulled the entire NatA complex towards the PTE by 25° (ref. ^17^). In the NatA-Ebp1-80S complex, we now show that while the Ebp1-cH also engages in a direct interaction with NatA in the same position as the NACα-cH, the NatA complex is not pulled towards the PTE but maintains its distance as in the NatA-MAP2-80S complex (Fig. 2d).

Moreover, in the previous structures of NatA at the distal site, the Naa10 C-terminal extension is placed in cis and positioned in parallel to Naa15 TPR6-7 (ref. ^17^). In the reconstruction of the NatA-Ebp1-80S complex, we again observe the Naa10 helix in this position. Further along this trajectory, additional signal for the Naa10 tail is visible and appears to be placed on top of TPR6-7 (Supplementary Fig. 14). However, the ~9 Å local resolution does not allow to pinpoint which residues interact in this area. In hindsight, this signal was also present, but less pronounced, in the reconstruction of the ternary NatA-MAP2-80S and the quaternary NatA-NAC-MAP1-80S complexes^17^, indicating that it is not contributed by Ebp1.

### NatA has a second, ‘proximal’ binding site at the PTE

**Fig. 3:**
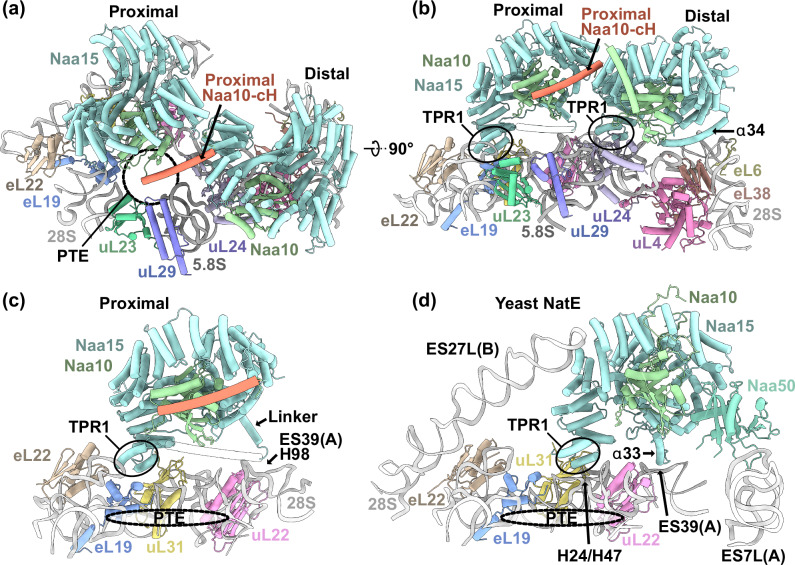
NatA has two binding sites at the PTE. **a** Top view on the PTE (dashed circle) with two copies of NatA positioned at the proximal and distal site. At the proximal site, Naa10 is positioned on top of the PTE while the Naa10-cH (red) contacts the surface of distal Naa15. The distance of proximal and distal Naa10 to the PTE (tunnel exit restriction G2416) is ~35 Å and ~75 Å, respectively. **b** NatA uses the same interaction surface to bind the proximal and distal site, most prominently TPR1 (encircled). **c** Proximal NatA is now positioned in the eL19-eL22-uL31 interface with TPR1 while the flexible linker that connects to the α34 anchor interacts with the exposed kink-loop of ES39(A). Distal NatA is not shown in this figure panel. **d** The position of yeast NatE (PDB: 6HD7 (ref. ^18^)) is shifted relative to the NatA proximal site and binds on top of 28S rRNA helices H24 and H47. The Naa15 anchoring helix α33 (corresponds to α34 in human Naa15) also contacts ES39(A) in this position. Yeast NatE engages in additional rRNA contacts with ES27L(B) and ES7L(A), the latter being mediated by Naa50. In the yeast structure, Naa10 is positioned 50 Å away from tunnel exit restriction G2416 and thereby further away from the PTE than proximal NatA.

The NatA-80S interaction is too dynamic to obtain a well resolved map after data processing when NAC, MAP2 or Ebp1 are not present for stabilization^17^. However, further sub-classification of a previous NatA-80S dataset revealed that the NatA complex has a second binding site in proximity to the PTE (Supplementary Figs. 12c, d and 15 and Supplementary Table 1). At this proximal site, Naa10 is positioned directly above the PTE, resulting only in a short ~35 Å gap to emerging substrates. This immediate substrate access of Naa10 is not granted at the distal site, where a nascent chain would need to close a gap of ~75 Å to reach the active site of Naa10. In vitro acetylation assays confirmed that a nascent chain length of ~68 amino acids allows co-translational acetylation by NatA^32^. Strikingly, the proximal site does not clash with the distal site and allows two copies of NatA to be coordinated at the PTE (Fig. 3a, b). While the distal site of NatA does not interfere with binding of most other RAFs identified so far, the proximal position is located within the highly competed universal adapter site 2 (UAS2)^35^. Even though proximal NatA binds the ribosome in a completely different position, it still uses the same interaction surface for ribosome binding, most prominently Naa15 TPR1. However, TPR1 is now positioned in between eL19, eL22 and uL31 (Fig. 3c). This specific interaction site was previously thought to be exclusively accessed by the NACβ anchoring helix. Only fungal RAC^46^, and the ribosome binding domain of the translocon associated RAMP4 (ref. ^47^) were found to also utilize this site (Supplementary Fig. 16). At the proximal site, the NatA α34 anchor is not visible in our reconstruction, but the trajectory of the upstream linker that connects the anchor to the Naa15 core (Naa15^571-588^) clearly contacts H98 of ES39L (Fig. 3c). Of note, in yeast this ES39L kink-loop also serves as the binding site for the *Sc*NatE anchor^18^, as well as for *Sc*NatB^19^ (Supplementary Fig. 17). Even though proximal *Hs*NatA shares this contact position with *Sc*NatE, the overall binding site differs between the two organisms (Fig. 3c, d). *Sc*Naa15 TPR1 is positioned closer to the PTE on top of H24 and H47 and Naa15 is further stabilized by additional contacts with ES27L. In our map, we clearly see ES27L locked away in the ES27L_in_ position, as previously observed for NatA, NatA-MAP2 and NatA-NAC-MAP1 bound ribosomes^17^.

Analysis of the ternary NatA-NatA-80S complex revealed that distal NatA coordinates proximal NatA via a direct interaction in trans that involves the contact helix of proximal Naa10 (Naa10-cH) (Fig. 3a, b). In our previous NatA-MAP2-80S complex^17^, this Naa10-cH was associated in cis to distal Naa15 and positioned in parallel to Naa15 TPR6-7. Strikingly, the Naa10-cH of proximal NatA is rotated by ~90° (Supplementary Fig. 18) to contact the surface of distal Naa15 at the same site as the HypK-cH^21^, the NACα-cH^17,32^ and the Ebp1-cH described above. Naa10 coordinated at the proximal site is therefore the fourth RAF that has a contact helix that can compete for access to the binding site at Naa15 TPR4-5.

Having identified two possible NatA binding sites, we next asked which site might be used to coordinate Naa50 in the NatE complex. When bound to NatA at the distal site, Naa50 is entirely solvent exposed, positioned far away from potential emerging substrates, and makes no contact to the ribosomal surface, as observed in a recent cryo-EM structure^32^ (Supplementary Fig. 19a). When superimposing the structure of *Hs*NatE with proximal NatA, Naa50 would be positioned directly in the interface between Naa15 and the general RAF adapter protein uL24 next to emerging nascent chains, which would however interfere with distal NatA binding (Supplementary Fig. 19b, c). Whether proximal or distal NatA coordinates Naa50 in vivo remains to be elucidated.

In human cells, at least two distinct NatA complexes exist, composed of either Naa15-Naa10 or Naa16-Naa10 (ref. ^48^). Naa15 and Naa16 are structurally highly similar with 70% sequence identity. Using a Naa15 specific antibody, Co-IP-MS confirmed a co-elution between Naa15 and Naa16, indicating that an interaction between at least two distinct endogenous NatA complexes might occur inside HeLa cells (Supplementary Fig. 13a). To rule out that the Co-IP was polysome-dependent, lysates were treated with and without RNAse I or T1 to disrupt polysomes (Supplementary Fig. 13c). Co-IP Western blotting experiments still clearly show the Naa15-Naa16 interaction after RNAse treatment, supporting the idea that two distinct NatA complexes interact in vivo. As expected, Naa16 co-precipitated at higher levels with Naa10 than with Naa15, indicating that most Naa10-Naa16 complexes are not associated with Naa10-Naa15 (Supplementary Fig. 13c). Whether homodimers of NatA composed exclusively of Naa15 exist in HeLa cells cannot be resolved with this method. While these Co-IP experiments confirmed the presence of NatA-NatA complexes in human cells, further in vivo experiments would be required to shed light on the biological significance of this complex. In order to test whether Naa15 composed NatA can dimerize in vitro and independent of the ribosome, we measured dynamic light scattering (DLS) of increasing NatA concentrations (0.03–8 mg/ml). Indeed, we observe that dimerization also occurs in the absence of the ribosome (Supplementary Fig. 20), at concentrations above ~0.5 mg/ml. We then repeated these experiments with the heterotrimeric NatA-HypK complex and could show that HypK completely blocks the dimerization of NatA.

### Four RAFs compete for access to a conserved binding site on Naa15

**Fig. 4:**
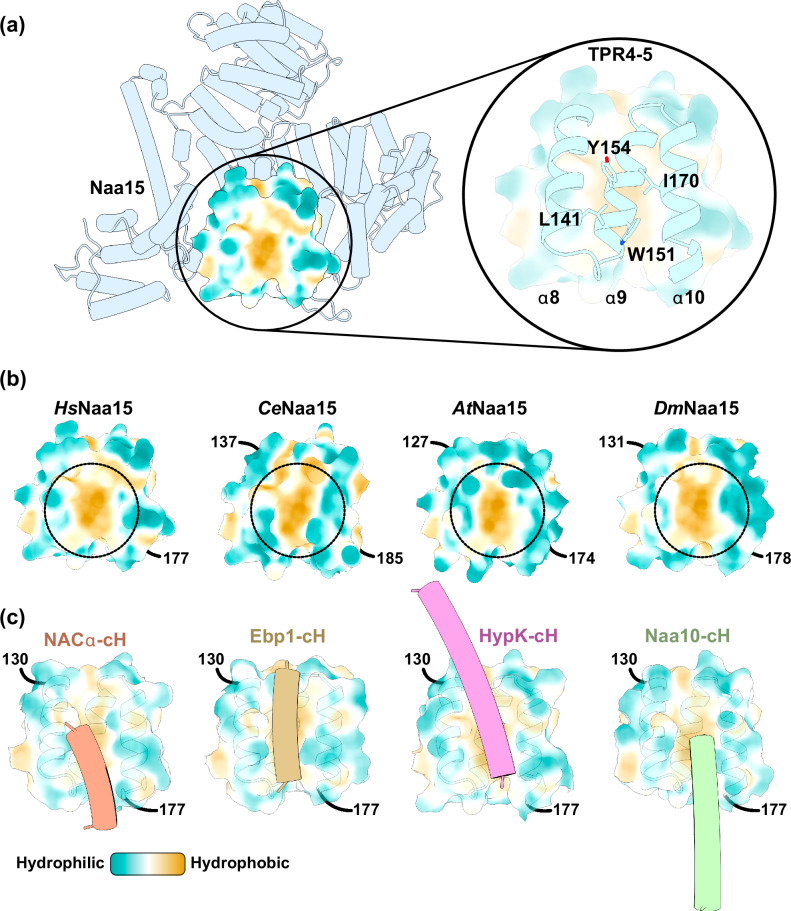
Four RAFs compete for access to a conserved binding site of Naa15 TPR4-5. **a** Cartoon representation of the HsNaa15 Alphafold prediction. The molecular hydrophobicity potential was calculated with ChimeraX and is shown for part of TPR4-5 (Naa15 α8-10). The inset highlights numerous conserved lipophilic residues that contribute to the lipophilic surface potential. **b** AlphaFold predictions of Homo sapiens Naa15 (HsNaa15), Caenorhabditis elegans Naa15 (CeNaa15), Arabidopsis thaliana Naa15 (AtNaa15) and Drosophila melanogaster Naa15 (DmNaa15). The exposed lipophilic patch in between TPR4-5 is conserved between these different organisms. Only part of Naa15 TPR4-5 is shown in the figure with corresponding residue numbers (labeled). **c** The contact helices (cH) of NACα (PDB: 9FQ0 (ref. ^17^)), Ebp1 (this study), HypK (PDB: 6C95 (ref. ^21^)) and Naa10 (this study) all bind at the same position in parallel to Naa15 α8-10.

The broad substrate specificity of NatA, and its ability to occupy two adjacent binding sites on the ribosome independent of a nascent chain or other factors, calls for regulation. HypK has emerged as a major regulator by inhibiting Naa10 catalytic activity^21,22^. In the absence of other RAFs, our attempt to determine the structure of NatA-HypK on the ribosome was not successful (Supplementary Fig. 21), supporting the notion that ribosome binding of NatA-HypK is inhibited by HypK, by restraining Naa15 conformational flexibility^17^. In order to enable ribosome binding, NatA-HypK needs to be rearranged. Recent structures of ribosome-bound NAC and NatA showed that NACα can partially displace HypK^32^ by positioning its cH in parallel to Naa15 TPR4-5 helices α8-10. With our cryo-EM structures, we show that this binding site on Naa15 can also be utilized by Ebp1 and proximal Naa10. The TPR4-5 helices α8-10 therefore constitute a highly competed, general binding site on NatA for the coordination with NAC and MAP1, proximal NatA or Ebp1. The most prominent feature of this site is a conserved hydrophobic surface patch including an aromatic cluster (Fig. 4a, b and Supplementary Fig. 22). Especially a central tryptophan residue is strictly conserved and present from fungi to human. The contact helices of NACα, Ebp1, HypK and Naa10 all share similar features as they are all amphipathic, located close to the C-terminus, and flexibly tethered to the core protein by a linker to allow a positioning of the cH at Naa15 α8-10 (Fig. 4c). Our observation that NACα, Ebp1, HypK and Naa10 occupy the same site on Naa15 consequently puts all of these RAFs into competition at the PTE.

Taken together, human Naa15 is a versatile binding scaffold that can be accessed by Naa10, Naa50, HypK, NAC, Ebp1 and the ribosome. Complex formation with most of these RAFs can occur independent of the ribosome, but the ribosome likely plays a crucial role in mediating the NatA-NAC interaction in the presence of HypK. Multi-factor assemblies that form with distal NatA reveal a conserved hydrophobic patch that is accessed by proximal Naa10, HypK, NAC and Ebp1.

## Discussion

### NAC coordinates co-translational enzymatic activity

We previously showed that NME and NTA can be coordinated between MAP1 and NatA via the abundant NAC^17^. In the absence of the ribosome, we show that NAC can already form a ternary complex with MAP1 and NatA with high affinity. Our BLI data show that NAC is also able to recruit NatA in the presence of Naa50. However, Naa50 negatively influences NAC binding. Such an allosteric binding inhibition has also been described for Naa50 and the NACα homolog HypK^29^. NatE was recently observed in the distal site with Naa50 positioned far away from emerging nascent chain substrates (Supplementary Fig. 18a), but in vivo, human NatE might be coordinated from the proximal position. There, Naa50 would be placed directly on top of the PTE. The position in between Naa15 and uL24 would result in a minimal distance of only 55 Å to emerging nascent chains, which is 30 Å shorter than in yeast (Supplementary Fig. 18) and 60 Å shorter than from distal NatE (Supplementary Fig. 19a, b, d). Of note, Naa50 is inactive in yeast^49,50^ and mainly serves to position NatA at the ribosome, while human Naa50 is catalytically active^29,50^ and would benefit from the closer distance to its acetylation target, the initiator methionine.

In contrast to the slight impact of Naa50 on the NatA-NAC interaction, HypK was able to completely abolish the formation of this complex. Using BLI, we determined a >100x higher affinity of HypK binding to NatA compared to NAC binding to NatA. This drastic difference in *K*_*d*_ partially explains why HypK can outcompete NAC in this in vitro setting. However, the binding sites of NAC and HypK only overlap in the Naa15-cH contact, while both UBA domains bind to distinct sites on Naa15, as evidenced by a recent NatA-HypK-NAC-80S cryo-EM structure^32^. Given that the UBA domains alone were shown to be necessary and sufficient for the high-affinity interaction of HypK^22^ and also NAC^32^ with NatA, we were surprised to find that HypK blocks the NAC-NatA interaction in the absence of the ribosome. Having a closer look on the NatA/HypK-NAC-80S complex, it becomes clear why the NAC-UBA binding site on NatA is disrupted in the NatA-HypK complex. As previously described, binding of NatA to HypK or the ribosome triggers conformational changes in the N-terminal TPRs in opposing directions^17^. Naa15 TPR1-3 is a dynamic and adaptable element within the Naa15 solenoid that rotates around a flexible linker, positioned between helices α6 and α7 (Supplementary Fig. 23). At the ribosome, Naa15 TPR2-3 (helices α4-6) open up to accommodate the NAC-UBA, forming an interaction interface of ~450 Å^2^ (PISA server^51^). In contrast, binding of HypK tightens up TPR1-3, reducing the available interface for Naa15-NAC-UBA to ~180 Å^2^ (PISA server)^51^ (Supplementary Fig. 23).

In conclusion, neither NAC nor the ribosome appear to be independently able to displace HypK. Instead, the ribosome likely weakens the NatA-HypK interaction to allow remodeling of the NAC-UBA binding site on Naa15 TPR2-3. Through this induced-fit-mechanism, NAC is able to contact Naa15 via the high-affinity UBA interaction. With the NAC dimerization domain positioned at the PTE, the NAC-cH is in the perfect position to displace the HypK-cH^32^.

### The NatA proximal site at the PTE

**Fig. 5:**
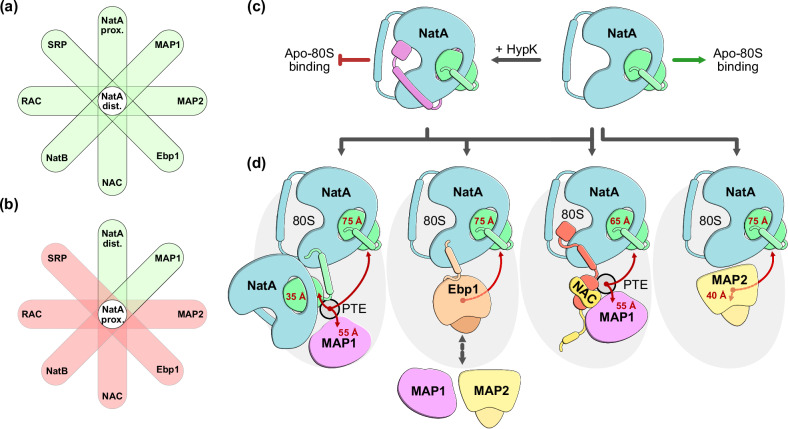
NatA interactions at the human ribosome. **a**, **b** show Venn-like diagrams of different multi-protein assemblies that may compile at the PTE. Sterically possible two-factor combinations are shown in green, while clashing combinations are shown in red. **a** The NatA distal site (white circle) allows concurrent binding of most other RAFs without steric clash (green), including proximal NatA (this study), MAP1 (refs. ^17,39^), MAP2 (ref. ^9^), Ebp1 (ref. ^10^ and this study), NAC^17^, NatB^19^, RAC^46^ and SRP^40^. **b** In contrast, the NatA proximal site (white circle) locates centrally in the highly competed UAS2 (ref. ^35^) and would clash with most other RAFs (red), except for distal NatA and MAP1 (green). **c** HypK binding to the NatA complex seems to inhibit ribosome binding unless HypK is remodeled by another RAF. In the absence of HypK, NatA can bind ribosomes without any other associated factor. **d** Proximal Naa10, Ebp1 and NACα can all compete with the HypK-cH and might facilitate NatA binding at the distal site. To perform successive NME and NTA, proximal NatA could coordinate with MAP1 and distal NatA could coordinate either with NAC-MAP1 or MAP2^17^. Ebp1 does not have MAP activity and might therefore be a placeholder for MAP1 or MAP2. While MAP2 can form a ternary assembly with NatA and the ribosome, there is not structural evidence for a competition with HypK, indicating that NatA-HypK might not independently form a complex with MAP2 at the PTE. Distances from the PTE to the active site of Naa10, MAP1 and MAP2 are labeled in red.

We show that Naa15 can dynamically coordinate Naa10 from two different binding sites. While the proximal site is located centrally within UAS2 (ref. ^35^), the non-intrusive distal site allows NatA to form large multi-protein assemblies at the PTE via the scaffolding function of Naa15 (Fig. 5a, b).

Strikingly, proximal and distal NatA can cooperate to form a ternary structure at the ribosome. The interaction between the two enzyme complexes is mediated by the extended cH of proximal Naa10, which is rotated by ~90° to contact distal Naa15. It is intriguing to speculate whether this structural rearrangement of proximal Naa10 might impact its substrate specificity or enzymatic activity. Similarly, the structural adaptations of Naa15 at the distal site^17^ might have an effect on the function of distal Naa10, which might be further shaped by Naa15 mediated interactions with other RAFs. If and how the catalytic function of NatA might be fine-tuned by its binding site on the ribosome and its RAF interactions remains to be elucidated.

While the GNAT-fold of the Naa10 enzyme is highly conserved between different organisms, its C-terminal extension underwent drastic changes throughout evolution (Supplementary Fig. 24). Particularly in mammals, this extension features the long α-helical cH immediately downstream of the catalytic domain. A frame-shift in the human Naa10 C-terminus has been linked to severe disease^52^, highlighting the importance of this structural feature. Appended to the C-terminal helix, human Naa10 holds a 56-residue long extension of unknown function, enriched in acidic residues (Supplementary Fig. 24). The presence of long unstructured extensions is a common feature of RAFs, also found for instance on the N-termini of Naa30, MAP2, NMT1 and NMT2, the C-termini of Naa15, Naa50 (ref. ^53^) and SRP19, as well as both termini of SRP68, NACα and NACβ.

Most ribosome associated enzymes analyzed so far employ a range of different conformations or even have multiple binding sites on the ribosome (e.g. MAP1 (refs. ^17,19,39^), MAP2 (ref. ^9^), NatA, NatB^19^). In the case of NatA, two ribosomal binding sites can be occupied at the same time, placing two Naa10 enzymes in distinct positions around the PTE. Given the much shorter distance of proximal Naa10 to the PTE, it appears likely that NTA would be carried out by proximal and not distal NatA. The observation that an acetyltransferase can coordinate a second copy of itself at the PTE has also been made for yeast NatB^19^. NatB is structurally highly similar to NatA, with the TPR scaffold protein Naa25 engulfing the enzyme Naa20, but operates independently of NME. It too has a distal and a proximal binding site (Supplementary Fig. 19e, f). Proximal NatB is coordinated by distal NatB, placing proximal Naa20 in closer proximity to a potential emerging substrate. Similar to NatA, the distal position is more off-center and allows concurrent binding of numerous other factors (e.g. MAP1, MAP2, NAC, distal NatA). When a second copy of NatB binds, it becomes positioned in a much more competed area around the inner ring of the PTE. While this would limit the amount of factor assemblies that could compile at the PTE, it results in a shorter distance to an emerging substrate for immediate acetylation of the N-terminal methionine.

Unlike NatB, NatA cannot acetylate methionine and therefore requires prior NME activity by MAP1 or MAP2. We recently showed that distal NatA can coordinate with MAP1 via the scaffolding function of NAC, or form a ternary complex with MAP2 and the ribosome^17^. These structures showcase two possible routes that might enable coordinated successive NME and NTA. Neither route would be accessible to proximal NatA, as it would clash with MAP2 and NAC. However, the dynamic MAP1 interaction with the ribosome would likely not be perturbed by proximal NatA. In such a quaternary NatA-NatA-MAP1-80S assembly, the active sites of MAP1 and proximal Naa10 would be placed directly next to each other, with exceptionally short distances of 55 Å and 35 Å to potential emerging substrates, respectively (Supplementary Fig. 25). Whether MAP1 can bind the ribosome in this position without the coordinating function of NAC remains to be explored.

### NatA regulation at the ribosome

NatA is responsible for the majority of acetylation events in human cells and has the broadest substrate specificity of all NATs, as well as two binding sites on the ribosome. The high activity and versatility of NatA calls for regulation. The function of NatA is regulated by many variables, including Acetyl-CoA^16^, IP6^21^, as well as the presence of the NatE subunit Naa50 (refs. ^29,49,50^) and the regulatory protein HypK^21,22^. Furthermore, there are multiple layers of regulation by HypK, impacting both Naa10 and Naa15. Firstly, HypK inhibits the catalytic function of Naa10 with its N-terminal extension^21,22^. Secondly, HypK interferes with binding of other NatA interactors, such as NAC or Naa50 (ref. ^29^), and prevents the dimerization of NatA. Finally, HypK blocks ribosome binding of NatA, possibly via conformationally restraining the N-terminal TPRs^17^. Recently, NACα was reported to compete with HypK at the ribosome by employing an overlapping binding site in parallel to TPR4-5 (refs. ^17,32^).

Here, we show that there are at least two other RAFs besides HypK and NACα that employ a cH to bind the exposed hydrophobic surface in parallel to Naa15 helices α8-10. Given that NACα has been shown to remodel the HypK-NatA interaction by competing with the cH-binding site^32^, it is tempting to speculate whether Ebp1 and proximal NatA might offer alternate routes for HypK displacement on ribosomes that are not decorated by NAC (Fig. 5c, d). Intriguingly, proximal NatA clashes with NACβ, suggesting that proximal NatA decorated ribosomes do not have NAC. In addition, Ebp1 abundance has been reported to negatively correlate with NAC, also suggesting that most Ebp1 decorated ribosomes might not have NAC^45^. Both of these observations suggest that ternary NatA-Ebp1-80S and NatA-NatA-80S complexes form independent of NAC, thus necessitating alternate mechanisms of NatA regulation. A preliminary attempt to decorate ribosomes with NatA-HypK was unsuccessful, despite a high concentration of RAF relative to the 80S particles (Supplementary Fig. 21). This indicates that NatA-HypK is not able to self-promote ribosome binding, and that at least one copy of NatA without HypK might be required to assemble the ternary NatA-NatA-80S complex.

If Ebp1 and proximal NatA are unable to displace HypK, it would suggest that there is a pool of NatA in the cell that is not complexed by HypK, or that there is another mechanism in place to displace HypK before the Ebp1-NatA-80S and NatA-NatA-80S assemblies can compile at the PTE. The ratio between NatA and NatA-HypK is not well characterized between different organisms, tissues or in the context of disease, and further studies will be required to address this question.

The NatA-Ebp1-80S complex does not hold NME activity, and would therefore be unable to produce NatA substrates. For NatA to perform NTA, Ebp1 would need to be exchanged for one of the two active MAPs (Fig. 5d). Regulating their active counterparts and competing for access to a shared binding site is a common feature of pseudoenzymes, such as Ebp1 (ref. ^15^). Of note, the ribosome biogenesis factor Arx1 in yeast is also a MAP2-like pseudoenzyme that occupies the MAP2 binding site on the late 60S pre-ribosomal particles, until it is later displaced^54,55^. Ebp1 is highly abundant and NatA mediated NTA is among the most widely executed co-translational modifications. Our observation that these two factors interplay at the ribosome, and endogenously interact in HeLa cells, might indicate that Ebp1 is not just a stalling factor employed on hibernating ribosomes or an off-pathway component, but could be a central player in the orchestration of early co-translational processing of nascent chains. Furthermore, we show that the function of Naa15 may go far beyond shuttling Naa10 to the PTE. Instead, Naa15 might constitute a widely accessed interaction hub, employed to coordinate numerous RAFs at the ribosome. Structures of human NatB and NatC at the ribosome are not yet available. As these large enzyme complexes also comprise a solenoidal adapter protein, it is tempting to speculate whether NatB and NatC also serve as a versatile interaction hub, capable of coordinating diverse multi-factor assemblies at the PTE.

## Methods

### Sample preparation

NatA, Naa50, NAC, MAP1, NatA-HypK and MBP-HypK were obtained by insect cell expression, while Ebp1 was produced in *E. coli* cells. For insect cell expression, cells were grown at 27 °C and 80 rpm to be infected at a density of 2 × 10^6^ cells/ml. Expression was continued for 72 h and cells were harvested by centrifugation at 1500 × *g*. For *E. coli* expression, a pre-culture of Rosetta DE3 cells was used to inoculate LB medium to a start optical density (OD_600_) of 0.02. Cells were grown until reaching an OD_600_ of 0.6 and expression was induced with 1 mM IPTG. Expression was continued for 4 h and cells were harvested by centrifugation at 4000 × *g*. After harvesting, cell pellets were washed once with PBS, flash frozen in liquid nitrogen and stored at −80 °C until further use. For purification, pellets were lysed in microfluidizer in the presence of 1 × protease inhibitor (Roche) and cleared by ultracentrifugation at 50,000 × *g* for 20 min. Cleared lysate was passed through 0.2 µm syringe filter and proteins were purified at 4 °C.

All Ni^2+^-IMAC purifications were done in IMAC-A (20 mM HEPES, 500 mM NaCl, 20 mM Imidazole, pH 7.4). All Strep-II-affinity chromatography steps were done in Strep buffer (20 mM HEPES, 500 mM NaCl, pH 7.4). All size exclusion chromatography (SEC) runs were done in SEC buffer (20 mM HEPES KOH, 150 mM KOAc, 5 mM MgOAc_2_, 1 mM TCEP, pH 7.4). All purification procedure followed a similar scheme, with one or two subsequent affinity-chromatography capture steps, an optional on-column digest, and a final SEC to obtain pure protein and stoichiometric complexes in SEC buffer.

NatA, Naa50, MAP1 and Ebp1 were captured on Ni^2+^-Agarose beads (Qiagen) via a His-tag on Naa10, Naa50, Ebp1 or MAP1. After washing, the tag was removed by on-column digestion with 3C protease (for NatA, MAP1 and Naa50) or TEV protease (for Ebp1). For NAC, the same workflow was done (as recently described^17^) with Streptavidin Beads (Purecube) to capture the Strep tag-II on the NACα N-terminus, followed by on-column 3C digestion. His-MBP-HypK was purified on Ni^2+^- Agarose beads (Qiagen) and eluted with imidazole. Stoichiometric NatA-HypK was obtained by co-expressing His-MBP-TEV-HypK and Naa15 with Naa10-3C-Strep-II. Purification was done with two capture steps with Ni^2+^-IMAC and on-column TEV digestion, followed by Strep-affinity chromatography with on-column 3C digestion. To obtain purified MBP as a negative control in BLI measurements, His-MBP-TEV-HypK was captured on Ni^2+^-Agarose beads (Qiagen). HypK was eluted by TEV digestion over-night and remaining His-MBP was eluted with 20 mM Imidazole.

After the capture steps, proteins were concentrated to ~10 mg/ml and subjected to SEC. NatA, NatA-HypK, MBP-HypK, MBP, MAP1 and Ebp1 were run on an S200 16/600 column (Cytiva), while NAC and Naa50 were run on an S200 Increase 10/300 column (Cytiva). The protein purity was evaluated by SDS-PAGE and coomassie staining (Supplementary Fig. 26).

Human 80S ribosomes were isolated from HeLa S3 cells as described^10^, with the sucrose cushion adjusted to a KOAc concentration of 500 mM. Following the purification procedure, ribosomes were subjected to SEC (20 mM HEPES KOH, 600 mM KOAc, 5 mM MgOAc_2_, 1 mM TCEP, pH 7.4) on a Superose 6 10/300 column (Cytiva) to remove any co-purified RAFs from the PTE. Afterwards, the buffer was adjusted to 20 mM HEPES KOH, 150 mM KOAc, 5 mM MgOAc_2_, 1 mM TCEP, pH 7.4. After purification, ribosomes were flash frozen in liquid nitrogen and stored at −80 °C until further use.

### Cryo-EM grid preparation

Before sample freezing, copper grids were glow-discharged in the Solarus plasma cleaner (Gatan, Inc.) for 1 min in oxygen atmosphere. For the data acquisition of NatA, 500 nM of 80S ribosomes were mixed with 12.67 µM of purified NatA, as recently described^17^. For the complex of Ebp1 and NatA, 500 nM of 80S ribosomes were incubated with 15 µM of Ebp1 and 12.57 µM of NatA for 30 min at room temperature. For the attempted complex formation of NatA-HypK with the 80S ribosome, 20 µM NatA-HypK were incubated with 800 nM 80S ribosomes for 30 min at room temperature. From each sample, 3 µl were vitrified in liquid ethane using the Vitrobot Mark IV (FEI company) on R2/1 300 mesh copper grids (Quantifoil). Freezing was done at 4 °C with Whatman #1 filter papers. A blot force of 0 was used with 10 s wait time and 100% humidity. Until data acquisition, grids were stored in liquid nitrogen.

### Data collection

The NatA-80S datasets were acquired as preciously described^17^. Briefly, two datasets of the NatA-80S sample were collected on a Glacios transmission electron microscope (Thermo Fisher Scientific) operated at 200 keV with the Falcon 3 direct electron detector (Thermo Fisher Scientific) that collected at a pixel size of 1.223 Å/pixel and a magnification of 120,000. The total dose per micrograph was 51.65 and 55.07 e^-^/Å^2^ for the two datasets, respectively. The NatA-Ebp1-80S dataset was also collected on the Glacios transmission electron microscope at a magnification of 120,000 and a pixel size of 1.223 Å/pixel. The total dose for this dataset was 27.51 e^-^/Å^2^. The data acquisitions were set up and monitored with EPU (Thermo Fisher Scientific).

### Data processing

Detailed descriptions of the processing schemes for all datasets can be found in the Supplementary Information (Supplementary Figs. 11, 15, 21). Briefly, movies were imported and pre-processed in CryoSPARC^53^, including Patch Motion Correction and Patch CTF Estimation. Particles were picked with the Blob Picker and extracted followed by 3 subsequent rounds of 2D classification. Ab-initio Reconstruction into three classes was done with the remaining particles and used to seed a Heterogeneous Refinement. 80S ribosome containing class was subjected to Homogenous Refinement. Two rounds of focused 3D Classification with different masks around the PTE were performed to deal with the local heterogeneity. After classification, a Particle Subtraction job was done with a negative mask generated in ChimeraX^56^, followed by a final Local Refinements. Local Resolution Estimation jobs were run for the final refinements.

### Cryo-EM model building, refinement, and analysis

The high-resolution cryo-EM structure of the human ribosome (PDB ID: 6QZP)^57^ was used as a starting point for model building. For building of the ternary NatA-Ebp1-80S complex, our NatA-MAP2-80S structure (PDB-ID: 9FPZ)^17^ and Ebp1-80S structure (PDB-ID: 6SXO^10^) were used as a starting model. For building of the NatA-NatA-80S structure, the cryo-EM structure of NatA-MAP2-80S was also used (PDB-ID: 9FPZ^17^). For model building, the component proteins were first rigid body fitted into the cryo-EM map using ChimeraX^56^. Atomic model building was performed in Coot^58^ and the preliminary model was refined and validated in PHENIX suite^59,60^. The refinement statistics for all cryo-EM datasets are shown in Supplementary Table 1.

### Biolayer interferometry

All BLI experiments were performed in BLI buffer (20 mM HEPES KOH, 150 mM KOAc, 5 mM MgOAc_2_, 1 mM TCEP, pH 7.4 with the addition of 0.01 % Tween-20). For kinetic measurements, biotinylated NAC or NatA was immobilized on streptavidin (SA) biosensors (Fortébio). Biotinylation was carried out using the EZ-Link NHS-PEG4-Biotin (Thermo Fisher Scientific) and biotinylation efficiency was quantified using the Pierce Biotin Quantification kit (Thermo Fisher Scientific). Proteins were deliberately under-labeled to a biotin labeling efficiency of <5% (<5 biotin molecules for 100 proteins) to control that proteins loaded to the tip do not contain more than one label, as to not interfere with analyte binding. After a 60 s sensor scan, the ligand (NAC or NatA) was loaded to the tip for 180 s. After two washing steps for 200 s, the analyte (NatA, MAP1, Naa50, MBP-HypK, NatA-Naa50 (1:1) or NatA-HypK) was associated for 100 s, followed by dissociation for 150 s. A control sensor was used in parallel, to ensure that analytes do not unspecifically interact with the sensor tip. Analytes were loaded to the tip in 1:1 dilutions ranging from 250–15.6 nM for MAP1, 250–7.8 nM for NatA, 500–31.3 nM for MBP-HypK, 125–7.8 nM for Naa50, 125–7.8 nM for NatA-Naa50 (1:1) and 250–62.5 nM for NatA-HypK. All measurements were done in duplicates at 25 °C and data was analyzed using the HT 10.0 software (Fortébio) with a global 1:1 fit model.

Spiking experiments were set-up the same way as the kinetic experiments. After loading and washing biotinylated NAC, NatA was associated for 100 s (250 nM) in the presence of increasing concentrations of Naa50 or MBP-HypK (0–100% of the NatA concentration). Finally, all six biosensors were moved to BLI buffer for 150 s for dissociation of the NatA. Control experiments were done in parallel to ensure that Naa50 does not bind to NAC, MBP does not interfere with the NAC-NatA interaction, MBP-HypK does not bind to NAC, and that none of the used analytes (Naa50, MBP-HypK, MBP and NatA) unspecifically interact with unloaded SA-biosensors.

An ‘order-of-addition’ experiment was done to showcase the role of NAC as a mediator between NatA and MAP1. Herein, a 60 s sensor scan was performed, before loading biotinylated MAP1 for 180 s, followed by two 100 s washing steps in BLI buffer. Two biosensors were then loaded with 2 µM NAC for 200 s and subsequently moved into a new well for another 200 s, which also contained 2 µM NAC. For one of the biosensors, this well also contained 2 µM NatA in addition to the 2 µM NAC. The first biosensor was therefore used as a negative control to show that movement between two wells containing 2 µM NAC does not result in a second association curve. The second biosensor was used to show that NatA rapidly associates to MAP1 loaded biosensors if NAC was loaded first to mediate the interaction. In addition to this main experiment, two control sensors were used to show that NatA does not rapidly associate to MAP1 loaded sensors without the mediating function of NAC. The second control was done to show that NAC and NatA do not bind unspecifically to unloaded SA-biosensors.

Finally, a BLI competition experiment was set up to validate the negative effect of Naa50 and MBP-HypK on the NAC-NatA interaction, observed in the kinetic and spiking experiments. The set-up was similar to the kinetic experiments. After a 60 s sensor scan, NAC was loaded to the sensor tip for 180 s, followed by two 100 s washing steps. Then, 250 nM NatA was associated to three NAC-loaded biosensors for 100 s. Subsequently, all three sensors were moved into a new well also containing 250 nM NatA with the addition of either 250 nM MBP (negative control), 250 nM MBP-HypK or 250 nM Naa50. This ‘displacement’ step was carried out for 100 s, followed by a 150 s dissociation step in BLI buffer. In parallel to the main measurements, five control sensors were used to ensure that Naa50 and NatA-HypK do not bind to NAC, and that none of the used analytes (NatA, MBP-HypK or Naa50) unspecifically bind to unloaded SA-biosensors.

### SEC-MALS

SEC-MALS of *Hs*MAP1 and *Hs*NAC complexes was performed with an S200 Increase 3.2/300 column (Cytiva) on an Äkta Pure (Cytiva) connected to a DAWN® Heleos II 8 + MALS detector and the Optilab® T-rEX dRI monitor (Wyatt Technology). SEC was performed at room temperature in 20 mM HEPES KOH pH 7.4, 150 mM KOAc, 5 mM MgOAc_2_, 1 mM TCEP. The protein concentration of NAC and MAP1 was 4 mg/ml in each SEC-MALS run and complexes were assembled by incubation for 20 min at room temperature prior to injection. Data analysis was done in Astra 6 (Wyatt Technology) assuming a dn/dc value of 0.185 ml/g.

SEC-MALS of *Hs*NatA complexes was performed at EMBL Heidelberg with an S200 Increase 10/300 GL column (Cytiva) on an Agilent 1260 Infinity II HPLC system (Agilent). Chromatography was run at 0.5 ml/min at room temperature in 20 mM HEPES KOH, 150 mM KOAc, 5 mM MgOAc_2_, 1 mM TCEP, pH 7.4. NatA and NAC were injected at a concentration of 2 mg/ml and 4 mg/ml, respectively. The system outlet was connected to MiniDAWN and Optilab MALS system (Wyatt Technology). Data analysis was done in Astra 8 (Wyatt Technology) assuming a dn/dc value of 0.185 ml/g.

### Dynamic light scattering (DLS)

DLS measurements were done to assess the concentration dependent dimerization of *Hs*NatA. A NatA and NatA-HypK dilution series was made from 8 mg/ml to 0.03125 mg/ml in 20 mM HEPES KOH, 150 mM KOAc, 5 mM MgOAc_2_, 1 mM TCEP, pH 7.4. Data was collected at 20 °C on the Prometheus Panta (NanoTemper) using Prometheus High Sensitivity Capillaries (NanoTemper). Data analysis was done on the Prometheus Panta Analysis Software (NanoTemper).

### Ebp1-80S salt sensitivity testing by Western-blotting and immunodetection

To determine the minimal salt concentration required to displace Ebp1 from human ribosomes, a series of SEC runs were performed, followed by SDS-PAGE, Western blotting and immunodetection. SEC runs were performed in SEC buffer (20 mM HEPES KOH, 350–700 mM KOAc, 5 mM MgOAc_2_, 1 mM TCEP, pH 7.4) with varying KOAc concentrations. The experiment was repeated four times with different salt concentrations (350 mM KOAc, 500 mM KOAc, 600 mM KOAc, 700 mM KOAc). For each experiment, 50 µl of HELA ribosomes (final concentration 125 nM) were mixed with 130 µl of dilution buffer (20 mM HEPES KOH, 350 mM KOAc, 5 mM MgOAc_2_, 1 mM TCEP, pH 7.4) and 20 µl of His-Ebp1 (final concentration 60 µM). For each of the four measurements, 40 µl were injected onto the SEC column. Before injection, the sample was centrifuged for 20,000 × *g* for 5 min. SEC was performed at room temperature on as Superose 6 5/150 GL column (Cytiva) and absorption was tracked at 254 and 280 nm. The prominent high-molecular weight peak with strong absorption at 254 nm corresponded to the ribosomes which were separated from unbound Ebp1. Peak ribosome fraction was applied to SDS-gel and blotted onto a PVDF membrane. Finally, anti-HIS immunodetection was done with a primary antibody against the His-tag of Ebp1 and an alkaline phosphatase conjugated secondary antibody. As a negative control, SEC was performed with ribosomes without the addition of His-Ebp1. As a positive control, pure His-Ebp1 (3 µg) were loaded on the SDS-Gel.

### Immunoprecipitation and on-bead digestion

HeLa cells at 80% confluency in 10 cm culture dishes were harvested by scraping and pelleted by centrifugation at 17,000 × *g* for 15 s at 4 °C. The cell pellets were lysed by resuspension in 500 µl IPH lysis buffer (50 mM Tris-HCl pH 8.0, 150 mM NaCl, 5 mM EDTA, 0.5% NP-40, 1x protease inhibitor cocktail) and incubated on a rotator for 15 min at 4 °C. The cell extracts were then centrifuged at 17,000 × *g* for 5 min at 4 °C. The supernatants were collected, and the total protein concentrations were measured using Pierce BCA assay (Thermo Fisher Scientific). Cell lysates containing 1 mg of total protein were mixed with 4 µg of rabbit polyclonal anti-Naa10 (BioGenes), 4 µg rabbit polyclonal anti-Naa15 (BioGenes) or 4 µg rabbit IgG isotype control (Thermo Fisher Scientific) and incubated on a rotator for 3 h at 4 °C. Subsequently, 30 µl of equilibrated Dynabeads Protein G (Invitrogen) was added to each sample and incubated on a rotator overnight at 4 °C. The next day, the magnetic beads were washed three times in IPH lysis buffer and two times in wash buffer without detergent (50 mM Tris-HCl pH 8.0, 150 mM NaCl, 5 mM EDTA, 1x protease inhibitor cocktail).

After removal of the final wash, the magnetic beads were resuspended in 40 µl of Trypsin buffer (50 mM Tris pH 8.0, 1 mM CaCl_2_). Proteins were denatured and reduced by adding 10 mM dithiothreitol (DTT) and heating the samples at 95 °C for 5 min. Samples were then alkylated by incubation with 20 mM chloroacetamide (CAA) for 1 h on a shaker at room temperature. Afterwards, the CAA reaction was quenched by adding 2 mM DTT. Samples were digested using Trypsin in a 1:50 ratio (µg protease:µg protein) and incubated overnight on a shaker at 37 °C. Following digestion, the peptide solutions were separated from the beads, transferred to new tubes and the digestion reaction was quenched by adding trifluoroacetic acid (TFA) to a final concentration of 1%. Samples were desalted using Oasis HLB 96-well µElution Plate (Waters) according to the instruction manual.

### LC-MS/MS analysis

For the LC-MS/MS analysis, a total of 12 samples were analyzed. These included four independent biological replicates for each condition: NAA10-IP, NAA15-IP, and control rabbit IgG-IP. 0.5 µg tryptic peptides in 2% acetonitrile (ACN) and 0.5% formic acid (FA) were injected into an Ultimate 3000 RSLC system (Thermo Scientific) connected online to an Exploris 480 mass spectrometer (Thermo Scientific) equipped with EASY-spray nano-electrospray ion source. Peptides were trapped on a pre-column (Acclaim PepMap 100, 2 cm × 75 µm ID nanoViper column, packed with 3 µm C18 beads), and then separated on an analytical column (PepMap RSLC, 25 cm × 75 µm ID EASY-spray column, packed with 2 µm C18 beads) using a biphasic ACN gradient from two nanoflow UPLC pumps at a flow rate of 250 nl/min. Solvent A was 0.1% FA (v/v) in water and solvent B was 100% ACN. The gradient used was: 5% B for 5 min, 5–8% B for 1 min, 8–28% B for 42 min, 28–40% B for 15 min, and 40–85% B for 2 min. Very hydrophobic peptides were eluted by 85% B for 8 min.

Peptides were detected in the Exploris 480 Mass Spectrometer with FAIMS (high-field asymmetric waveform ion mobility spectrometry) enabled using two compensation voltages (CVs): −45 V and −65 V. The mass spectrometer was operated in data-dependent-acquisition (DDA) mode, switching between full scan MS and MS/MS acquisition. The cycle time was 1.5 s/CV and MS spectra were acquired in the range 375–1500 m/z with resolution 120,000 at *m*/*z* 200, automatic gain control (AGC) target of 3e6 and maximum injection time (IT) at auto. The most intense peptides with charge states 2 to 5 were sequentially isolated with an AGC target of 1e5, maximum IT of 75 ms, and isolation width of 1.6 m/z followed by HCD (higher-energy collision dissociation) fragmentation with a normalized collision energy of 30%. Fragments were detected at a resolution of 15,000 at *m*/*z* 200, with first mass fixed at *m*/*z* 120. Dynamic exclusion was set to 30 s and “exclude isotopes” was enabled. The resulting raw files were processed using Proteome Discoverer (version 2.5) and the protein sequence database of Swiss-Prot annotated human protein sequences (20,435 sequences, retrieved April 2024). The search parameters included trypsin as protease with maximum two missed cleavages allowed and minimum peptide length was six. Cysteine carbamidomethylation was set as static modification and variable modifications were methionine oxidation and N-terminal acetylation. The target false discovery rate (FDR) was set to 0.01 (strict) and 0.05 (relaxed). Precursor and fragment mass tolerances were 10 ppm and 0.02 Da, respectively. Further analysis of the data was carried out using Perseus software (version 2.0.1.1). Data was filtered to remove contaminants and proteins with low/medium FDR or without four valid values in at least one group. Data were log2-transformed and imputed based on normal distribution. Significance was determined using a two-sample two-tailed Student’s *t* test in Perseus, and the cut-off threshold was *p* value < 0.01 and log2 fold change >2.

### Co-immunoprecipitation and Western blot analysis

A total of 2 × 10^6^ HeLa cells were seeded in 10 cm culture dishes. After 24 h, cells were transiently transfected using XtremeGENE 9 (Roche) according to the manufacturer’s protocol. For V5-IP, cells were transfected with 7 µg of plasmid encoding NAA15-V5, NAA10-V5 or an empty vector as a control. For FLAG-IP, cells were co-transfected with 4 µg of plasmid encoding FLAG-EBP1, NAA15-V5, and NAA10-V5. As a control, cells were co-transfected with NAA15-V5, NAA10-V5 and either empty vector or LacZ-V5. Forty-eight hours after transfection, cells were washed with 1x PBS and harvested by scraping. Cells were collected by centrifugation at 17,000 × *g* for 15 s at 4 °C. Cell pellets were lysed in 250 µl of lysis buffer (10 mM Tris-HCl pH 7.5, 150 mM NaCl, 0.5 mM EDTA, 0.5% NP-40, 1x protease inhibitor cocktail) and incubated on a rotator for 15 min at 4 °C. Lysates were then cleared by centrifugation at 17,000 × *g* for 5 min at 4 °C, and the total protein concentrations were determined using the Pierce BCA Protein Assay Kit (Thermo Fisher Scientific). The lysates were adjusted to equal protein concentrations and then diluted 1:2 in dilution buffer (10 mM Tris-HCl pH 7.5, 150 mM NaCl, 0.5 mM EDTA, 1x protease inhibitor cocktail). To dissolve polysomes, lysates were treated with RNase I (18 U/mg protein) (Thermo Fisher Scientific) or RNase T1 (1000 U/ml lysate) (Thermo Fisher Scientific) for 30 min at room temperature on a rotator. RNase activity was quenched with SUPERaseIn RNase Inhibitor (Invitrogen). For Western blot analysis, 20 µl of each lysate was mixed with SDS-sample buffer to a final concentration of 1x and heated at 95 °C for 5 min. Immunoprecipitation was performed using FLAG- or V5-trap agarose beads (Proteintech) following the manufacturer’s protocol. Each lysate was incubated with 25 µl of pre-equilibrated beads overnight at 4 °C on a rotator. The next day, beads were washed three times with 600 µl of wash buffer (10 mM Tris-HCl pH 7.5, 150 mM NaCl, 0.05% NP-40, 0.5 mM EDTA). After the final wash, beads were resuspended in 30 µl 2x SDS-sample buffer and heated at 95 °C for 5 min to elute immunocomplexes. Lysate and IP samples were separated by SDS-PAGE and transferred to nitrocellulose membranes using the Trans-Blot Turbo system (Bio-Rad). Membranes were blocked in 5% non-fat dry milk and incubated overnight at 4 °C with primary antibodies: anti-V5 (1:2000, Invitrogen, R960CUS), anti-FLAG (1:500, Sigma-Aldrich, F1804), and anti-NAA16 (1:1000, Sigma-Aldrich, HPA040157). The following day, membranes were washed three times with 1xPBS-T (0.1% Tween-20) and incubated for 1 h at room temperature with HRP-conjugated secondary antibodies: anti-mouse (1:3000, Cytiva, NA931) or anti-rabbit (1:3000 dilution, Cytiva, NA934). After three washes with 1xPBS-T, blots were imaged using the ChemiDoc XRS+ system (Bio-Rad).

### Figure preparation

Figures were prepared in UCSF ChimeraX^56^ and volcano plots were created using VolcaNoseR^61^.

### Reporting summary

Further information on research design is available in the Nature Portfolio Reporting Summary linked to this article.

## Supplementary information

Supplementary Information

Reporting Summary

Transparent Peer Review file

## Source data

Source data

## Data Availability

All structural data that support the findings of this study have been deposited in the Protein Data Bank (PDB) (coordinates) and EMDB (maps) with the accession codes 9GJ5 and EMD-51382 for the ternary NatA-Ebp1-80S assembly and 9GJ6 and EMD-51383 for the ternary NatA-NatA-80S assembly. Of note: only the PTE is modeled for the human 80S ribosome. The entire human 80S ribosome can be superposed on PTE components (except 28S rRNA helix H59, which is flexible). The mass spectrometry proteomics data have been deposited to the ProteomeXchange Consortium via the PRIDE^62^ partner repository with the dataset identifier PXD059724. Source data are provided with this paper.

## References

[1] Kramer, G., Shiber, A. & Bukau, B. Mechanisms of cotranslational maturation of newly synthesized proteins. *Annu. Rev. Biochem.* **88**, 337–364 (2019). 30508494 10.1146/annurev-biochem-013118-111717

[2] Varland, S., Osberg, C. & Arnesen, T. N-terminal modifications of cellular proteins: the enzymes involved, their substrate specificities and biological effects. *Proteomics* **15**, 2385–2401 (2015). 25914051 10.1002/pmic.201400619 PMC4692089

[3] Aksnes, H., Ree, R. & Arnesen, T. Co-translational, post-translational, and non-catalytic roles of N-terminal acetyltransferases. *Mol. Cell* **73**, 1097–1114 (2019). 30878283 10.1016/j.molcel.2019.02.007 PMC6962057

[4] Deng, S. & Marmorstein, R. Protein N-terminal acetylation: structural basis, mechanism, versatility, and regulation. *Trends Biochem. Sci.* **46**, 15–27 (2021). 32912665 10.1016/j.tibs.2020.08.005 PMC7749037

[5] Gamerdinger, M. & Deuerling, E. Cotranslational sorting and processing of newly synthesized proteins in eukaryotes. *Trends Biochem. Sci.* **49**, 105–118 (2024). 37919225 10.1016/j.tibs.2023.10.003

[6] Sherman, F., Stewart, J. W. & Tsunasawa, S. Methionine or not methionine at the beginning of a protein. *Bioessays* **3**, 27–31 (1985). 3024631 10.1002/bies.950030108

[7] Addlagatta, A., Hu, X., Liu, J. O. & Matthews, B. W. Structural basis for the functional differences between type I and type II human methionine aminopeptidases. *Biochemistry* **44**, 14741–14749 (2005). 16274222 10.1021/bi051691k

[8] Xiao, Q., Zhang, F., Nacev, B. A., Liu, J. O. & Pei, D. Protein N-terminal processing: substrate specificity of Escherichia coli and human methionine aminopeptidases. *Biochemistry* **49**, 5588–5599 (2010). 20521764 10.1021/bi1005464 PMC2906754

[9] Klein, M. A., Wild, K., Kišonaitė, M. & Sinning, I. Methionine aminopeptidase 2 and its autoproteolysis product have different binding sites on the ribosome. *Nat. Commun.* **15**, 716 (2024). 38267453 10.1038/s41467-024-44862-7 PMC10808355

[10] Wild, K. et al. MetAP-like Ebp1 occupies the human ribosomal tunnel exit and recruits flexible rRNA expansion segments. *Nat. Commun.* **11**, 776 (2020). 32034140 10.1038/s41467-020-14603-7 PMC7005732

[11] Kowalinski, E. et al. The crystal structure of Ebp1 reveals a methionine aminopeptidase fold as binding platform for multiple interactions. *FEBS Lett.* **581**, 4450–4454 (2007). 17765895 10.1016/j.febslet.2007.08.024

[12] Wang, Y. et al. The structure and function of multifunctional protein ErbB3 binding protein 1 (Ebp1) and its role in diseases. *Cell Biol. Int.* **48**, 1069–1079 (2024). 38884348 10.1002/cbin.12196

[13] Bhaskar, V. et al. Dynamic association of human Ebp1 with the ribosome. *RNA* **27**, 411–419 (2021). 33479117 10.1261/rna.077602.120 PMC7962488

[14] Kraushar, M. L. et al. Protein synthesis in the developing neocortex at near-atomic resolution reveals Ebp1-mediated neuronal proteostasis at the 60S tunnel exit. *Mol. Cell* **81**, 304–322.e316 (2021). 33357414 10.1016/j.molcel.2020.11.037 PMC8163098

[15] Lyons, P. J. Inactive metallopeptidase homologs: the secret lives of pseudopeptidases. *Front. Mol. Biosci.* **11**, 1436917 (2024). 39050735 10.3389/fmolb.2024.1436917 PMC11266112

[16] Aksnes, H., Drazic, A., Marie, M. & Arnesen, T. First things first: vital protein marks by N-terminal acetyltransferases. *Trends Biochem. Sci.* **41**, 746–760 (2016). 27498224 10.1016/j.tibs.2016.07.005

[17] Klein, M., Wild, K. & Sinning, I. Multi-protein assemblies orchestrate co-translational enzymatic processing on the human ribosome. *Nat. Commun.* **15**, 7681 (2024). 39227397 10.1038/s41467-024-51964-9 PMC11372111

[18] Knorr, A. G. et al. Ribosome-NatA architecture reveals that rRNA expansion segments coordinate N-terminal acetylation. *Nat. Struct. Mol. Biol.* **26**, 35–39 (2019). 30559462 10.1038/s41594-018-0165-y

[19] Knorr, A. G. et al. The dynamic architecture of Map1- and NatB-ribosome complexes coordinates the sequential modifications of nascent polypeptide chains. *PLoS Biol.* **21**, e3001995 (2023). 37079644 10.1371/journal.pbio.3001995 PMC10118133

[20] Arnesen, T. et al. Proteomics analyses reveal the evolutionary conservation and divergence of N-terminal acetyltransferases from yeast and humans. *Proc. Natl. Acad. Sci. USA* **106**, 8157–8162 (2009). 19420222 10.1073/pnas.0901931106 PMC2688859

[21] Gottlieb, L. & Marmorstein, R. Structure of human NatA and its regulation by the Huntingtin interacting protein HYPK. *Structure* **26**, 925–935 (2018). 29754825 10.1016/j.str.2018.04.003 PMC6031454

[22] Weyer, F. A. et al. Structural basis of HypK regulating N-terminal acetylation by the NatA complex. *Nat. Commun.* **8**, 15726 (2017). 28585574 10.1038/ncomms15726 PMC5467210

[23] Vetting, M. W. et al. Structure and functions of the GNAT superfamily of acetyltransferases. *Arch. Biochem Biophys.* **433**, 212–226 (2005). 15581578 10.1016/j.abb.2004.09.003

[24] Salah Ud-Din, A. I., Tikhomirova, A. & Roujeinikova, A. Structure and functional diversity of GCN5-related N-acetyltransferases (GNAT). *Int. J. Mol. Sci.* **17**, 1018 (2016). 10.3390/ijms17071018 PMC4964394 27367672

[25] Liszczak, G. et al. Molecular basis for N-terminal acetylation by the heterodimeric NatA complex. *Nat. Struct. Mol. Biol.* **20**, 1098–1105 (2013). 23912279 10.1038/nsmb.2636 PMC3766382

[26] Gautschi, M. et al. The yeast N(alpha)-acetyltransferase NatA is quantitatively anchored to the ribosome and interacts with nascent polypeptides. *Mol. Cell Biol.* **23**, 7403–7414 (2003). 14517307 10.1128/MCB.23.20.7403-7414.2003 PMC230319

[27] Evjenth, R. et al. Human Naa50p (Nat5/San) displays both protein N alpha- and N epsilon-acetyltransferase activity. *J. Biol. Chem.* **284**, 31122–31129 (2009). 19744929 10.1074/jbc.M109.001347 PMC2781511

[28] Arnesen, T. et al. The chaperone-like protein HYPK acts together with NatA in cotranslational N-terminal acetylation and prevention of Huntingtin aggregation. *Mol. Cell Biol.* **30**, 1898–1909 (2010). 20154145 10.1128/MCB.01199-09 PMC2849469

[29] Deng, S., McTiernan, N., Wei, X., Arnesen, T. & Marmorstein, R. Molecular basis for N-terminal acetylation by human NatE and its modulation by HYPK. *Nat. Commun.* **11**, 818 (2020). 32042062 10.1038/s41467-020-14584-7 PMC7010799

[30] Gong, X. et al. OsHYPK-mediated protein N-terminal acetylation coordinates plant development and abiotic stress responses in rice. *Mol. Plant* **15**, 740–754 (2022). 35381198 10.1016/j.molp.2022.03.001

[31] Miklánková, P. et al. HYPK promotes the activity of the N(α)-acetyltransferase A complex to determine proteostasis of nonAc-X(2)/N-degron-containing proteins. *Sci. Adv.* **8**, eabn6153 (2022). 35704578 10.1126/sciadv.abn6153 PMC9200280

[32] Lentzsch, A. M. et al. NAC guides a ribosomal multienzyme complex for nascent protein processing. *Nature* **633**, 18–724 (2024). 39169182 10.1038/s41586-024-07846-7 PMC12039536

[33] Raue, U., Oellerer, S. & Rospert, S. Association of protein biogenesis factors at the yeast ribosomal tunnel exit is affected by the translational status and nascent polypeptide sequence. *J. Biol. Chem.* **282**, 7809–7816 (2007). 17229726 10.1074/jbc.M611436200

[34] Deuerling, E., Gamerdinger, M. & Kreft, S. G. Chaperone interactions at the ribosome. *Cold Spring Harb. Perspect. Biol.* **11**, a033977 (2019). 10.1101/cshperspect.a033977 PMC6824243 30833456

[35] Pech, M., Spreter, T., Beckmann, R. & Beatrix, B. Dual binding mode of the nascent polypeptide-associated complex reveals a novel universal adapter site on the ribosome. *J. Biol. Chem.* **285**, 19679–19687 (2010). 20410297 10.1074/jbc.M109.092536 PMC2885246

[36] Kirstein-Miles, J., Scior, A., Deuerling, E. & Morimoto, R. I. The nascent polypeptide-associated complex is a key regulator of proteostasis. *EMBO J.* **32**, 1451–1468 (2013). 23604074 10.1038/emboj.2013.87 PMC3655472

[37] Gamerdinger, M. et al. Early scanning of nascent polypeptides inside the ribosomal tunnel by NAC. *Mol. Cell* **75**, 996–1006.e1008 (2019). 31377116 10.1016/j.molcel.2019.06.030

[38] Nyathi, Y. & Pool, M. R. Analysis of the interplay of protein biogenesis factors at the ribosome exit site reveals new role for NAC. *J. Cell Biol.* **210**, 287–301 (2015). 26195668 10.1083/jcb.201410086 PMC4508901

[39] Gamerdinger, M. et al. NAC controls cotranslational N-terminal methionine excision in eukaryotes. *Science* **380**, 1238–1243 (2023). 37347872 10.1126/science.adg3297

[40] Jomaa, A. et al. Mechanism of signal sequence handover from NAC to SRP on ribosomes during ER-protein targeting. *Science* **375**, 839–844 (2022). 35201867 10.1126/science.abl6459 PMC7612438

[41] Beatrix, B., Sakai, H. & Wiedmann, M. The alpha and beta subunit of the nascent polypeptide-associated complex have distinct functions. *J. Biol. Chem.* **275**, 37838–37845 (2000). 10982809 10.1074/jbc.M006368200

[42] Wiedmann, B., Sakai, H., Davis, T. A. & Wiedmann, M. A protein complex required for signal-sequence-specific sorting and translocation. *Nature* **370**, 434–440 (1994). 8047162 10.1038/370434a0

[43] Lauring, B., Sakai, H., Kreibich, G. & Wiedmann, M. Nascent polypeptide-associated complex protein prevents mistargeting of nascent chains to the endoplasmic reticulum. *Proc. Natl. Acad. Sci. USA* **92**, 5411–5415 (1995). 7777521 10.1073/pnas.92.12.5411 PMC41704

[44] Hou, F., Chu, C. W., Kong, X., Yokomori, K. & Zou, H. The acetyltransferase activity of San stabilizes the mitotic cohesin at the centromeres in a shugoshin-independent manner. *J. Cell Biol.* **177**, 587–597 (2007). 17502424 10.1083/jcb.200701043 PMC2064205

[45] Zheng, W. et al. Visualizing the translation landscape in human cells at high resolution. *Nat Commun.* **16**, 10757 (2025). 10.1038/s41467-025-65795-9 PMC12663405 41315256

[46] Kišonaitė, M. et al. Structural inventory of cotranslational protein folding by the eukaryotic RAC complex. *Nat. Struct. Mol. Biol.* **30**, 670–677 (2023). 37081320 10.1038/s41594-023-00973-1 PMC10191838

[47] Lewis, A. J. O., Zhong, F., Keenan, R. J. & Hegde, R. S. Structural analysis of the dynamic ribosome-translocon complex. *Elife* **13**, RP95814 (2024). 10.7554/eLife.95814 PMC11186639 38896445

[48] Arnesen, T. et al. A novel human NatA Nalpha-terminal acetyltransferase complex: hNaa16p-hNaa10p (hNat2-hArd1). *BMC Biochem.* **10**, 15 (2009). 19480662 10.1186/1471-2091-10-15 PMC2695478

[49] Weidenhausen, J. et al. Structural and functional characterization of the N-terminal acetyltransferase Naa50. *Structure* **29**, 413–425.e415 (2021). 33400917 10.1016/j.str.2020.12.004

[50] Deng, S. et al. Structure and mechanism of acetylation by the N-terminal dual enzyme NatA/Naa50 complex. *Structure* **27**, 1057–1070 (2019). 31155310 10.1016/j.str.2019.04.014 PMC6610660

[51] Krissinel, E. & Henrick, K. Inference of macromolecular assemblies from crystalline state. *J. Mol. Biol.* **372**, 774–797 (2007). 17681537 10.1016/j.jmb.2007.05.022

[52] Cheng, H. et al. Phenotypic and biochemical analysis of an international cohort of individuals with variants in NAA10 and NAA15. *Hum. Mol. Genet.* **28**, 2900–2919 (2019). 31127942 10.1093/hmg/ddz111 PMC6736318

[53] Weidenhausen, J. et al. Extended N-terminal acetyltransferase Naa50 in filamentous fungi adds to Naa50 diversity. *Int. J. Mol. Sci.* **23**, 10805 (2022). 10.3390/ijms231810805 PMC9500918 36142717

[54] Greber, B. J., Boehringer, D., Montellese, C. & Ban, N. Cryo-EM structures of Arx1 and maturation factors Rei1 and Jjj1 bound to the 60S ribosomal subunit. *Nat. Struct. Mol. Biol.* **19**, 1228–1233 (2012). 23142985 10.1038/nsmb.2425

[55] Bradatsch, B. et al. Arx1 functions as an unorthodox nuclear export receptor for the 60S preribosomal subunit. *Mol. Cell* **27**, 767–779 (2007). 17803941 10.1016/j.molcel.2007.06.034

[56] Pettersen, E. F. et al. UCSF ChimeraX: structure visualization for researchers, educators, and developers. *Protein Sci.* **30**, 70–82 (2021). 32881101 10.1002/pro.3943 PMC7737788

[57] Natchiar, S. K., Myasnikov, A. G., Kratzat, H., Hazemann, I. & Klaholz, B. P. Visualization of chemical modifications in the human 80S ribosome structure. *Nature* **551**, 472–477 (2017). 29143818 10.1038/nature24482

[58] Emsley, P., Lohkamp, B., Scott, W. G. & Cowtan, K. Features and development of Coot. *Acta Crystallogr. D Biol. Crystallogr.* **66**, 486–501 (2010). 20383002 10.1107/S0907444910007493 PMC2852313

[59] Afonine, P. V. et al. Real-space refinement in PHENIX for cryo-EM and crystallography. *Acta Crystallogr. D Struct. Biol.* **74**, 531–544 (2018). 29872004 10.1107/S2059798318006551 PMC6096492

[60] Adams, P. D. et al. PHENIX: a comprehensive Python-based system for macromolecular structure solution. *Acta Crystallogr. D Biol. Crystallogr.* **66**, 213–221 (2010). 20124702 10.1107/S0907444909052925 PMC2815670

[61] Goedhart, J. & Luijsterburg, M. S. VolcaNoseR is a web app for creating, exploring, labeling and sharing volcano plots. *Sci. Rep.* **10**, 20560 (2020). 33239692 10.1038/s41598-020-76603-3 PMC7689420

[62] Perez-Riverol, Y. et al. PRIDE inspector toolsuite: moving toward a universal visualization tool for proteomics data standard formats and quality assessment of ProteomeXchange datasets. *Mol. Cell Proteom.* **15**, 305–317 (2016). 10.1074/mcp.O115.050229 PMC4762524 26545397

