# Supplementary material for: NatA engages in multi-factor complexes at the ribosomal polypeptide tunnel exit
Source: Nat Commun. 2026 Jan 23;17:884. doi: 10.1038/s41467-026-68787-5 (PMC12830823; doi:10.1038/s41467-026-68787-5)
Supplement: Supplementary file 1 — Supplementary Information [file 41467_2026_68787_MOESM1_ESM.pdf]

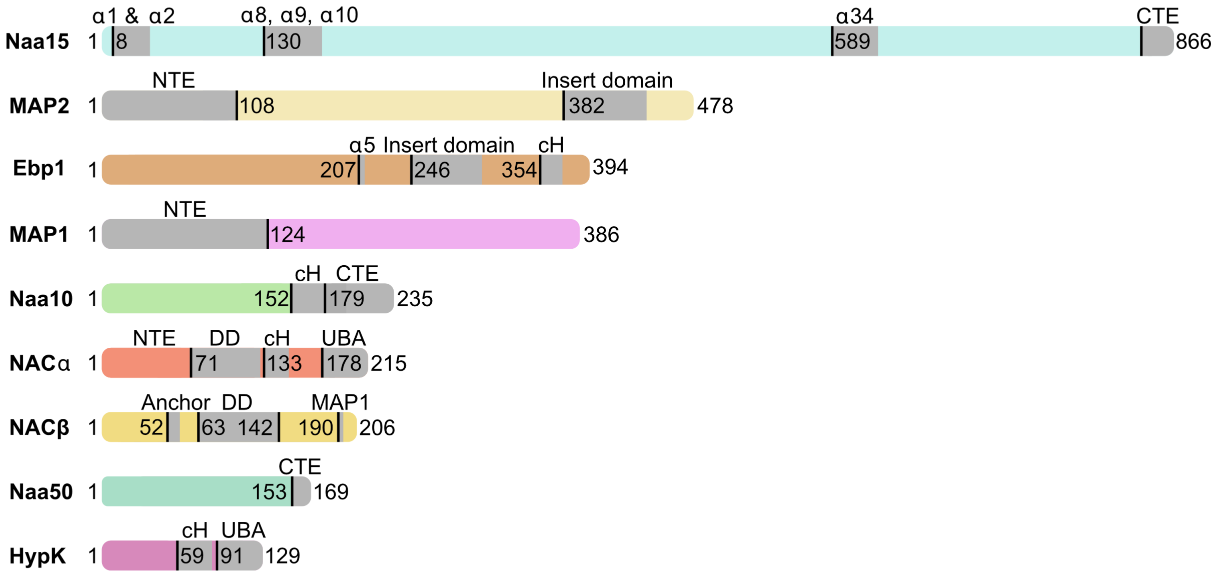

Supplementary Figure 1: Domain organization of ribosome associated proteins. Important features are labelled. Residue numbers are also shown and marked with a black line. Important α-helical elements are marked, as well as the insert domains of MAP2 and Ebp1. In addition, the contact helices (cH) involved in the Naa15 interactions are highlighted, as well as unstructured N-terminal extensions (NTE) or C-terminal extensions (CTE). The dimerization domain (DD) of NAC and UBA domains of NACα and HypK are also labelled. For NACβ, the anchoring helix and MAP1 contact site are also shown.

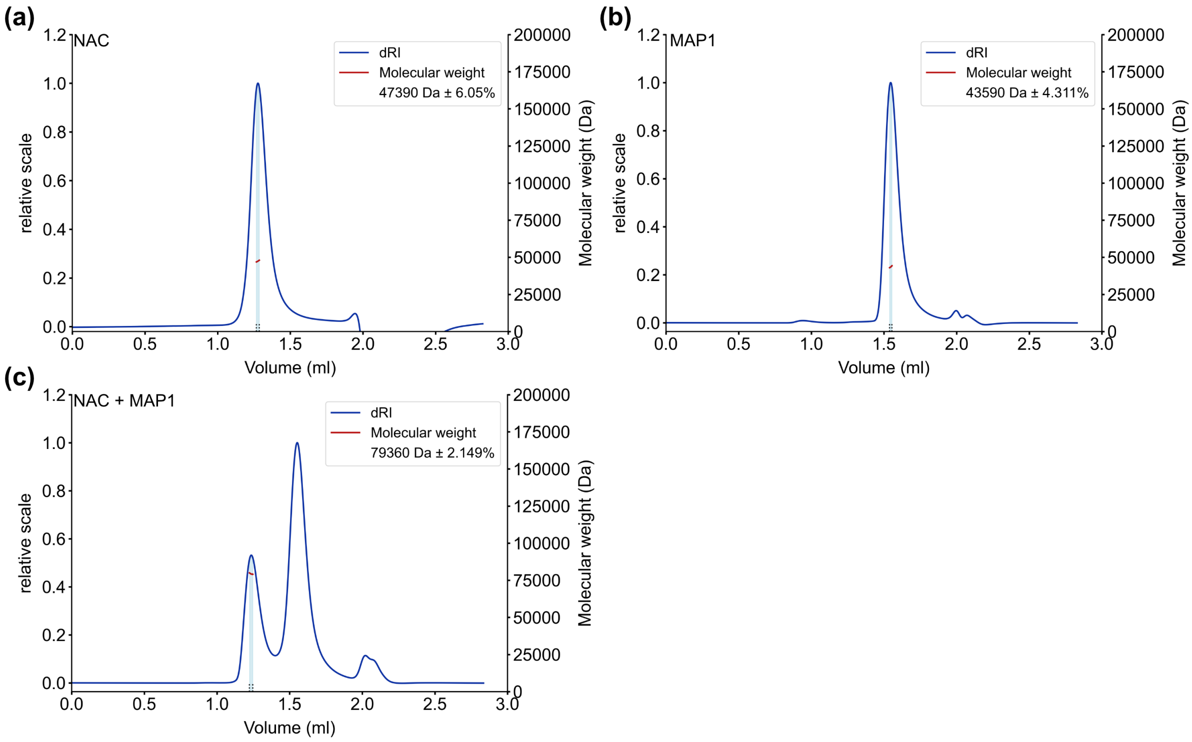

Supplementary Figure 2: SEC-MALS confirms the formation of stable NAC-MAP1 complexes. (a) Without MAP1, the molecular weight of NAC was determined to be ~47 kDa (theoretical ~46 kDa), corresponding to a single heterodimer of NAC. **(b)** Without NAC, the molecular weight of MAP1 was determined to be ~44 kDa (theoretical ~44 kDa), corresponding to a monomer. **(c)** When mixing MAP1 and NAC, a 1:1 complex of ~79 kDa is formed between the two proteins. SEC runs were done on an S200 Increase 3.2/300 column (Cytiva). Source data are provided as a source data file.

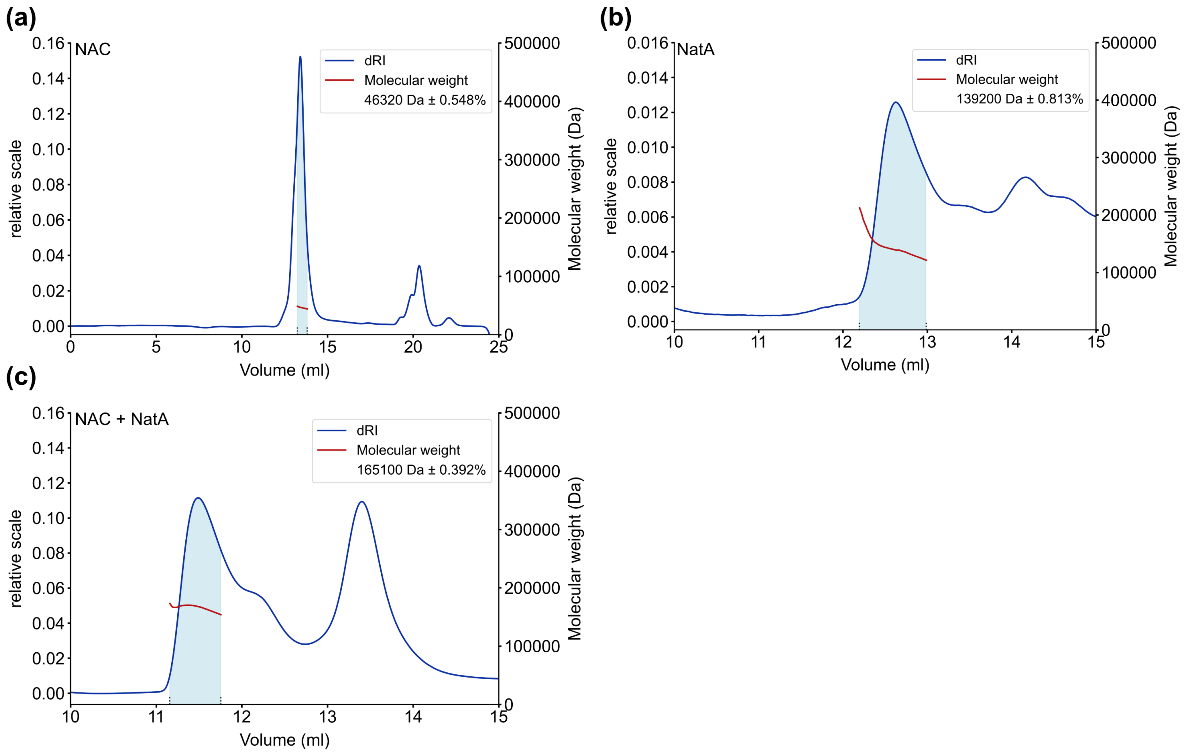

Supplementary Figure 3: SEC-MALS confirms the formation of stable NAC-NatA complexes. (a) Without NatA, the molecular weight of NAC was determined to be ~46 kDa (theoretical ~46 kDa), corresponding to a single heterodimer of NAC. **(b)** Without NAC, the molecular weight of NatA was determined to be ~139 kDa (theoretical ~129 kDa), corresponding to a single heterodimer of NatA. **(c)** When mixing NatA and NAC, complex formation increases the molecular weight to ~165 kDa. SEC runs were done on an S200 Increase 10/300 GL column (Cytiva). Source data are provided as a source data file.

(a)

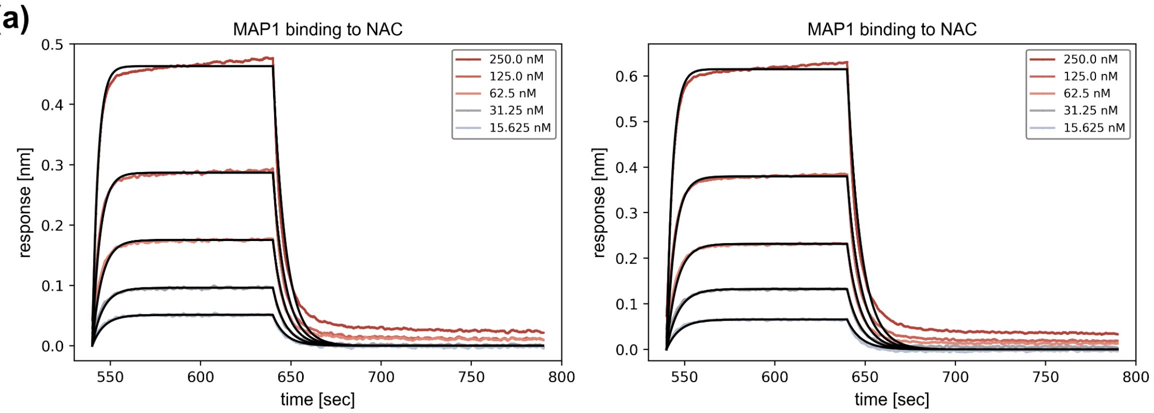

Replicate	K_d	K_d Error	k_a	k_a Error	k_{dis}	k_{dis} Error	Full R^2
1	200.8 nM	4.607	716700 Ms^{-1}	15760	0.1439 s^{-1}	0.0009430	0.9927
2	191.7 nM	4.317	676400 Ms^{-1}	14580	0.1297 s^{-1}	0.0008419	0.9920

(b)

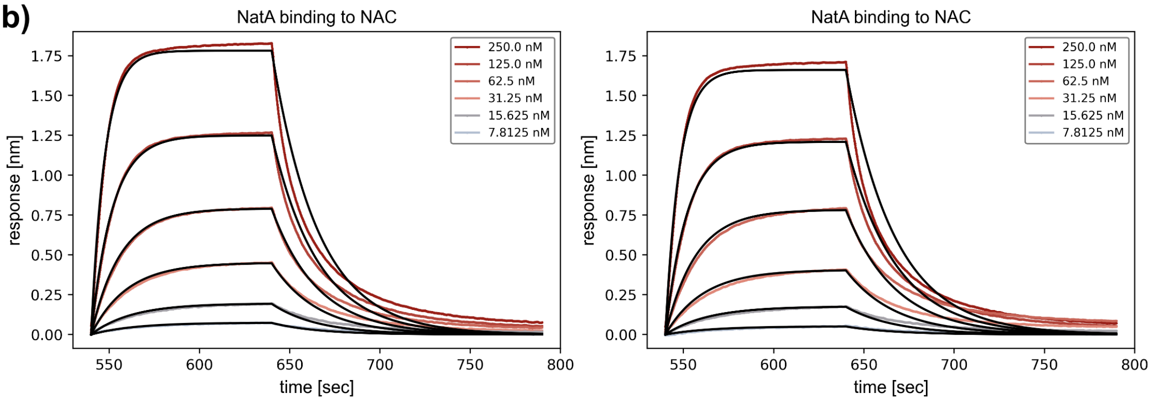

Replicate	K_d	K_d Error	k_a	k_a Error	k_{dis}	k_{dis} Error	Full R^2
1	107.9 nM	1.151	363200 Ms^{-1}	3701	0.03919 s^{-1}	0.000124	0.9929
2	101.0 nM	1.284	363200 Ms^{-1}	4411	0.03667 s^{-1}	0.000138	0.9891

(c) (d)

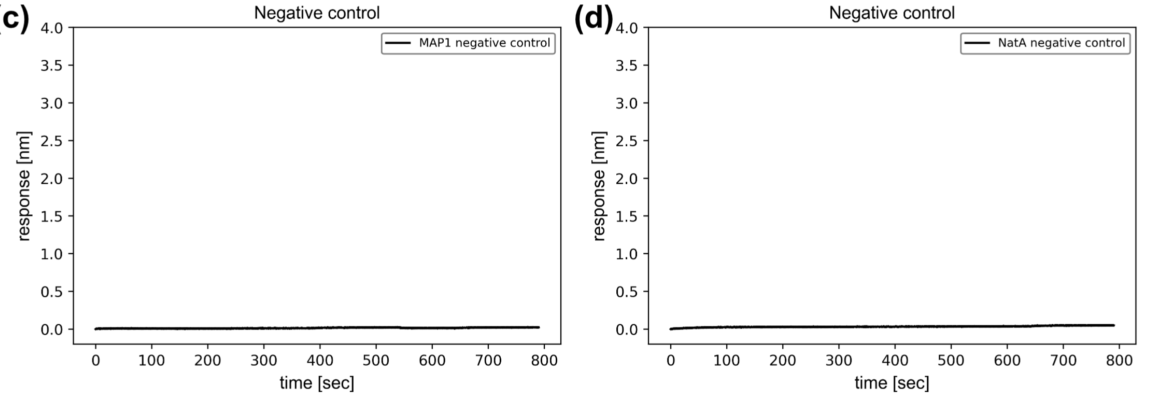

Figure description is located on the next page

Supplementary Figure 4: BLI kinetic measurements of MAP1 and NatA binding to immobilized NAC. For each measurement, biotinylated NAC was loaded to the sensor tip and washed twice in BLI buffer (not shown). Then, different concentrations of analyte (MAP1 or NatA) were associated to the sensor tips for 100 seconds. Dissociation was carried out over 150 seconds in BLI buffer. Analyte concentrations ranged from 250 nM to ~15.6 nM for MAP1 and from 250 nM to ~7.8 nM for NatA (colored from red to blue). Fitted curves are overlayed in black. Measurements were done in duplicates (n=2). K_d, ka, kdis and errors, as well as the coefficient of determination R^2 are shown in the table below. **(a)** MAP1 binding to NAC. **(b)** NatA binding to NAC. Two controls were run in parallel to ensure that neither **(c)** MAP1, nor **(d)** NatA bind unspecifically to unloaded SA-biosensors. Source data are provided as a source data file.

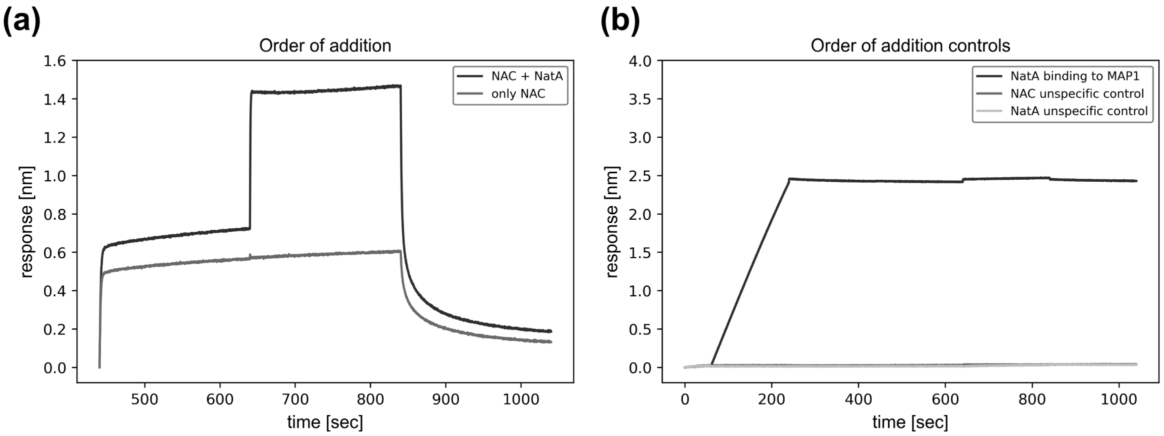

Supplementary Figure 5: NAC mediates the interaction between MAP1 and NatA. (a) Biotinylated MAP1 was loaded to the sensor tips and washed twice with BLI buffer (not shown). Then NAC was associated (440-640 sec). A control sensor (dark grey) was moved into a new well containing the same concentration of NAC (640-840 sec) and finally moved into BLI buffer for dissociation (840-1040). As expected, the second association step (640-840 sec) did not result in an additional sudden increase in response. In the main measurement (black), NAC was also associated to the MAP1 loaded sensor tips (440-640 sec). For the second association step however, the biosensor was moved into a NAC-NatA containing well (640-840 sec), resulting in the rapid association of NatA to the MAP1 loaded sensor. **(b)** To show that NatA does not rapidly bind to MAP1 without NAC, NatA was associated to a MAP1 loaded biosensor (dark grey) (640-840 sec). Additional controls (grey and light-grey) were done to ensure that neither NAC, not NatA bind unspecifically to unloaded SA-biosensors. Source data are provided as a source data file.

(a)

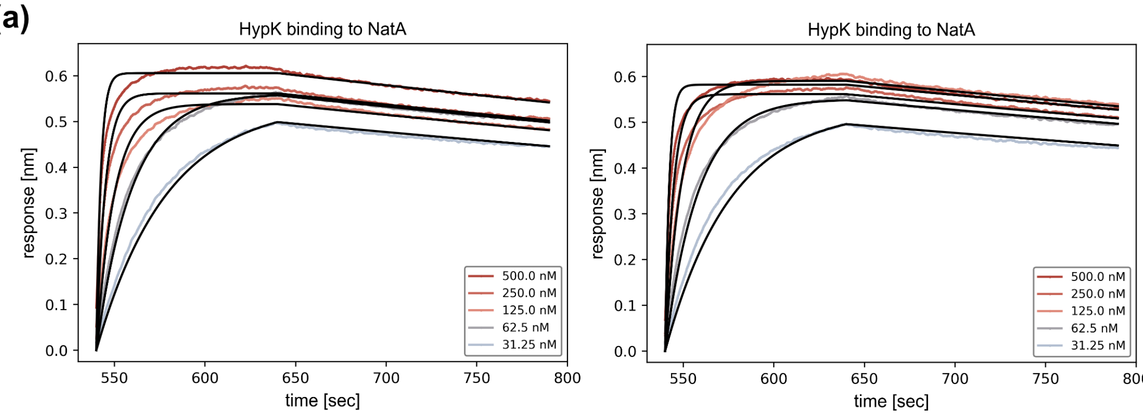

Replicate	K_d	K_d Error	k_a	k_a Error	k_{dis}	k_{dis} Error	Full R^2
1	0.9341 nM	0.008455	800100 Ms^{-1}	2661	0.000747 s^{-1}	0.000006	0.9830
2	0.7652 nM	0.009155	862200 Ms^{-1}	3427	0.00066 s^{-1}	0.000007	0.9743

(b)

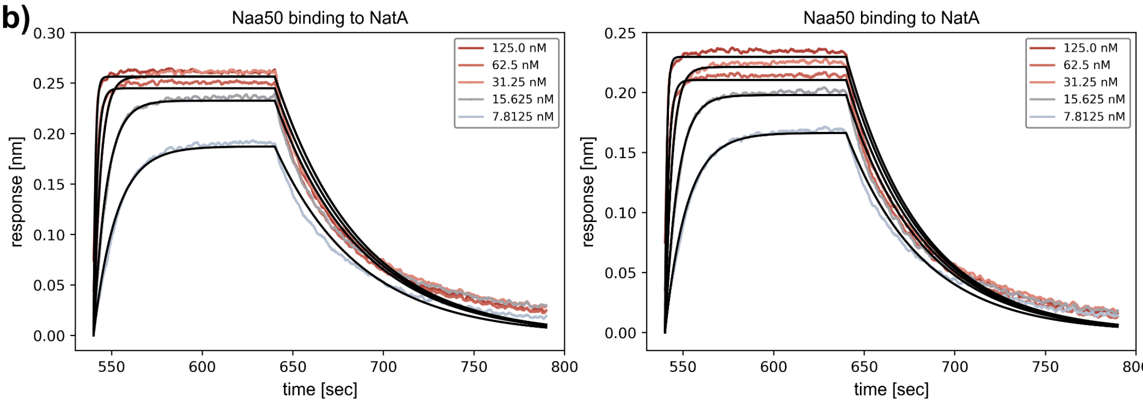

Replicate	K_d	K_d Error	k_a	k_a Error	k_{dis}	k_{dis} Error	Full R^2
1	22.37 nM	0.2327	946700 Ms^{-1}	9528	0.02118 s^{-1}	0.000056	0.9883
2	24.96 nM	0.2446	950400 Ms^{-1}	9004	0.02373 s^{-1}	0.000059	0.9907

(c)

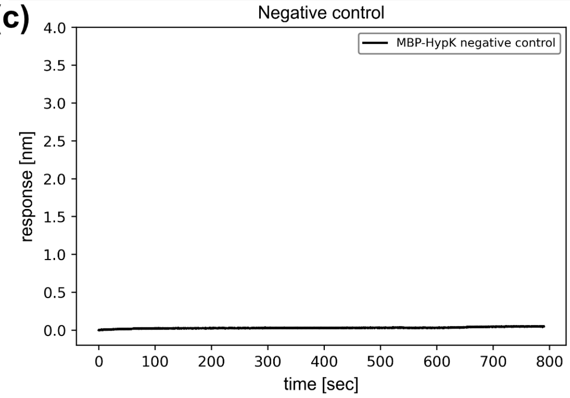

(d)

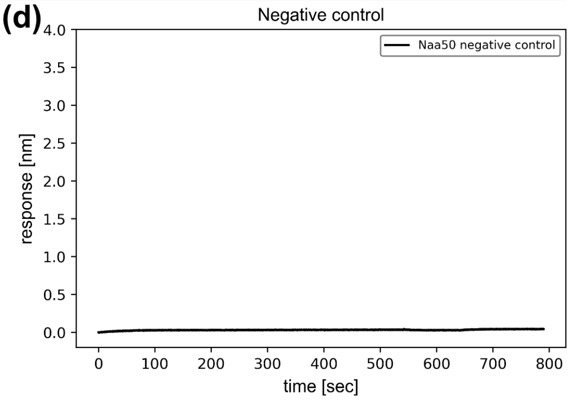

Figure description is located on the next page

Supplementary Figure 6: BLI kinetic measurements of MBP-HypK and Naa50 binding to immobilized NatA. For each measurement, biotinylated NatA was loaded to the sensor tip and washed twice in BLI buffer (not shown). Then, different concentrations of analyte (MBP-HypK or Naa50) were associated to the sensor tips for 100 seconds. Dissociation was carried out over 150 seconds in BLI buffer. Analyte concentrations ranged from 500 nM to 31.25 nM for MBP-HypK and from 125 nM to ~7.8 nM for Naa50 (colored from red to blue). Fitted curves are overlayed in black. Measurements were done in duplicates (n=2). K_d, ka, kdis and errors, as well as the coefficient of determination R^2 are shown in the table below. **(a)** MBP-HypK binding to NatA. **(b)** Naa50 binding to NatA. Two controls were run in parallel to ensure that neither **(c)** MBP-HypK, nor **(d)** Naa50 bind unspecifically to unloaded SA-biosensors. Source data are provided as a source data file.

(a)

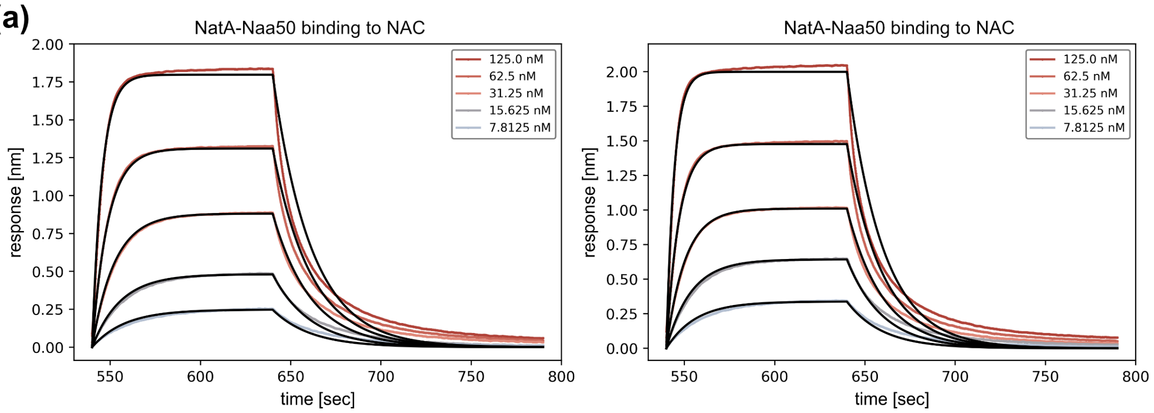

Replicate	K_d	K_d Error	k_a	k_a Error	k_{dis}	k_{dis} Error	Full R^2
1	177.0 nM	2.230	273200 Ms^{-1}	3275	0.04834 s^{-1}	0.000188	0.9920
2	140.8 nM	1.947	370500 Ms^{-1}	4869	0.05215 s^{-1}	0.000226	0.9906

(b)

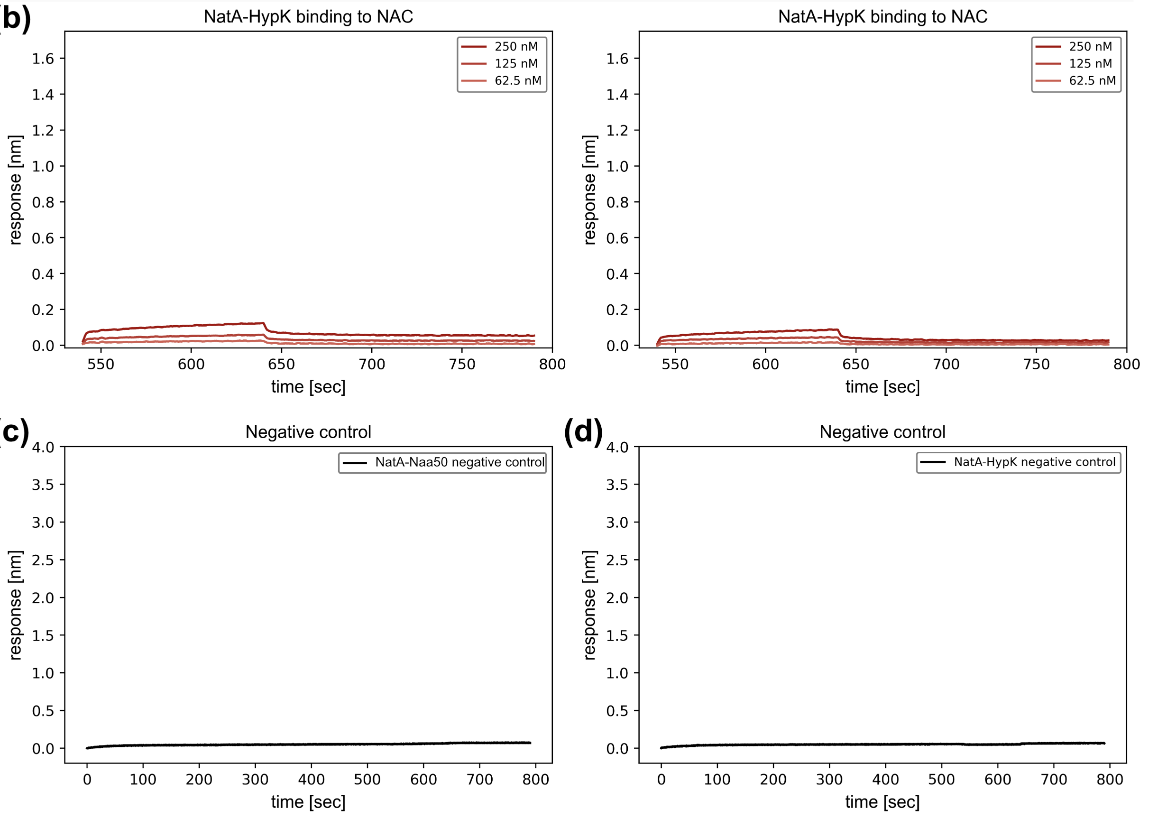

Figure description is located on the next page

Supplementary Figure 7: BLI measurements of NatE and NatA-HypK binding to immobilized NAC. For each measurement, biotinylated NAC was loaded to the sensor tip and washed twice in BLI buffer (not shown). Then, different concentrations of analyte (NatA-Naa50 or NatA-HypK) were associated to the sensor tips for 100 seconds. Dissociation was carried out over 150 seconds in BLI buffer. Analyte concentrations ranged from 125 nM to ~7.8 nM for NatE (1:1 NatA-Naa50) and from 250 nM to 62.5 nM for NatA-HypK (colored from red to blue). **(a)** NatA-Naa50 binding to NAC. Fitted curves are overlayed in black. Measurements were done in duplicates (n=2). K_d, ka, kdis and errors, as well as the coefficient of determination R^2 are shown in the table below. **(b)** NatA-HypK binding to NAC. Signal amplitude was severely diminished in the presence of HypK and resulting curves could no longer be fit with a global 1:1 model. Two controls were run in parallel to ensure that neither **(c)** NatA-Naa50, nor **(d)** NatA-HypK bind unspecifically to unloaded SA-biosensors. Source data are provided as a source data file.

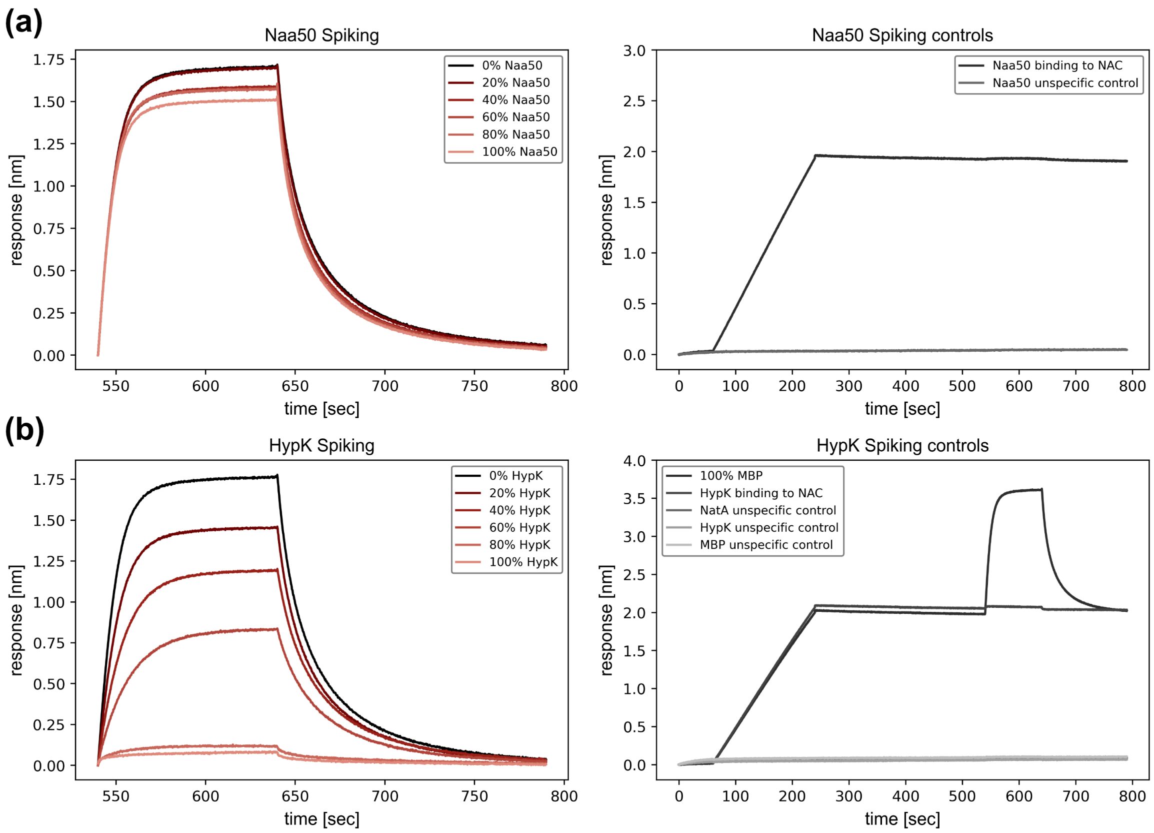

Supplementary Figure 8: BLI spiking experiment shows negative effect of Naa50 and HypK on the NAC-NatA interaction. For the main experiment (left panel) biotinylated NAC was loaded to six biosensors and washed twice with BLI buffer (not shown). Then, all sensors were moved into wells containing the same concentration of NatA. In addition to the consistent concentration of NatA, each well was supplemented with increasing concentrations of Naa50 or MBP-HypK (0%-100% with respect to the NatA concentration). **(a)** Increasing concentrations of Naa50 (up to 1:1 with NatA) slightly dampens the response of the NAC-NatA interaction (left panel). Two control sensors were used (right panel) to ensure that Naa50 does not associate to NAC loaded biosensor (association 540-640 sec) (black) and that Naa50 does not bind to unloaded SA-biosensors (dark grey). **(b)** Increasing concentrations of MBP-HypK (up to 1:1 with NatA) diminish the response of the NAC-NatA interaction (left panel). Five control sensors were used (right panel) The first control (100% MBP, black) was done to ensure that the MBP, which is also fused to HypK, does not negatively impact the NAC-NatA interaction. To do so, biotinylated NAC was loaded to sensor tips (60-240 sec) and wash twice in BLI buffer. Then NatA was associated (540-640 sec) (as in the main experiment) with the addition of MBP (1:1 with NatA). Finally, NatA was dissociated in BLI buffer. As expected, the presence of MBP did not negatively impact the NAC-NatA interaction. The second control (dark grey) shows that MBP-HypK does not bind to NAC loaded sensor tips. The remaining controls confirmed that there is no unspecific interaction between NatA, MBP-HypK or MBP to unloaded SA-biosensors. Source data are provided as a source data file.

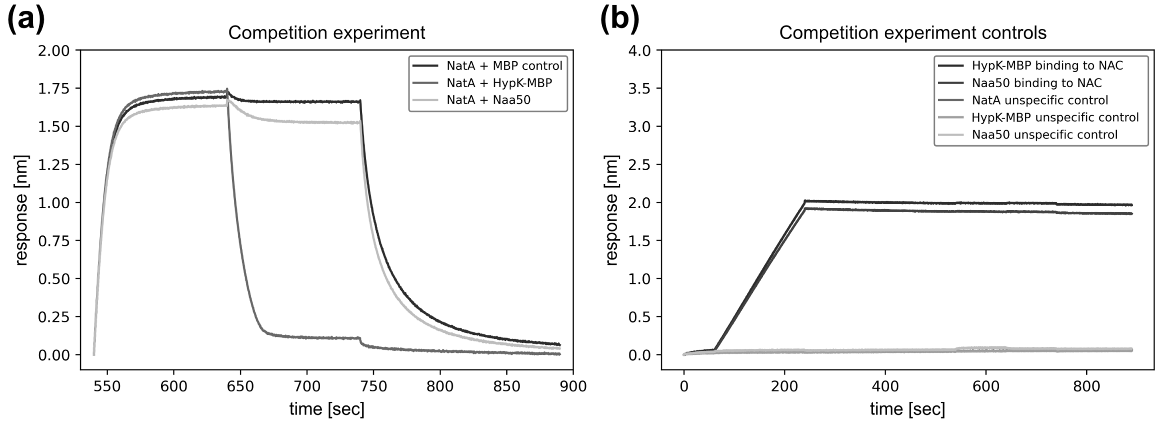

Supplementary Figure 9: BLI competition experiment shows that Naa50 and HypK negatively impact the NatA-NAC interaction. (a) Biosensors were loaded with biotinylated NAC and washed twice with BLI buffer (not shown). Then, NatA was associated to all three sensor tips (540-640 sec). To ensure that MBP does not negatively impact the NatA-NAC interaction, the biosensor was moved into a second NatA containing well with the addition of MBP (1:1 with NatA) (black). As expected, the presence of MBP did not dampen the NatA-NAC interaction. In the main experiment, NAC-loaded biosensors that were previously associated with NatA were moved into a new well that contained the same concentration of NatA, with the addition of MBP-HypK (grey) or Naa50 (light-grey). The presence of MBP-HypK results in the displacement of NAC and the rapid dissociation of NatA from the NAC loaded biosensor. The presence of Naa50 results in the dissociation of a small fraction of NatA from the NAC loaded biosensor. **(b)** Five controls sensors were used to ensure that MBP-HypK and Naa50 do not bind to NAC (black and dark grey). The remaining control sensors confirmed that there is no unspecific interaction between NatA, MBP-HypK or Naa50 with the unloaded SA-biosensor. Source data are provided as a source data file.

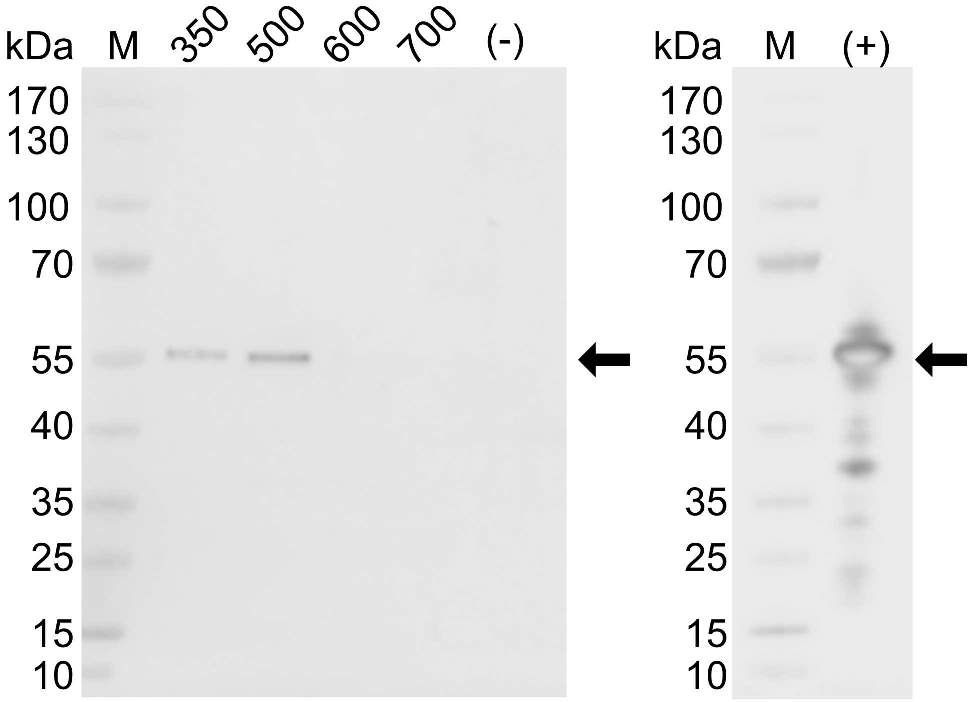

Supplementary Figure 10: Ebp1 remains ribosome associated at 500 mM salt. Purified HIS-Ebp1 was mixed with 80S ribosomes and subjected to SEC at different salt concentrations (350-700 mM KOAc) on a Superose 6 5/150 GL column (Cytiva). Ribosome peak fractions were subjected to SDS-PAGE and Immuno-blotting. The anti-HIS antibody clearly detects Ebp1 up to a salt concentration of 500 mM (left blot, third lane). Ribosomes without Ebp1 were used as a negative control (-). Purified HIS-Ebp1 without ribosomes was used as a positive control (+). The black arrow indicates the band corresponding to HIS-Ebp1. Source data are provided as a source data file.

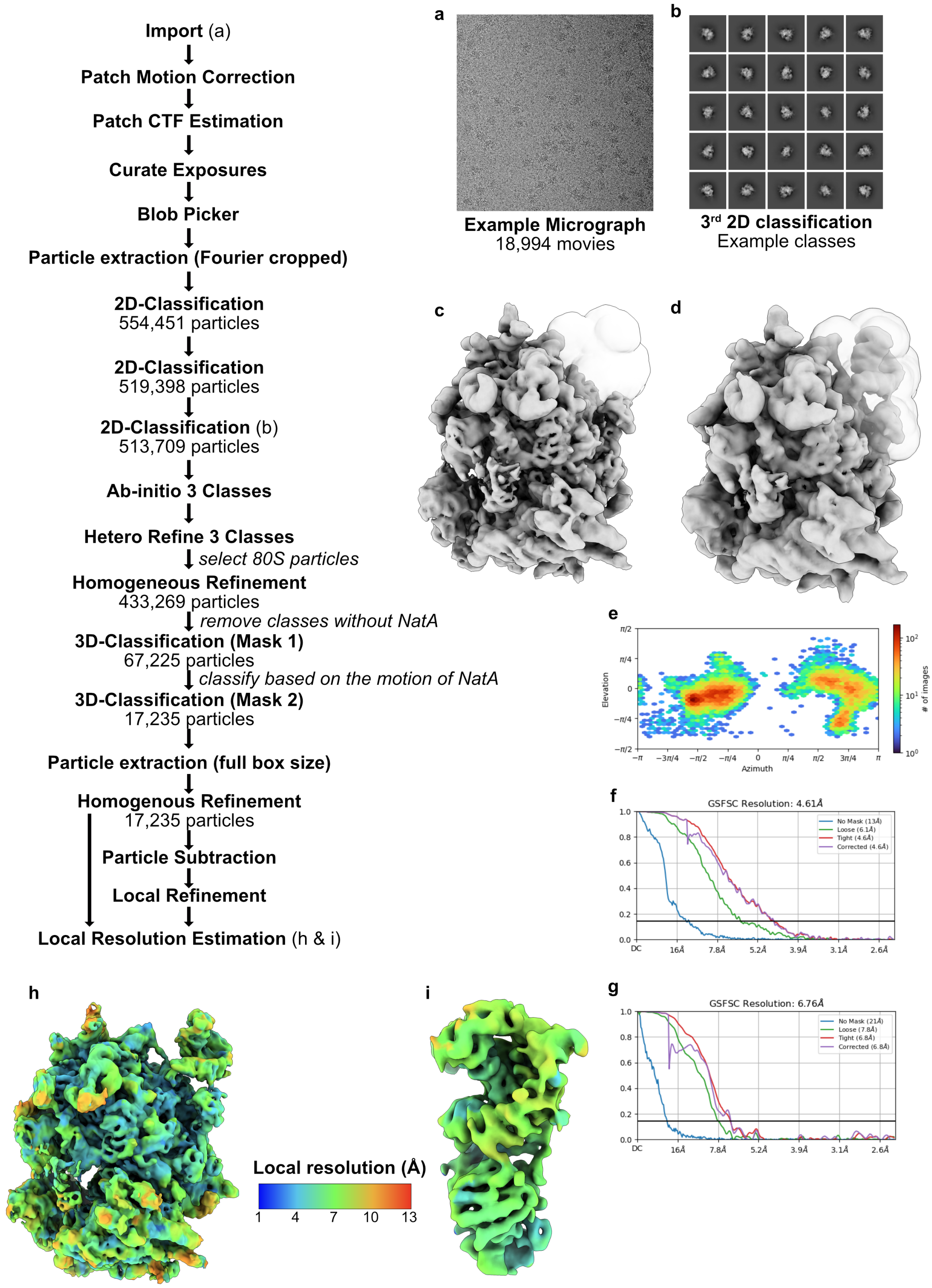

Figure description is located on the next page

Supplementary Figure 11: Cryo-EM data processing for the ternary NatA-Ebp1-80S complex. After data import **(a)** and pre-processing in cryoSPARC, particles were picked and subjected to three rounds of 2D classification **(b)**. Ribosome particles were selected and used to initialize an Ab-initio reconstruction into three classes. Resulting volumes were used to seed a Heterogeneous Refinement. 80S particles were subjected to Homogenous Refinement and masks were generated to encompass the binding site of distal NatA **(c)** as well as NatA-Ebp1 **(d)**. Masks were used for two subsequent 3D classifications. The first mask (c) was used to remove particles that do not have NatA, and the second mask (d) was used to select particles that showed a well-resolved interaction between NatA and the C-terminus of Ebp1. Remaining particles were subjected to Homogenous Refinement. **(e)** Angular distribution plot of the final Homogenous Refinement. **(f)** FSC plot of the final Homogenous Refinement. Finally, a mask was generated to remove the 80S signal with a particle subtraction job and a Local Refinement was run with the mask shown in (d). **(g)** FSC plot of the final Local Refinement. **(h)** Local resolution estimation of the final Homogenous Refinement and **(i)** the final Local Refinement.

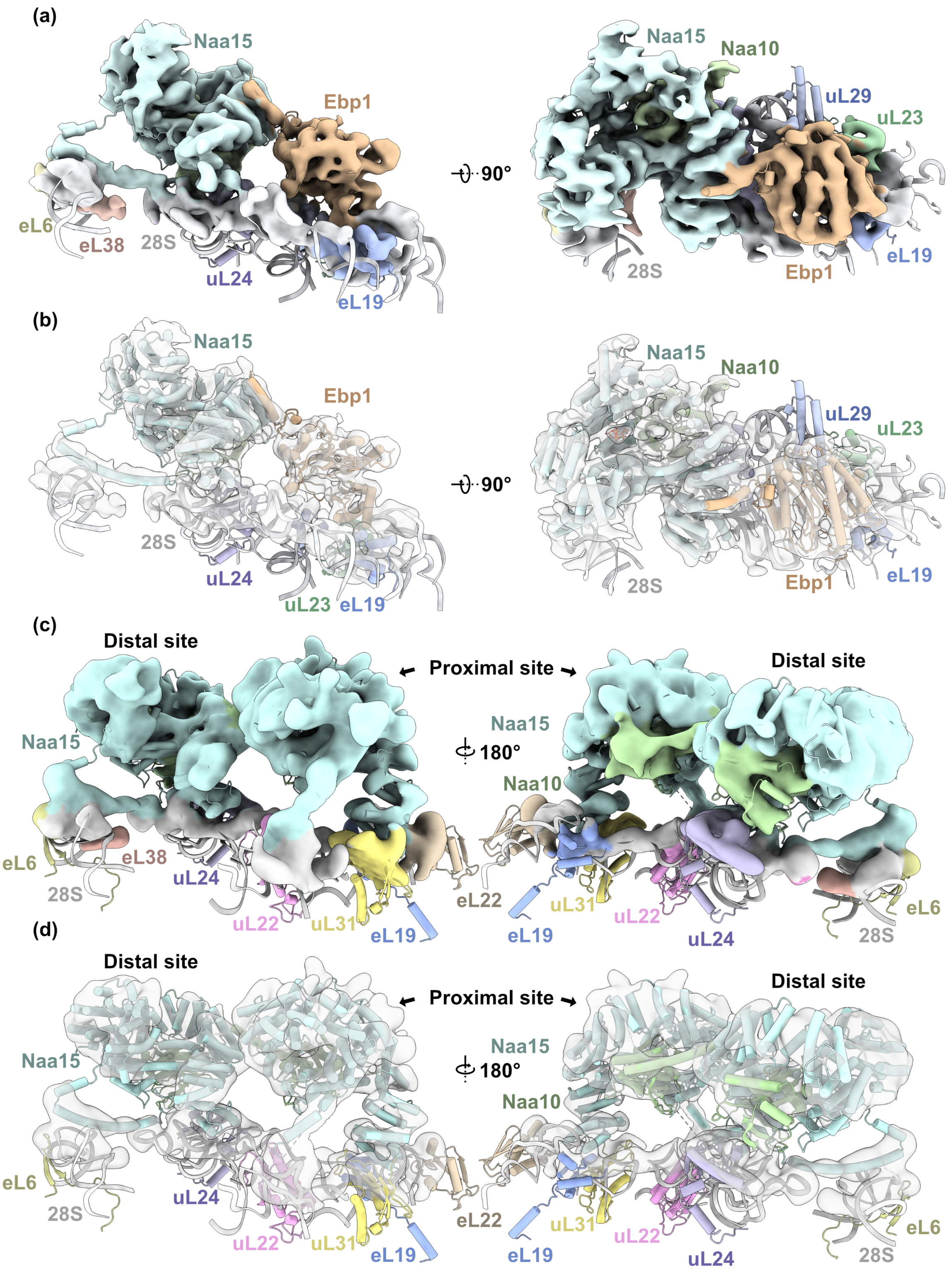

Figure description is located on the next page

Supplementary Figure 12: Map versus model for cryo-EM reconstructions of NatA-Ebp1 and NatA-NatA decorated ribosomes. (a) Coloured cryo-EM map from the final local Refinement overlayed on top of the Ebp1-NatA-80S model. **(b)** Same views as in (a) but with transparent map. **(c)** Front and back view on the NatA-NatA complex on human ribosomes. The map from the final local refinement is overlayed on top of the NatA-NatA-80S model. **(d)** Same views as in (c) but with transparent map.

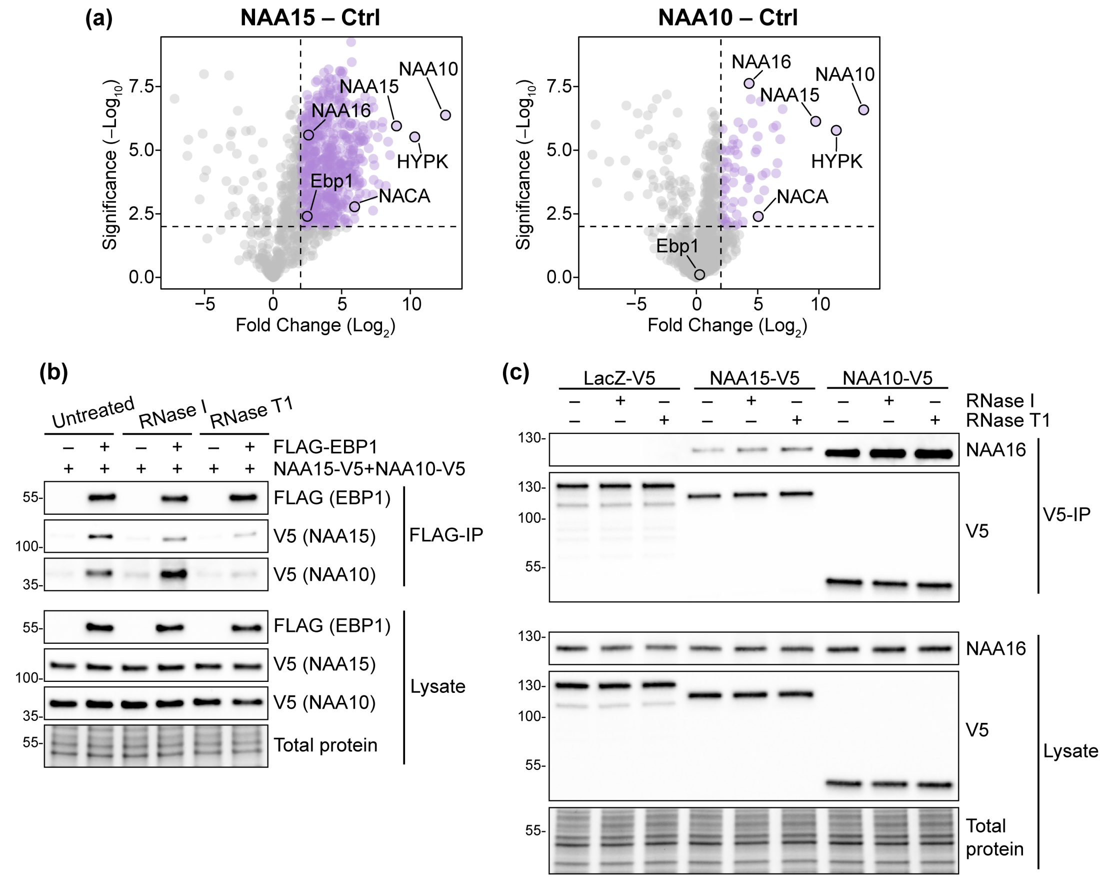

Supplementary Figure 13: Ebp1 and Naa16 are cellular binding partners of Naa15. (a) Volcano plots of proteins identified my mass spectrometry analysis in Naa15 and Naa10 immunoprecipitation (IP) samples from HeLa cells without prior RNAse treatment. Proteins enriched in Naa15 or Naa10 IP compared to control IP samples are indicated in purple. Significance threshold was $\log_2$ fold change > 2 and p-value < 0.01. Experiment was performed using four (n = 4) independent samples per condition. **(b)** Western blot analysis of FLAG-immunoprecipitation (IP) samples from HeLa cells co-transfected with FLAG-EBP1, NAA15-V5, and NAA10-V5. Control IPs had FLAG-EBP1 substituted with an empty vector. Cell lysates were treated with or without RNase I and T1 to dissolve polysomes. NAA15-V5 and NAA10-V5 showed enriched binding to FLAG-EBP1 compared to control IPs under all conditions. **(c)** Western blot analysis of V5-IP samples from HeLa cells transfected with NAA15-V5, NAA10-V5, or LacZ-V5 as a control. Endogenous NAA16 co-immunoprecipitated with NAA15-V5 and

NAA10-V5 in both RNase-treated and untreated cell lysates. Western blots are representative of three independent experiments (n=3). Source data are provided as a source data file.

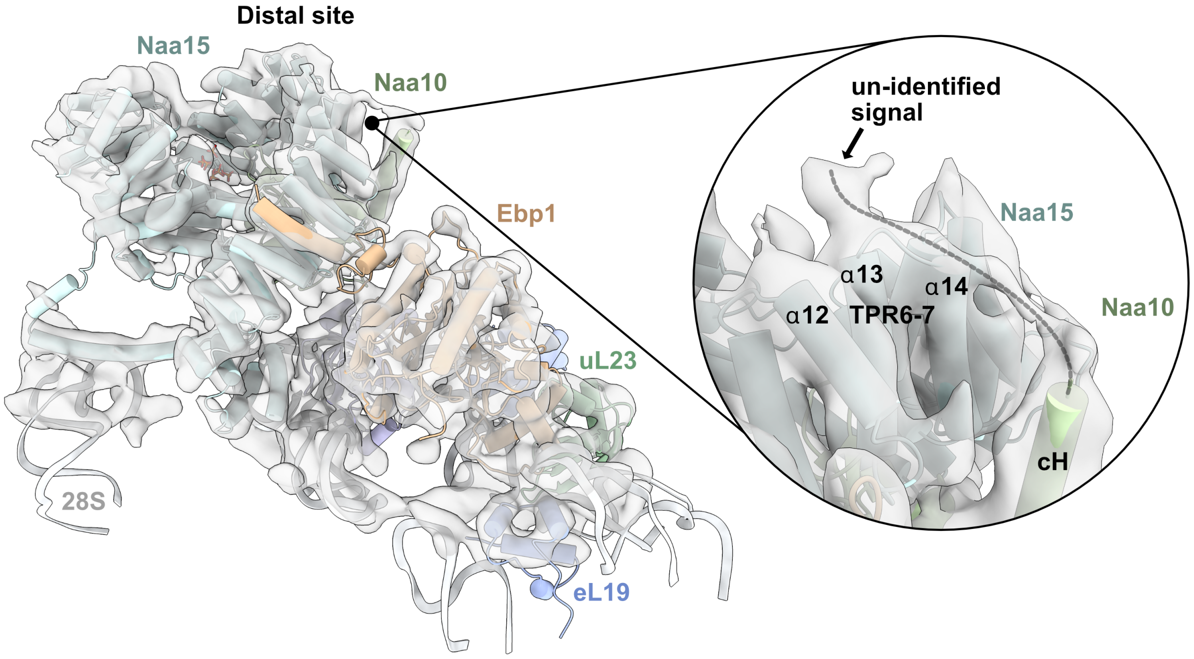

Supplementary Figure 14: The unstructured C-terminal extension of Naa10 appears to touch town on TPR6-7 of Naa15. Cryo-EM map from the final local refinement is overlayed on top of the NatA-Ebp1-80S model. The Naa10-cH is positioned in parallel to TPR6-7. The trajectory of Naa10s unstructured C-terminal extension is visible beyond the Naa10-cH and seems to interact with the top of Naa15 helixes α12-14 of TPR6-7.

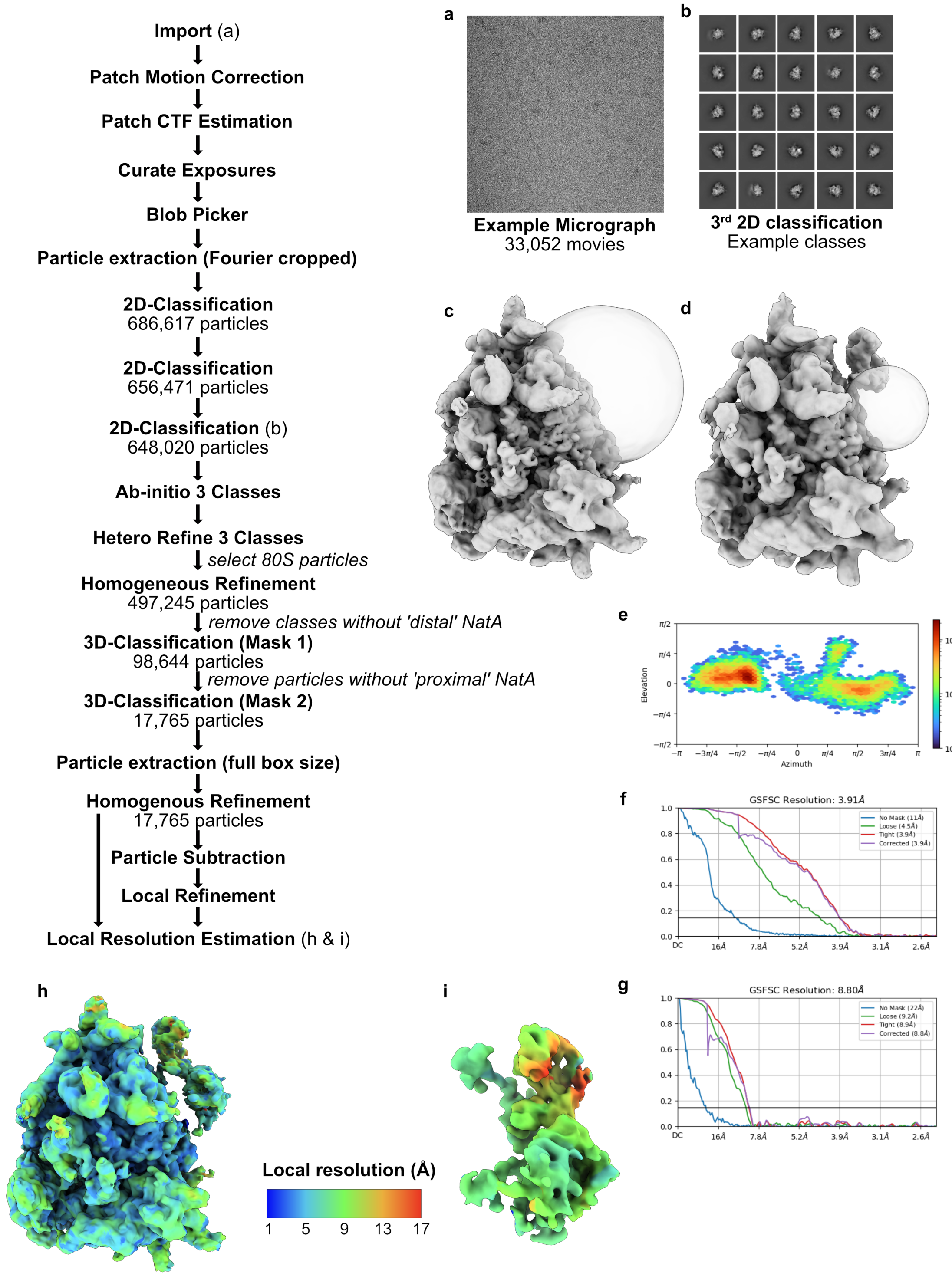

Figure description is located on the next page

Supplementary Figure 15: Cryo-EM data processing for the ternary NatA-NatA-80S complex. After data import **(a)** and pre-processing in cryoSPARC, particles were picked and subjected to three rounds of 2D classification **(b)**. Ribosome particles were selected and used to initialize an Ab-initio reconstruction into three classes. Resulting volumes were used to seed a Heterogeneous Refinement. 80S particles were subjected to Homogenous Refinement and a masks were generated to encompass the binding site of distal and proximal NatA **(c)** as well as specifically for proximal NatA **(d)**. Masks were used for two subsequent 3D classifications. The first mask (c) was used to remove particles that do not have distal NatA, and the second mask (d) was used to remove ribosomes without proximal NatA. Remaining particles were subjected to Homogenous Refinement. **(e)** Angular distribution plot of the final Homogenous Refinement. **(f)** FSC plot of the final Homogenous Refinement. Finally, a mask was generated to remove the 80S signal with a particle subtraction job and a Local Refinement was run with the mask shown in (c). **(g)** FSC plot of the final Local Refinement. **(h)** Local resolution estimation of the final Homogenous Refinement and **(i)** the final Local Refinement.

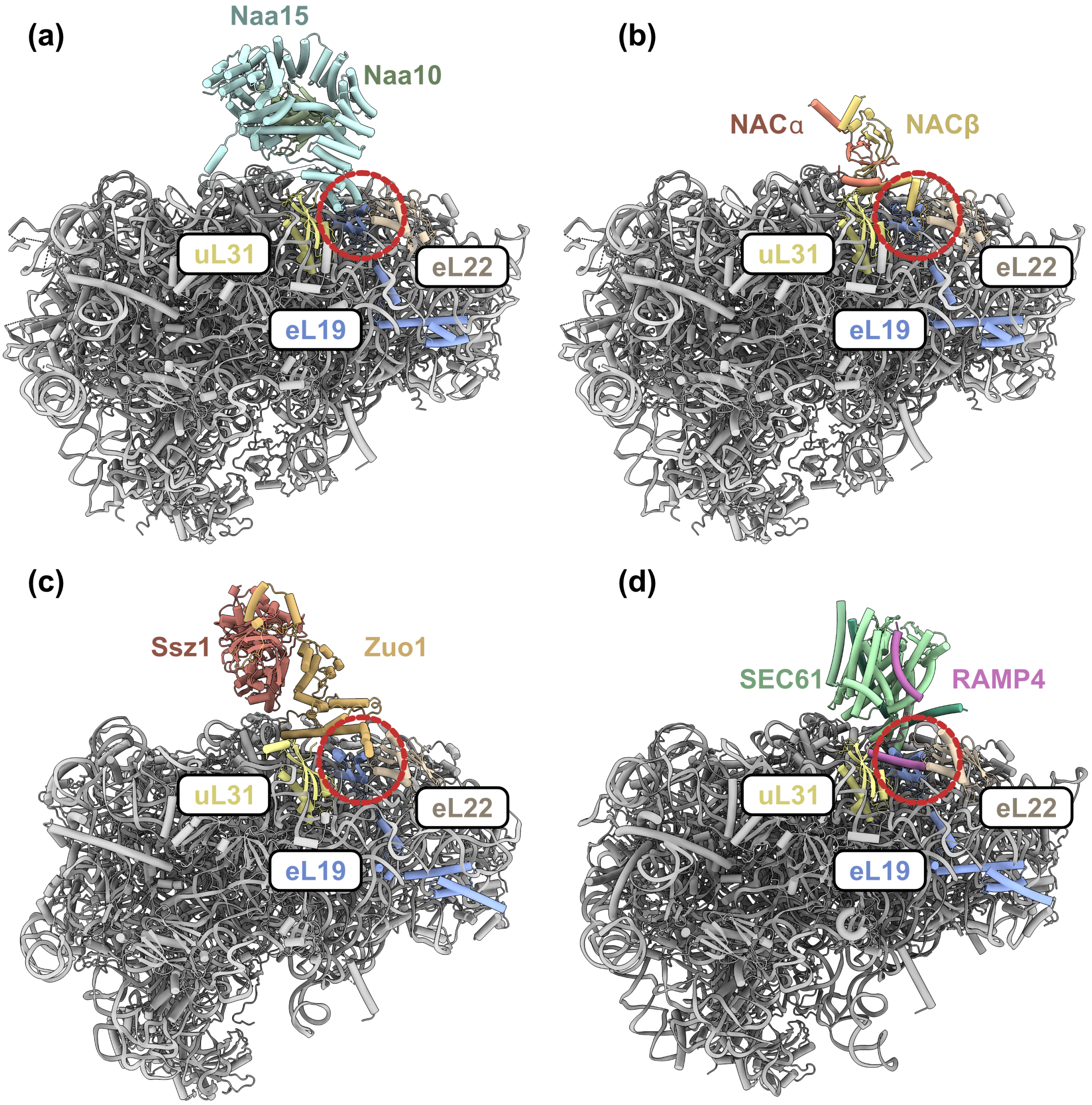

Supplementary Figure 16: The NatA proximal position competes with NACβ, Zuo1 and RAMP4. (a) Proximal NatA binds in the interface between uL31, eL19 and eL22. This binding site (encircled in red) is also utilized by **(b)** the helical NACβ anchor (PDB: 9FQ0 (ref: [1])), **(c)** The C-terminal part of the extended Zuotin homology domain helix of RAC (in state 2) (PDB: 7Z3O (ref: [2])), as well as **(d)** the hook-shaped ribosome anchor of RAMP4 in the ribosome translocon complex (PDB: 8RJB[3]).

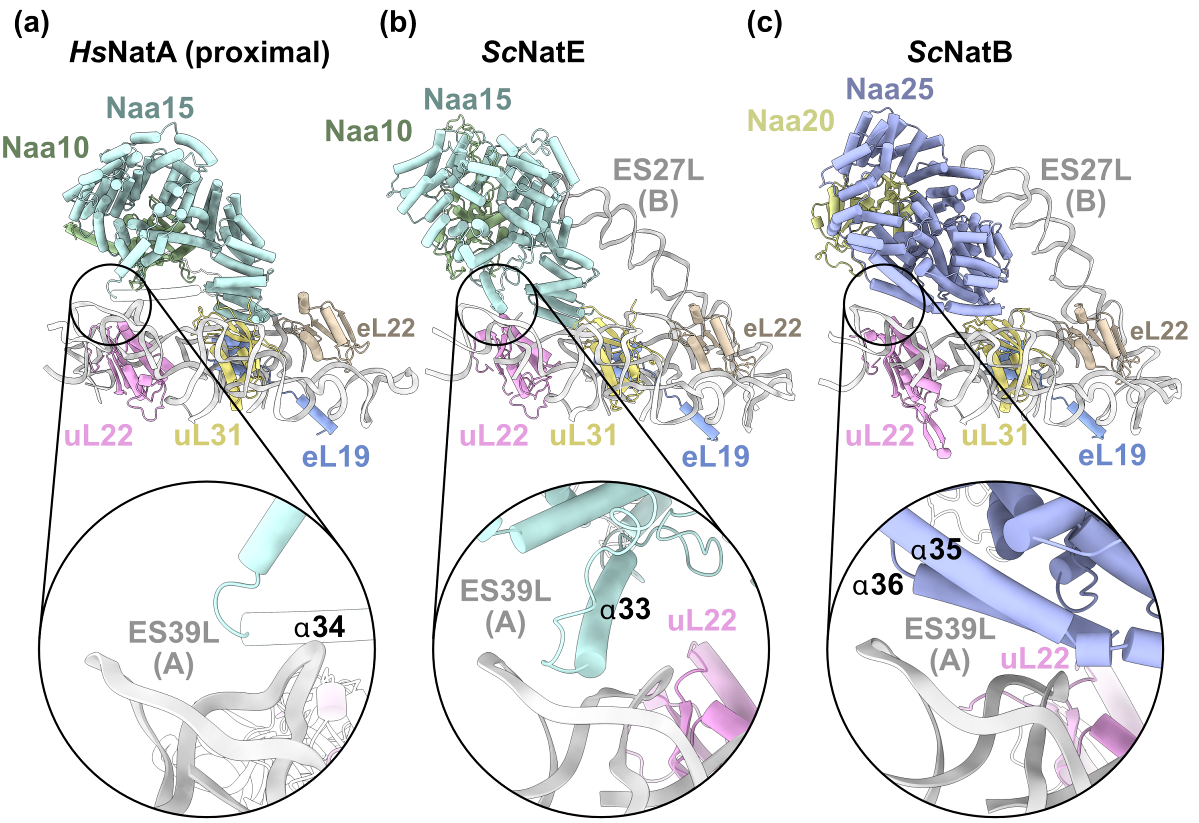

Supplementary Figure 17: ES39L is an important contact point for NatA, NatE and NatB. (a) In the proximal position, *Hs*NatA contacts an exposed knick-loop of ES39L(A). While the linker trajectory is well visible in the cryo-EM map, the α34 helix is not resolved (shown as transparent cylinder). **(b)** *Sc*NatA (pdb: 6HD7 (ref. [4])) has a different binding site than proximal *Hs*NatA but also utilized ES39L(A) as a contact point via α33 (corresponds to human α34). *Sc*Naa50 is not shown in the figure. **(c)** *Sc*NatB is positioned on top of ES39L(A), with two α-helices (α35 and α36) placed in parallel to the rRNA (PDB: 8BIP[5]).

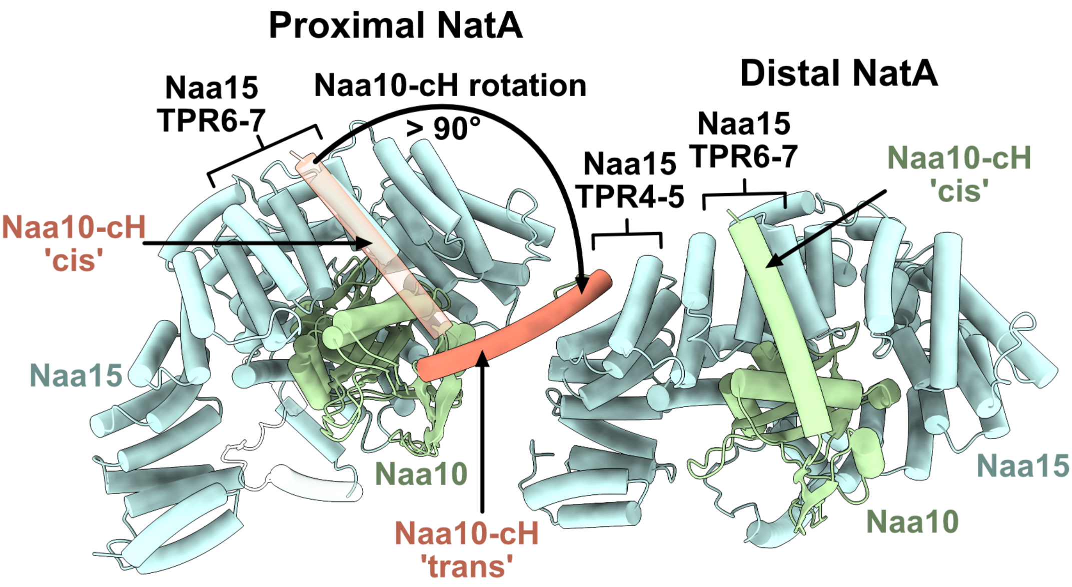

Supplementary Figure 18: The Naa10-cH is an adaptive element that can undergo a >90° rotation when adopting the proximal site. In the distal site, the Naa10-cH is arranged in 'cis' and positioned in parallel to Naa15 TPR6-7. When adopting the proximal site in the NatA-NatA-80S complex, the Naa10-cH undergoes a >90° rotation to enable the interaction with TPR4-5 of distal NatA in 'trans'.

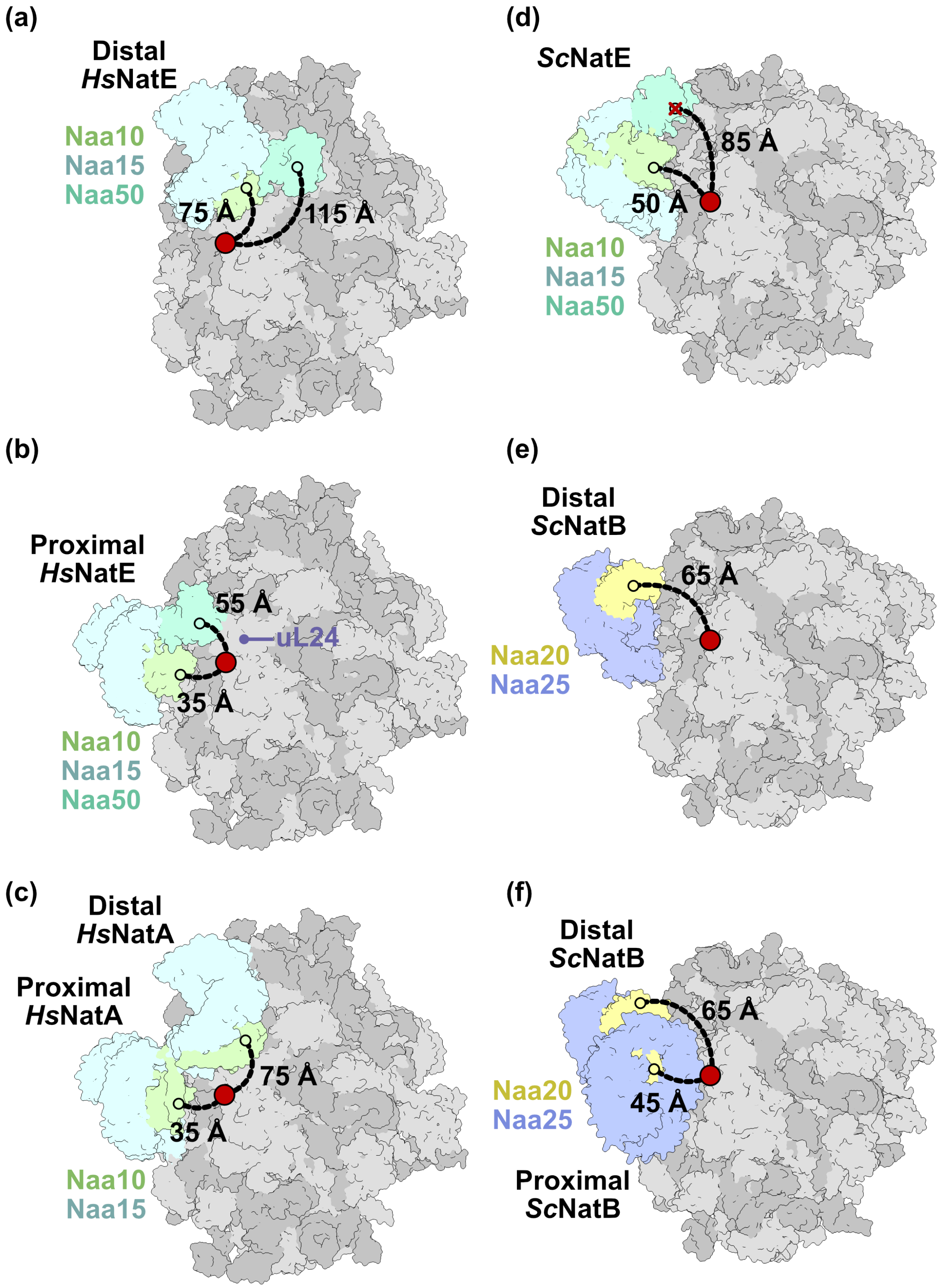

Figure description is located on the next page

Supplementary Figure 19: Coordination of Naa10, Naa20 and Naa50 from different binding sites. (a) Figure shows the structure *Hs*NatE when Naa50 is coordinated from the *Hs*NatA distal site (PDB: 9F1B[6]). **(b)** Hypothetical model showing the position of *Hs*NatE at the ribosome if Naa50 is coordinated from the proximal site. **(c)** The structure of *Hs*NatA in the distal and proximal site (this study) is shown, as well as **(d)** the structure of *Sc*NatE[4]. Of note, Naa50 is catalytically inactive in yeast (indicated by red cross). In addition, the **(e)** *Sc*NatB-80S structure (NatB-1 state) (PDB: 8BIP[5]) and the **(f)** ternary *Sc*NatB-*Sc*NatB-80S (NatB-1 and NatB-2 states) structures (PDB: 8BJQ[5]) are shown and labelled as distal and proximal *Sc*NatB, respectively. The position of the PTE is marked by a red circle. Distances to the active sites of Naa10, Naa20 and Naa50 are indicated by a dashed line. Of note, panel (b) shows a hypothetical model that was generated by superimposing the structure of *Hs*NatE (PDB: 6PPL[7]) onto the structure of proximal NatA. Distances were measured from the last 28S rRNA PTE constriction at G2416 (ref. [8])).

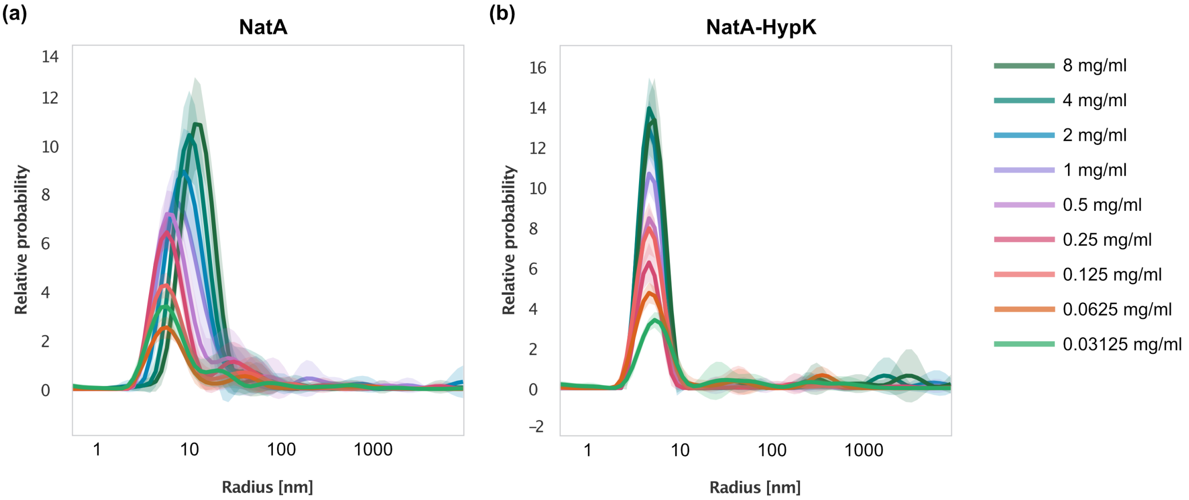

Supplementary Figure 20: DLS measurements reveal a concentration dependent dimerization of NatA. (a) At the highest measured concentration of 8 mg/ml the average hydrodynamic radius of NatA is 11.45 nm, suggesting that NatA has dimerized. Upon dilution, the average radius is shifted to smaller values, until reaching the lowest value of 4.79 nm at a concentration of 0.031 mg/ml, corresponding to the expected size of a single heterodimer of NatA. **(b)** In the presence of HypK, the average hydrodynamic radius remains steady in a range between 5-6 nm, independent of the NatA concentration, indicating that HypK prevents the NatA dimerization. Source data are provided as a source data file.

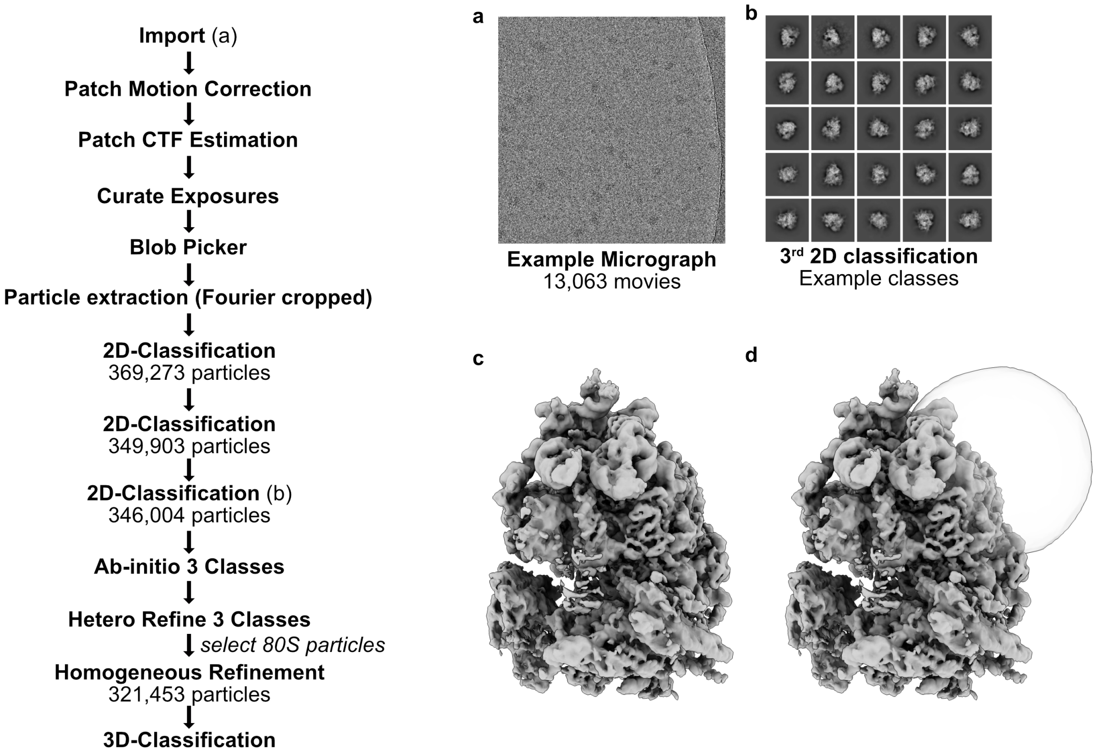

Supplementary Figure 21: Cryo-EM data processing for NatA-HypK and the 80S ribosome. To test whether NatA can still bind at the ribosomal PTE when complexed by HypK, a cryo-EM dataset was acquired. **(a)** 13,063 movies were collected and pre-processed in CryoSPARC. **(b)** Extracted particles were subjected to three rounds of 2D classification. Ab-inition reconstruction into three classes yielded seed volumes for Heterogeneous Refinement. **(c)** 80S particles were subjected to Homogenous Refinement, but the resulting map did not show any additional signal around the PTE, indicating that NatA-HypK did not bind in the distal or proximal site. **(d)** A spherical mask was generated to encompass both NatA binding site and used for focused 3D classification. None of the resulting maps revealed any signal for NatA-HypK at the ribosome.

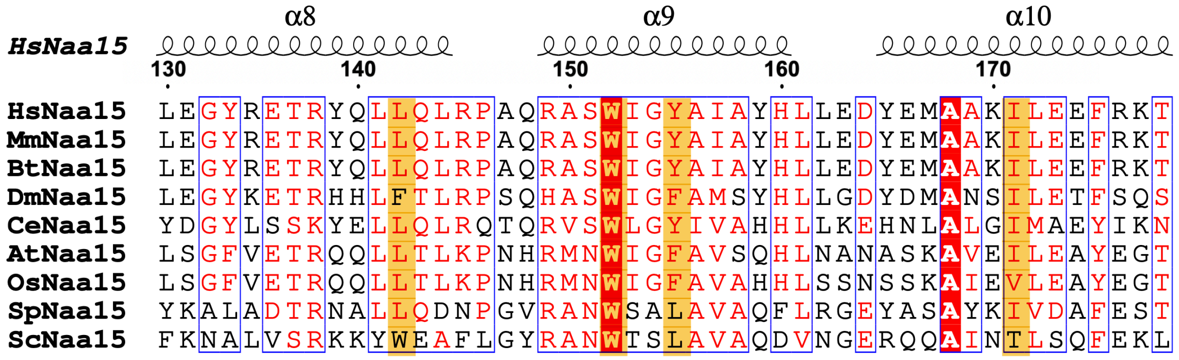

Supplementary Figure 22: Multiple sequence alignment of Naa15 α8-10 of TPR4-5. Alignment composes of Naa15 from *Homo sapiens* (Hs), *Mus musculus* (Mm), *Bos taurus* (Bt), *Drosophila melanogaster* (Dm), *Caenorhabditis elegans* (Ce), *Arabidopsis thaliana* (At), *Oryza sativa* (Os), *Schizosaccharomyces pombe* (Sp) and *Saccharomyces cerevisiae* (Sc). Residues that contribute to the exposed lipophilic patch in between TPR4-5 are highlighted in yellow. The hydrophobic character in these positions is conserved between these organisms. A tryptophan residue in the center of the lipophilic patch is especially well conserved.

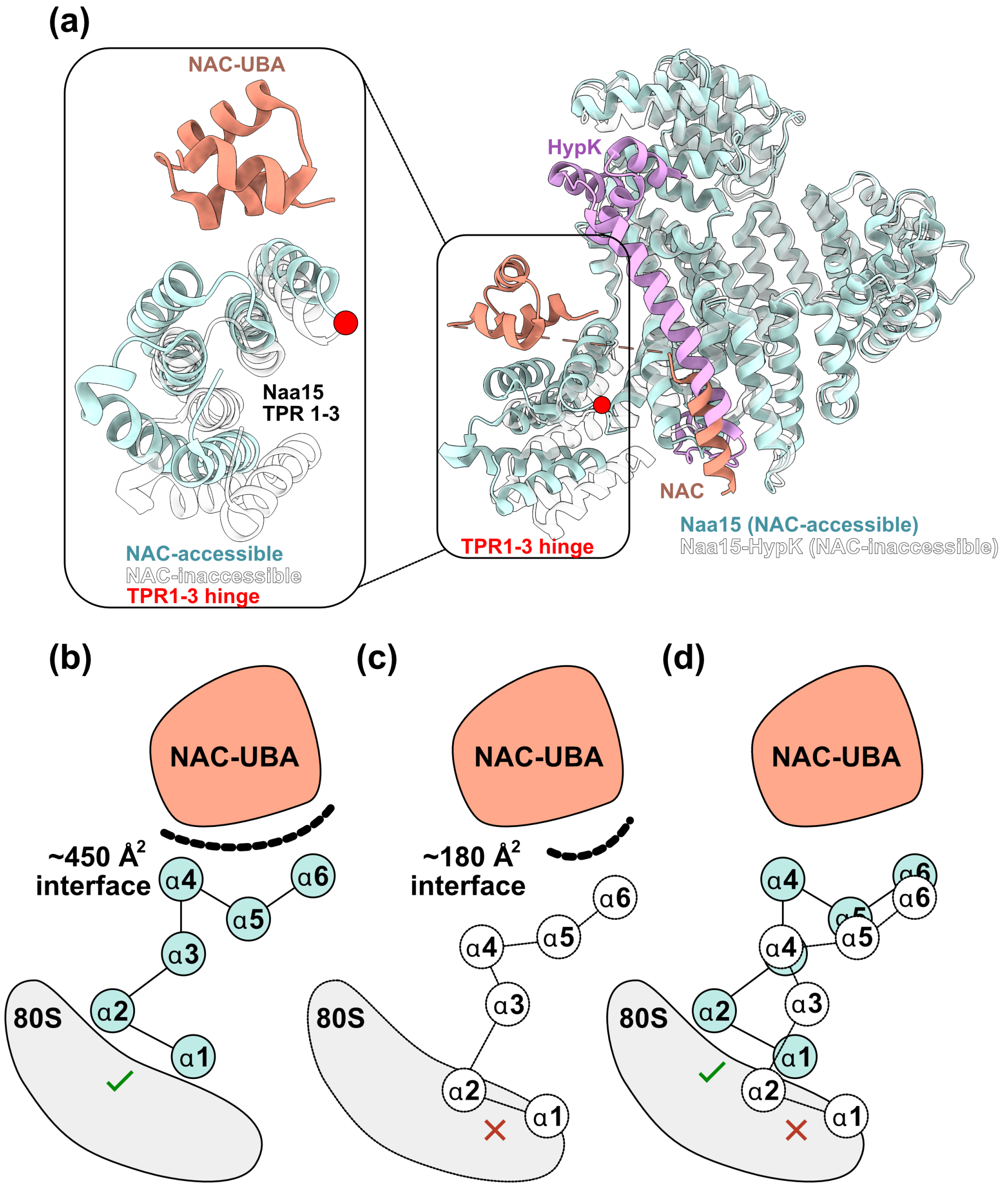

Supplementary Figure 23: HypK- or ribosome binding shapes the NAC-UBA binding site on Naa15. (a) Structures of NatA-HypK (PDB: 6C95)[9] (Naa15 and HypK are shown in transparent and purple, respectively) and NatA-NAC (PDB: 9F1D)[6] (Naa15 and NACα are shown in blue and red, respectively) are superimposed on Naa15. Only the NACα-UBA domain and cH are shown. NACβ, Naa10 or the ribosome are not shown in the figure. TPR1-3 is an adaptable element that can rotate around a flexible hinge, positioned between Naa15 helices α6 and α7 (red dot). Binding to HypK tightens up Naa15 TPR1-3, making this binding site inaccessible for the NAC-UBA domain. In complex with the ribosome, Naa15 TPR1-3 opens up and becomes accessible for the NAC-UBA. **(b)** Binding of NatA to the ribosome allows the NAC-UBA domain to contact Naa15 helices α4-5, by forming a ~450 Å^2 interaction interface (PISA server[10]). **(c)** Binding to HypK prevents the NatA-80S interaction and rotates Naa15 TPR1-3 away. Without rearrangements in the NAC-UBA, this would reduce the Naa15-NAC-UBA interaction interface to ~180 Å^2 (PISA server[10]). **(d)** Comparison of (b) and (c).

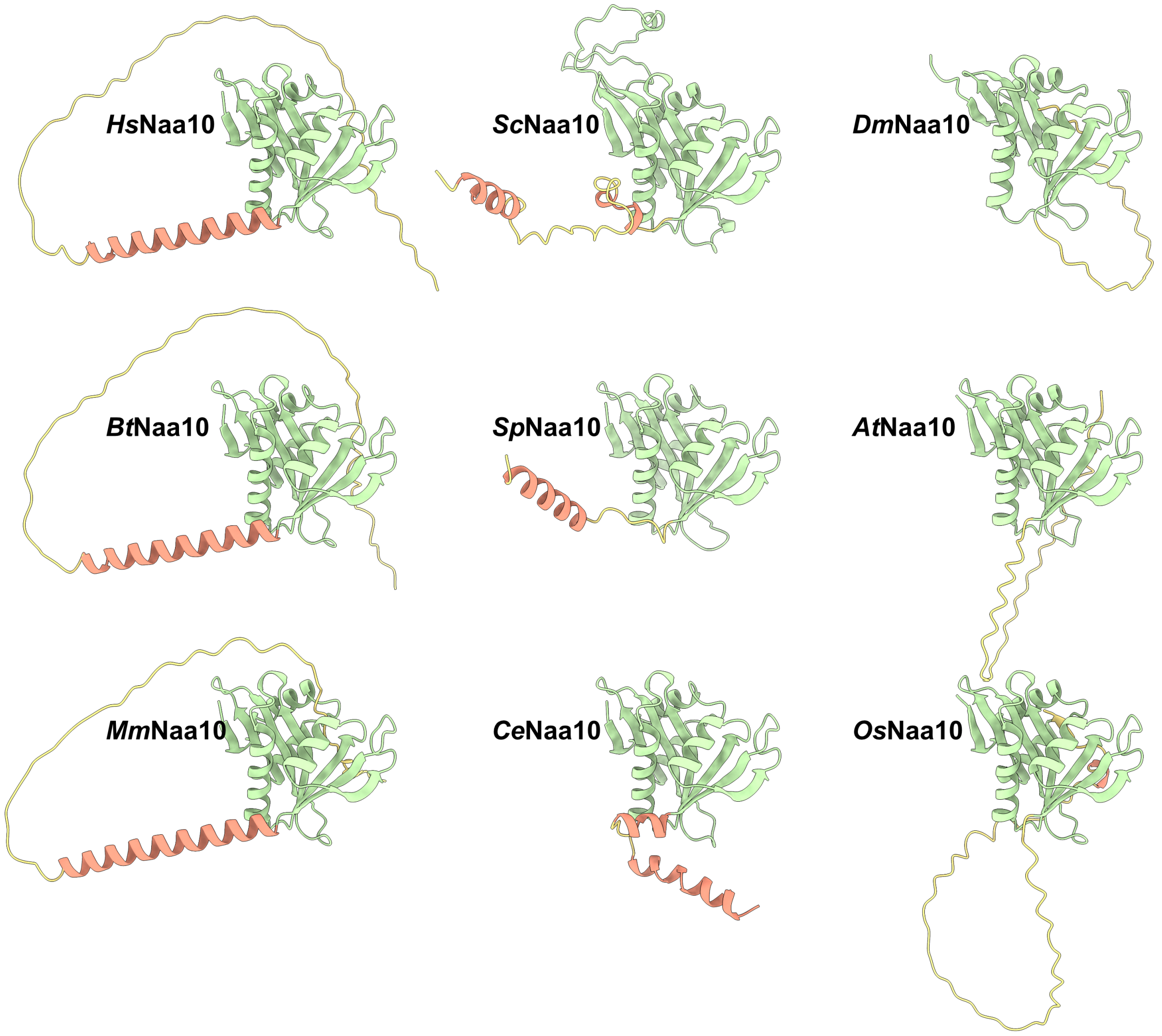

Supplementary Figure 24: Conservation of the Naa10 C-terminal extension across different organisms. Alphafold 2 predictions of full length Naa10 are shown for *Homo sapiens* Naa10 (*Hs*Naa10, UniProt: P41227), *Saccharomyces cerevisiae* Naa10 (*Sc*Naa10, UniProt: P07347), *Drosophila melanogaster* Naa10 (*Dm*Naa10, UniProt: Q9NHD5), *Bos taurus* Naa10 (*Bt*Naa10, UniProt: Q2KI14), *Schizosaccharomyces pombe* Naa10 (*Sp*Naa10, UniProt: Q9UTI3), *Arabidopsis thaliana* Naa10 (*At*Naa10, UniProt: Q9FKI4), *Mus musculus* Naa10 (*Mm*Naa10, UniProt: Q9QY36), *Caenorhabditis elegans* Naa10 (*Ce*Naa10, UniProt: O61219) and *Oryza sativa* Naa10 (*Os*Naa10, UniProt: A3AXS9). Regions corresponding to the Naa10-cH are highlighted in red. C-terminal segments predicted to be unstructured are coloured in yellow.

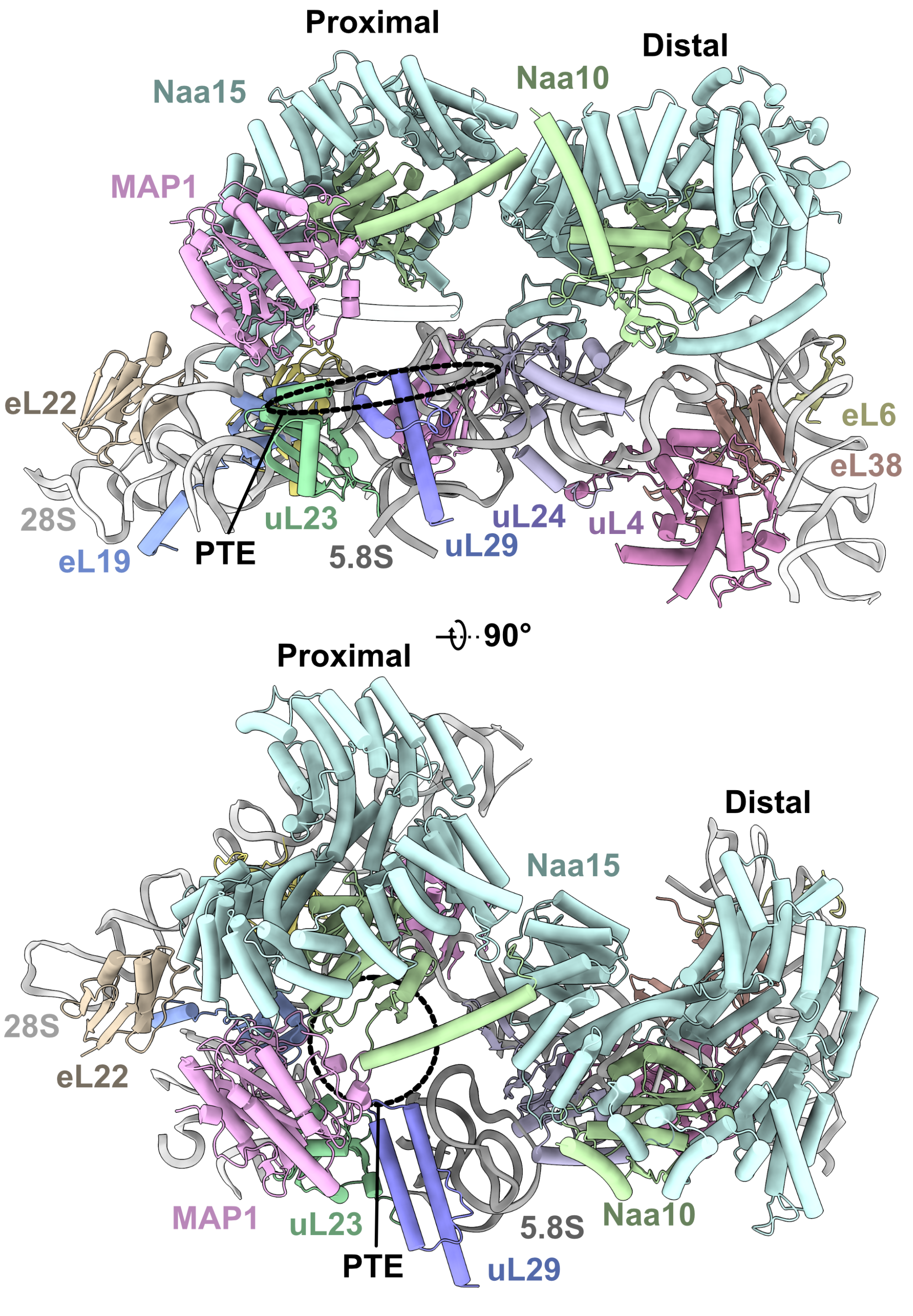

Supplementary Figure 25: Model of NME-NTA coordination between MAP1 and proximal NatA. The proximal and distal sites of NatA do not block the MAP1 binding site at the PTE. In this hypothetical multi-protein assembly, the active site of MAP1 and proximal Naa10 would be positioned directly on top of the PTE. This composite model was generated by superimposing the NatA-NatA-80S structure (this study), with the structure of MAP1 on the human ribosome (PDB: 9FQ0 (ref. [1])).

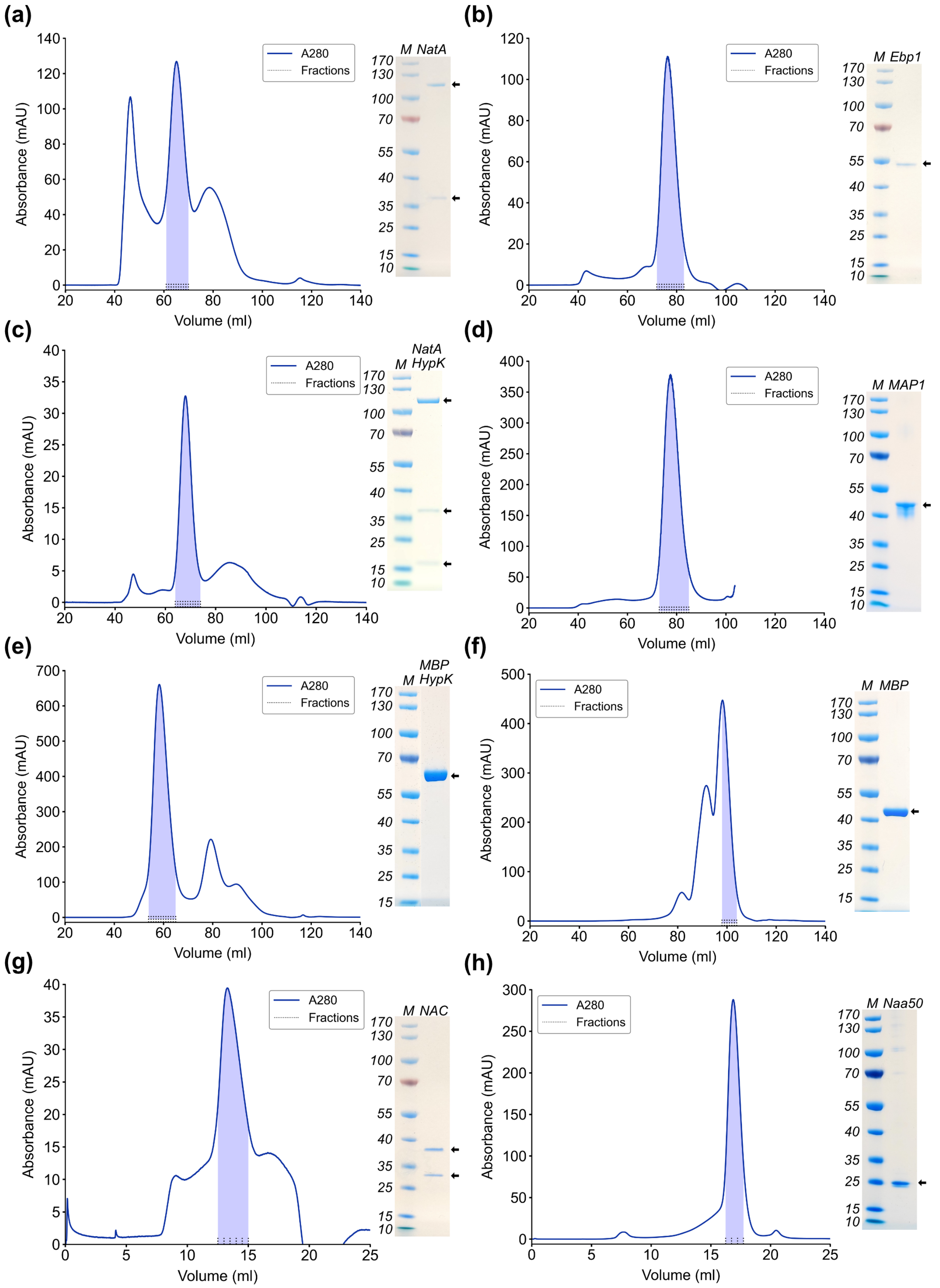

Figure description is located on the next page

Supplementary Figure 26: Protein purifications of ribosome associated factors. Preparative SEC runs are shown with selected fractions shaded in blue. Pooled fractions were analyzed by SDS-PAGE and coomassie staining. Bands corresponding to the protein of interest are indicated by an arrow. Purifications of **(a)** NatA[1], **(b)** Ebp1, **(c)** NatA-HypK, **(d)** MAP1[1], **(e)** MBP-HypK and **(f)** MBP were done on an S200 16-600 column (Cytiva). **(g)** NAC[1] and **(h)** Naa50 were purified with an S200 10/300 column (Cytiva).

Supplementary Table 1 | Cryo-EM data refinement statistics

**Model**	***Hs*NatA-*Hs*Ebp1-80S**	***Hs*NatA-*Hs*NatA-80S**
Data collection statistics		
Microscope	Glacios	Glacios
Camera	Falcon 3	Falcon 3
Voltage (kV)	200	200
Magnification	120,000	120,000
Total dose ($e^-/Å^2$)	51.65 Å/px, 55.07 Å/px	27.51 Å/px
Defocus range (μm)	-0.7 to -1,7	-0.7 to -1,7
Calibrated pixel size (Å)	1.223	1.223
Refinement statistics		
Refined particles	17,235	17,765
Resolution (Å)	4.61	3.91
Chains	14	18
Atoms	29428 (Hydrogens: 0)	43303 (Hydrogens: 0)
Residues	Protein: 2672 Nucleotide: 358	Protein: 3686 Nucleotide: 610
Water	0	0
Ligands	IHP: 1	IHP: 2 MG: 1
Bonds (RMSD)*		
Length (Å) (# > 4σ)	0.005 (0)	0.005 (0)
Angles (°) (# > 4 σ)	0.869 (30)	0.910 (41)
MolProbity score	2.31	2.15
Clash score	30.18	24.90
Ramachandran plot (%)		
Outliers	0.23	0.19
Allowed	4.76	3.72
Favored	95.01	96.08
Rama-Z (Ramachandran Plot, Z-score, RMSD)*		
whole (N = 951)	-0.51 (0.16)	-0.59 (0.13)
helix (N = 379)	0.07 (0.14)	-0.06 (0.11)
sheet (N = 107)	-0.22 (0.40)	-0.93 (0.31)
loop (N = 465)	-0.69 (0.18)	-0.53 (0.16)
Rotamer outliers (%)	0.00	0.00
Cβ outliers (%)	0.04	0.03
Peptide plane (%)		
Cis proline/general	0.0/0.0	0.0/0.0
Twisted proline/general	0.0/0.0	0.0/0.0
CaBLAM outliers (%)	1.98	1.58
ADP (B-factors)		
Iso/Aniso (#)	29428/0	43303/0
min/max/mean		
Protein	-0.00/1022.24/350.04	24.06/1028.85/229.13
Nucleotide	63.81/459.13/219.13	65.47/509.90/207.88
Ligand	817.97/817.97/817.97	119.39/229.85/215.25
Occupancy (%)		
Mean	1.00	1.00
occ = 1 (%)	100.00	100.00
0 < occ < 1 (%)	0.00	0.00
occ > 1 (%)	0.00	0.00
Model vs. Data (CC mask)	0.70	0.57
Resolution according to model vs. map FSC = 0.143 (masked) (Å)	4.7	4.2

* RMSD: root-mean-squared-deviation

Supplementary References

1 Klein, M., Wild, K. & Sinning, I. Multi-protein assemblies orchestrate co-translational enzymatic processing on the human ribosome. *bioRxiv*, 2024.2006.2014.599006 (2024). https://doi.org/10.1101/2024.06.14.599006

2 Kišonaitė, M. *et al.* Structural inventory of cotranslational protein folding by the eukaryotic RAC complex. *Nat Struct Mol Biol* **30**, 670-677 (2023). https://doi.org/10.1038/s41594-023-00973-1

3 Lewis, A. J. O., Zhong, F., Keenan, R. J. & Hegde, R. S. Structural analysis of the dynamic ribosome-translocon complex. *Elife* **13** (2024). https://doi.org/10.7554/eLife.95814

4 Knorr, A. G. *et al.* Ribosome-NatA architecture reveals that rRNA expansion segments coordinate N-terminal acetylation. *Nat Struct Mol Biol* **26**, 35-39 (2019). https://doi.org/10.1038/s41594-018-0165-y

5 Knorr, A. G. *et al.* The dynamic architecture of Map1- and NatB-ribosome complexes coordinates the sequential modifications of nascent polypeptide chains. *PLoS Biol* **21**, e3001995 (2023). https://doi.org/10.1371/journal.pbio.3001995

6 Lentzsch, A. M. *et al.* NAC guides a ribosomal multienzyme complex for nascent protein processing. *Nature* (2024). https://doi.org/10.1038/s41586-024-07846-7

7 Deng, S., McTiernan, N., Wei, X., Arnesen, T. & Marmorstein, R. Molecular basis for N-terminal acetylation by human NatE and its modulation by HYPK. *Nat Commun.* **11**, 818 (2020). https://doi.org/10.1038/s41467-020-14584-7

8 Kišonaitė, M., Wild, K., Lapouge, K., Ruppert, T. & Sinning, I. High-resolution structures of a thermophilic eukaryotic 80S ribosome reveal atomistic details of translocation. *Nat Commun* **13**, 476 (2022). https://doi.org/10.1038/s41467-022-27967-9

9 Gottlieb, L. & Marmorstein, R. Structure of Human NatA and Its Regulation by the Huntingtin Interacting Protein HYPK. *Structure* **26**, 925-935 (2018). https://doi.org/10.1016/j.str.2018.04.003

10 Krissinel, E. & Henrick, K. Inference of macromolecular assemblies from crystalline state. *J Mol Biol* **372**, 774-797 (2007). https://doi.org/10.1016/j.jmb.2007.05.022
