# Supplementary material for: NatA engages in multi-factor complexes at the ribosomal polypeptide tunnel exit
Source: Nat Commun. 2026 Jan 23;17:884. doi: 10.1038/s41467-026-68787-5 (PMC12830823; doi:10.1038/s41467-026-68787-5)
Supplement: Supplementary file 3 — Transparent Peer Review file [file 41467_2026_68787_MOESM3_ESM.pdf]

nature portfolio

Peer Review File

NatA engages in multi-factor complexes at the ribosomal polypeptide tunnel exit.

Corresponding Author: Professor Irmgard Sinning

This file contains all reviewer reports in order by version, followed by all author rebuttals in order by version.

Version 0:

Reviewer comments:

Reviewer #1

(Remarks to the Author)
In the current manuscript, Klein et al. perform several in vitro experiments to study the interaction of HsNatA with 80S ribosomes and various RAFs as well as the regulatory protein HypK, both independent and in the context of the ribosome. The majority of the work presented relies on the structural analysis of complexes reconstituted in vitro and their comparison with previously published work, as well as in vitro experiments assaying binding kinetics. The principal advance of the study is represented by the observation that Ebp1-NatA and NatA-NatA complexes can exist on the ribosome in vitro. Moreover, the authors identify a shared site on the surface of Naa15 contacted by a conserved binding helix of Ebp1, Naa10, and Nacα similar to that identified for HypK.
In cryo-EM studies the authors show the newly described assembly of the two NatA complexes co-existing on 80S ribosomes and discuss the potential physiological relevance of this finding.
Overall, it is novel and interesting to see that NatA can in principle bind the ribosome in the presence of Ebp1 and also together with a second copy of NatA, in each case engaging the second protein via a contact helix binding the same site on the Naa15 surface. However, the study suffers from a lack of in vivo evidence supporting the idea of a physiological relevance of these in vitro findings. Further, based on the presented data, the authors' conclusion that NatA may serve as a central coordinator at the ribosome, is rather speculative. In conclusion, the manuscript presents interesting new insights on the interplay of the ribosome-interacting factors NatA, Ebp1 and NAC which would be of interest to publish in Nature Comms. However, before publication it would be highly desirable to probe the significance of the observed interactions by mutational/biochemical experiments and, more importantly, to provide better evidence that the suggested interactions and functions actually occur in vivo.

Major comments:
In vivo IP
Page 11,15: The authors conclude that Ebp1 interacts with Naa15 in vivo based on co-IP experiments. However, Ebp1 is enriched only very mildly and with low significance in the results obtained with Naa15. Moreover, in their co-IP with Naa10, Ebp1 is not enriched at all. If in vivo Ebp1 indeed forms a complex with NatA, which consists of Naa15 and Naa10, one would expect clear enrichment in both cases. These data can thus not be interpreted in support of the author's hypothesis of a physiological role of the NatA-Ebp1 interaction on the ribosome.
Further, there is the question as to whether these results can indicate the joint presence of NatA and Ebp1 on the same ribosome at all, or rather the presence on different ribosomes in the context of a polysome. Here, it would be necessary to perform the co-IP experiments after a mild nuclease treatment to make sure that individual ribosomes and not also polysomes are purified, in which NatA and Ebp1 may interact individually with different ribosomes.

Fig 1 effect size
Figure 1c/d. Page 8: The subtitle of Fig1c/d is misleading as it implies a similar effect of Naa50 and HypK, while the measured effect differs greatly. In contrast to HypK, Naa50 shows barely any effect. Similarly, the authors state in line 200-202 that "Naa50 or HypK revealed a decrease in NatA binding to NAC" without taking into account the dramatic difference in magnitude. This should be pointed out in the text in a clear way.

Proximal vs Distal NatA, Function
Line 287: The authors compare the positions of proximal and distal NatA and the gap emerging subtrates would need to bridge from the PTE to Naa10. In this context, a functional experiment such as an acetylation assay would be interesting.

The authors describe the sorting scheme as first removing all particles that do not contain distal NatA but don't comment on these particles further. Did the dataset contain any particles exhibiting density for just the proximal NatA?
The speculation on the positioning of Naa50 in a hypothetical HsNatE-80S complex takes up a lot of space while not being based on any experimental data. This should be shortened considerably and discussed after the findings on the interaction in trans between proximal and distal NatA.
The authors argue that the presence of Naa16 in co-IP experiments with Naa15 indicates the existence of a NatA-NatA complex in vivo. However, also here, nuclease treatment of the samples before IP would be required at minimum to rule out a co-purification via polysomes.
Considering that the dimerization of NatA in DLS can be measured only at very high concentrations (mg/ml range), this should be mentioned in the text (line 362).

Early Discussion
Beginning at line 367, no new findings are introduced. Results are merely compared and illustrated. The following paragraphs should ideally be restructured and shortened to better fit the nature of a Results section, or be moved to Discussion.

Abundancy and "central coordination"
The authors suggest a role for (distal) NatA as a central coordinator of RAFs in vivo. However, in light of other studies (by the groups of Ban and others) and the rather low abundance of NatA compared to NAC in human cells, it is unclear to what degree NatA is actually capable of fulfilling this role. To this end, further results solidifying the involvement in the postulated complexes in vivo would be helpful.

Figure 4 representation of contact helix (cH) binding surface
In Fig 4c, the authors show a representation of the cH of different factors binding Naa15. Here, the representation of the latter should be reconsidered: Labels for residues 130 and 177 are in the exact same position, despite the surface representation obviously being viewed from different angles. This also makes it more difficult to judge the actual similarity of the binding modes. I suggest re-creating the figure 4c with Naa15 in the same orientation for all ligands.

More general, it is one of the key conclusions of the manuscript that the contact helices (cHs) of different RAFs (including NAC, Ebp1 and NatA itself) can interact with the same surface patch of NatA, which is then suggested to be in the position to act as a central coordinator on the ribosome. While the authors themselves do not provide any clear biochemical/genetic evidence that this suggested function exists in vivo, they should acknowledge/discuss previous data regarding the NatA-NAC interaction, showing that rather the UBA domain of NAC makes an essential contribution to NatA binding whereas mutations in the cH have comparably smaller effects.

Discussion
NAC coordinates co-translational enzymatic activity
Overall, the discussion is overly long and repetitive. It would benefit from inclusion of some of the information (e.g. comparing the effect size of Naa50 and HypK on NatA-NAC binding) in the Results and a removal of much of the redundant statements discussed in other places.
L427: Lentzsch et al recently showed N-acetylation in dependance on the length of the nascent chain in reticulocyte lysate. This should at least be cited here.
L463-468: These lines can be omitted or should be moved to the introduction.
Fig5a and b) The choice of star-shaped arrangement of RAFs does not add to the readability of the figure and should be replaced with e.g. a table or matrix.
L535: Why not reconstituting this complex (NatA-NatA-MAP1-80S)? The factors should all be available.
L541: This is redundant information that has been discussed multiple times so far in the paper
L553: Here, the same information is restated in different ways at least three times.
L572: The discussion of the regulation involving HypK appears to be too long, especially since it is based on very limited data

Minor comments:
Figure 1a: The choice of a striped bar for the non-existent binding of NatA-HypK to NAC is misleading and should be corrected. If no binding could be measured, the measurement should be shown as zero.
Figure 1b/d: A different color for the currently gray curves would improve contrast and readability.
Figure 1 Legend: "demolish" -> "abolish"
Figure 2 Legend: "fixate"
Figure 2 Legend: For (b) and (d) it should be clarified that these are partial models based on the experimental data, which omit NatA (b) and Ebp1 (d).
Figure 2d: For consistency, the arrow should point towards the "new" (teal) conformation observed in the Ebp1-NatA complex.
L 297: "touches down"?
L269: Omit "down" just write "towards the PTE"
L285: Add citation to previous work
Fig 3c: Here, too, the authors show a model of proximal NatA binding to the ribosome which is derived from their data on the ternary NatA-NatA-80S complex by omission of the distal NatA. This should be noted in the legend.
Supp Fig 15: Labels "remove classes without…." Should both be one level higher.
Fig 3c: The cH of Naa10 should be colored red as in (a) and (b) for consistency.

Reviewer #2

(Remarks to the Author)
Recommendation: publish with minor revision

In this manuscript, Klein et al. demonstrate that NatA serves as a central coordinator of co-translational protein maturation by dynamically assembling with multiple ribosome-associated factors (RAFs) at the polypeptide tunnel exit. Biochemical assays—including SEC-MALS and biolayer interferometry (BLI)—reveal that NAC can form ribosome-independent complexes with MAP1, NatA, and NatE. In particular, BLI "order-of-addition" experiments confirm that NAC mediates the interaction between NatA and MAP1. The authors further show that while Naa50 modestly impairs NatA's binding to NAC, HypK completely blocks this interaction. This is in particular surprising because a recent NatA-HypK-NAC-80S cryo-EM structure showed that NAC can bind to NatA in presence of HypK at the ribosome, but a possible structural explanation is discussed. Cryo-electron microscopy analysis uncovers that NatA can form a ternary complex with Ebp1 on the ribosome. In this assembly, Ebp1 binding remodels rRNA elements and competes with factors such as NACα and HypK for a conserved hydrophobic site on Naa15. Mass spectrometry experiments confirm that endogenous Naa15 interacts with Naa10, NACα, HypK, and Ebp1. Moreover, reanalysis of a NatA–80S dataset reveals a second, proximal binding site at the ribosome that positions the catalytic subunit Naa10 closer to emerging nascent chains, suggesting a mechanism for rapid substrate acetylation. Notably, proximal Naa10 contacts distal Naa15 at a conserved surface—centered on a critical tryptophan residue—similar to the binding sites used by NAC, MAP1, and Ebp1, indicating competitive interactions at the tunnel exit. Together, these findings position NatA as a versatile interaction hub that orchestrates multiple co-translational processing events critical for proteostasis.

This manuscript is well written and provides significant mechanistic insights into the co-translational regulation of protein maturation—a fundamental process in eukaryotic biology with broad implications for proteostasis and disease. By integrating high-resolution structural data with rigorous biochemical and biophysical analyses, the study not only expands our understanding of the multifaceted roles of NatA but also unveils novel mechanisms at the ribosomal tunnel exit. The discovery of a second, proximal binding site for NatA that enhances substrate accessibility is particularly innovative and could reshape how we view the spatial organization of co-translational modifications. Given its broad relevance to cell biology, structural biology, and biochemistry, this work is suitable for publication in Nature Communications.

Weakness of the manuscript:
The major weakness is that the study relies on in vitro biochemical assays and structural data and cellular experiments would strengthen the physiological relevance of the observed assemblies. While the discovery of distal and proximal binding sites for NatA is intriguing, the manuscript does not fully clarify how these sites differentially contribute to co-translational acetylation and overall proteostasis in cells.
The competitive binding observed among factors like NAC, HypK, and Ebp1 is well demonstrated in vitro. However, the mechanisms that modulate this competition in a cellular context remain somewhat speculative.
Furthermore, some flexible regions or extensions in the cryo-EM reconstructions are less well resolved, which may leave uncertainties regarding the complete interaction interfaces.
Since these questions open up further exciting directions of investigations and cannot be addressed in short periods of time, these points should not detract from the overall high quality of the work. Many groups working on the topic would as well benefit from these data, therefore the manuscript should be published as it is as an in vitro structural work.
Suggestions:
N-terminal acetylation is predominantly a co-translational modification. Might the assembly of NatA complexes in vitro in the absence of ribosomes reflect potential post-translational Nt-processing events like Nt-acetylation? Would immunopreciptiations from Naa15 or Naa10 from ribosomal and cytoplasmic fractionations show the same RAF interactions partners?

Minor Point
Fig.1: It would be beneficial to add the single measured points or error barswhere appropriate.

Reviewer #3

(Remarks to the Author)
In this manuscript, the authors investigate NAC interactions with NATs and MAPs in vitro, demonstrating that NatA can form high-affinity complexes with other ribosome-associated factors in the absence of ribosomes. Additionally, they resolve the structures of EBP1 and NatA in complex with the ribosome and revisit a previous dataset to identify a NatA dimer interacting at distinct positions near the tunnel exit.
While the study identifies interesting interactions, ribosome docking positions, and tertiary and quaternary complexes, the biological relevance of these findings remains unclear. The proposed functional roles of these complexes are largely speculative, and it is uncertain whether they exist in vivo under physiological conditions. Moreover, while the structural data provide valuable insights, they do not seem sufficient to support the proposed coordination mechanism. Strengthening the connection between these structures and a well-defined biological function would significantly improve the manuscript.
Major comments:
1) While NAC can form a ribosome-independent complex with MAP1 and NatA in vitro, the biological relevance of this interaction remains unclear. It has been proposed that NAC facilitates the release of HypK-mediated inhibition of NatA on the ribosome, thereby localizing NatA activity at the ribosomal tunnel exit. However, the formation of a ribosome-

independent complex appears to contradict this model; a ribosome-independent NAC-NatA complex may activate NatA, disrupting such NAC-mediated localization system and being detrimental for the cell.
In agreement with the potential danger of NatA-NAC ribosome-independent complexes, the authors report that HypK prevents the NAC-NatA interaction off the ribosome. This suggests that HypK may actively prevent off-the-ribosome NatA interactions with NAC in the cell. Could this mechanism explain the observed data? Addressing this question would help clarify the functional implications of the reported interactions.
2) The authors use mass spectrometry (Co-IP-MS) to assess the biological relevance of the observed interactions. However, cellular lysates were not treated with RNases prior to immunoprecipitation, meaning that polysomes remained intact. As a result, immunoprecipitating a ribosome-associated factor is likely to recover entire polysomes, leading to the co-purification of ribosome-associated factors that may not coexist on the same ribosome but are instead present within the same polysome. To support their claims, the authors must demonstrate that their immunoprecipitation is specific to individual ribosomes rather than polysomes. This limitation significantly weakens the conclusions drawn from these experiments and, unfortunately, leaves the existence of these complexes in vivo unproven.
3) Along these lines, can the authors comment on why EBP1 is only significantly enriched in the immunoprecipitate of NAA15 but not of NAA10 (Supp. Fig. 13)? If EBP1 and NatA were in complex on the same ribosome, one would expect that immunoprecipitating NAA10 would also lead to EBP1 enrichment. Overall, EBP1 enrichment appears relatively low compared to other ribosome-associated factors. Together with the methodological limitations discussed earlier, these observations further weaken the Co-IP-MS data as support for the existence of the proposed complex in cells.
4) His-tags are known to enhance the affinity of proteins for ribosomes, as previously reported (Supplemental Fig. S6 in Pech et al., J Biol Chem, 2010). Can the authors exclude the possibility that this effect is influencing their conclusions regarding EBP1 affinity and the salt resistance of its ribosome interaction?
5) I am not convinced of the relevance of the described proximal site of NatA or whether this binding site—or a dimeric state—is significantly populated in vivo. First, the authors identified this site through reanalysis of their dataset, suggesting that it is occupied by only a minor fraction of ribosomes in their cryo-EM sample. Additionally, NatA is present at substoichiometric levels compared to EBP1 and NAC, and this proximal binding site would be mutually exclusive with these highly abundant factors. If the proximal site is already sparsely occupied in an in vitro reconstituted system, it seems unlikely that it could effectively compete with other ubiquitous ribosome-associated factors in vivo. Furthermore, the functionality of a NatA dimer remains unclear. While the authors propose multiple potential mechanisms, these remain speculative.
It is therefore possible that the observed dimeric state is an artifact of the in vitro reconstitution conditions used for cryo-EM. This concern is further compounded by the limitations in the Co-IP-MS data discussed earlier. Addressing these issues, either through additional biochemical validation or functional assays, would be necessary to support the proposed mechanisms. So far, the authors' data demonstrate that these factors are able to simultaneously bind a ribosome, this does not imply that they represent a functional state or a coordination mechanism.
6) The authors claim that NatA plays a coordinating role in multifactor complexes at the ribosomal tunnel exit, as reflected in the title of the manuscript. However, the presented data do not support this conclusion. The authors observe that EBP1 shares the same binding site that NAC uses to displace HypK-cH for NatA activation. This raises the possibility that EBP1, like NAC, could facilitate NatA activation at the ribosome. Given the ubiquitous presence of both NAC and EBP1 on ribosomes, these findings suggest that NatA activation does not depend on a specific ribosome-associated factor but may instead occur through a factor-independent mechanism. This interpretation seems plausible but does not contradict the notion that NatA acts as a coordinator of other factors.
7) The authors' interpretation hinges on the assumption that a pool of HypK-free NatA exists in the cell. While they acknowledge that this is not established, their own mass spectrometry data show that HypK co-purifies with NAA15 and NAA10 at similar levels. Although Co-IP-MS is not strictly quantitative, this observation suggests that most cellular NatA exists in complex with HypK. This would agree with the need of keeping NatA inactive until it associates with the ribosome. Can authors comment on this?
In summary, while the structural data presented in this study are valuable for understanding ribosome-associated multicomplex interactions, I find that they are overinterpreted to propose mechanisms that remain hypothetical. Due to technical limitations, particularly in the Co-IP-MS experiments, the biological relevance of the described interactions is not convincingly demonstrated.

Minor Errata:
Page 11: "HypK-contact helices (cH) do".
Page 26: "…MAP2-like pseudoenzyme".

Version 1:

Reviewer comments:

Reviewer #1

(Remarks to the Author)
Reviewer comments revised version
NCOMMs-24-85546

In the revised version of their manuscript Klein et al. addressed several of our concerns satisfactorily. Nevertheless, some of the old and new data are still inconclusive and partially not appropriately interpreted and represented in the main text.

In vivo IP:

1. It should be clearly stated in the figure legend (Suppl. Fig 13) and the main text, that the co-immunoprecipitation Mass Spectrometry experiments are done without RNase treatment, whereas the newly added subsequent Co-IP Western blotting experiments were done with (or control without) RNase treatment.

2. Line 252 states in the revised version:

"To rule out that the Co-IP was polysome dependent (NAA15 and Ebp1 bound to separate ribosomes), lysates were treated with RNaseI or T1. However, subsequent Co-IP Western blotting experiments still clearly show the NAA15-EBP1 interaction."

However, the data presented in the new Suppl Fig. 13 b) and c) are rather inconclusive and this statement does not acknowledge the fact that after treatment with both RNases much less NAA15 co-immunoprecipitated with EBP1 than in the non-treated control indicating that both proteins were at least with very substantial amounts located on different ribosomes. In the case of RNaseI treatment surprisingly much less NAA15, but much more Naa10 precipitates with EBP1 than in the untreated control. This imbalance may indicate that Naa10 indeed associates but perhaps together with NAA16 and not NAA15. Moreover, in the case of RNase T1 treatment hardly any NAA15 or NAA10 co-immunoprecipitated with EBP1 even though the lysates nicely show similar levels of expression for all three tagged components. This should be clearly described in the main text (not only in the supplement) and critically discussed.
In the V5 –IP /Western blot experiment presented in the new Suppl. Fig 13 c the authors address the question whether an interaction between two distinct endogenous NatA complexes (namely, NAA15/NAA10 and NAA16/NAA10) might occur in vivo inside HeLa cells.
Here again, NAA15-V5 with or without RNase treatment co-immunoprecipitated only a minor fraction of Naa16, whereas NAA10-V5 co-immunoprecipitated a 2- to 3-fold excess of NAA16 (over NAA10) even though the lysates nicely show similar levels of expression for all three components.
Taken together Suppl Fig 13 b) and c) could also imply that EBP1 rather interacts with a NAA16/NAA10, than with a NAA15/NAA10 complex in vivo.
These data require a better description in the main text than the highly simplified statements in the revised main text:
Line 254
However, subsequent Co-IP Western blotting experiments still clearly show the NAA15-EBP1 interaction."
and
Line 351
"However, subsequent Co-IP Western blotting experiments still clearly show NAA15-NAA16 interaction."

Proximal versus distal NatA function
Our former request was (5.):
"Considering that the dimerization of NatA in DLS can be measured only at very high concentrations (mg/ml range), this should be mentioned in the text."
Previous main text:
"In order to test whether NAA15 composed NatA can dimerize in vitro and independent of the ribosome, we measured dynamic light scattering (DLS) of increasing NatA concentrations. Indeed, we observe that dimerization also occurs in the absence of the ribosome (Supplementary Fig. 20)."
Revised main text:
"In order to test whether NAA15 composed NatA can dimerize in vitro and independent of the ribosome, we measured dynamic light scattering (DLS) of increasing NatA concentrations (0.03-8 mg/ml). Indeed, we observe that dimerization also occurs in the absence of the ribosome (Supplementary Fig. 20)."
This is a misleading statement, since up to a concentration as high as 0.5mg/ml an average hydrodynamic radius of 5-6 nm representative of a single NatA heterodimer is observed. These results should also be described in the main text in a more complete and accurate way.

Taken together, the authors made a considerable effort to address the main points, however, without getting clear and consistent results. Therefore, we are not entirely convinced that the findings observed in vitro are indeed of physiological significance in the cell. We thus feel that the authors must tone down the text with respect to the in vivo data. After being completer and more transparent about the in vivo data in the main text (as suggested above), the authors should clearly state latest in the discussion that it is not yet clear to what end their interesting in vitro findings represent physiological behavior. Nevertheless, even without a totally convincing in vivo support for their data, I find their findings interesting and solid enough as to warrant publication in Nature Comms as soon as the data are represented in a more cautious way.

Reviewer #2

(Remarks to the Author)
I have only a few minor remaining points:

In Figure 1, replicates were added for panel a, but not for panel c. Replicates, SD or at least clear indication of n should be included consistently.

In Figure 2d, the "Naa15 rotated" label is barely legible

In the main text (line 252), please reference Supplementary Fig. 13a and at line 255, Supplementary Fig. 13b and c.

There is no textual reference to Supplementary Fig. 12a and b, please check.

Overall, the manuscript is much improved and suitable for publication after addressing these small points.

Reviewer #3

(Remarks to the Author)
I thank the authors for their response, which addressed some of my initial concerns. I appreciate the effort to clarify points raised in the first round, and I continue to value the structural insights presented in this study. That said, a few key issues remain unresolved.

Critical points

1. Title of the manuscript and claim of NatA coordinating tunnel exit complexes
I remain unconvinced by the title and the claim that NatA coordinates multi-factor complexes at the ribosomal tunnel exit. Authors show that NatA can form multiple complexes with different factors at the ribosomal tunnel exit. Yet, evidence showing that NatA regulates or coordinates the formation and activity of these complexes is not provided.
On the contrary, the data suggest that NAC, and possibly EBP1, regulate NatA activity at the ribosome. For this reason, the title should be revised to more accurately reflect the findings.

2. Co-IP-MS experiments
First, from a technical perspective, my concern regarding the Co-IP-MS data remains (point 2 of my previous review). Although the authors attempted to validate the Co-IPs under RNase-treated conditions using WB, the Co-IP-MS itself must also be repeated with RNase-pretreated lysates to ensure the data reflect direct ribosomal interactions. Without this, the results represent proteins co-existing in polysomes rather than on individual ribosomes, which is misleading. This experiment must either be repeated using RNase-treated polysomes or removed from the manuscript.
Second, the new western blot evidence presented to support a direct NatA-EBP1 interaction on individual ribosomes (generated by RNase treatment) is not convincing. Instead, it shows a marked reduction in the co-immunoprecipitation of Naa10 and Naa15 upon RNase treatment, especially with RNase T1. The levels of co-immunoprecipitated Naa10 after RNase T1 treatment approach those of the control (supplementary Fig 13, lane 3) rather than the untreated condition. This raises concerns that the NatA - EBP1 interaction strongly depends on polysome integrity, making its in vivo co-existence on the same ribosome uncertain. While I acknowledge that RNase digestion itself can affect ribosome binding and that the NatA-EBP1 complex might be of low abundance, the authors should address this issue directly and temper their claims regarding in vivo relevance.

In summary, I strongly recommend that all Co-IP experiments performed without prior RNase treatment either be repeated under proper conditions or removed. Claims derived from these experiments must be aligned with the actual data.

Other comments

3. Proximal NatA binding site
It remains unclear whether the proximal NatA binding site exists in vivo and what its proposed function might be. So far, this conformation has only been observed in vitro under restricted conditions and in the absence of HypK. Since the authors show that NatA-HypK cannot self-promote ribosome binding, this pathway seems unlikely to relief HypK inhibition. The suggestion that free NatA may serve this role is possible but remains speculative. Providing evidence that such a mechanism is plausible would strengthen this interpretation. At present, any functional assignment to the NatA dimer is speculative and the claims should be toned down accordingly.

Overall assessment
Despite these reservations, I want to emphasize that the structural data are of high value to the community and making them available will be useful for the field. However, the lack of a clearly defined biological context or demonstrated function reduces the overall impact of the study.
I would support publication in Nature Communications provided that (i) the Co-IP-MS experiments are repeated with RNase-treated lysates (or else removed) and (ii) the manuscript title is adjusted to better reflect the actual findings.

Reviewer #4

(Remarks to the Author)
The authors have addressed the majority of concerns raised by the previous Reviewers. However, considering that the manuscript's novel findings center on the Ebp1-NatA-80S and NatA-NatA-80S complexes, the most critical issues (three reviews all had the same concerns) regarding the biological relevance of these two complexes remain unresolved. In the revision the authors provide additional evidence (via co-immunoprecipitation after RNase I/T treatment) for the existence of Ebp1-NatA and Naa15-Naa16 interactions in vivo, the claim that Ebp1-NatA-80S and NatA-NatA-80S complexes function in vivo lacks convincing experimental support.
Moreover, both two complexes are not organized in a manner that could achieve successive NME and NTA.
For Ebp1-NatA-80S: This complex lacks N-terminal methionine excision (NME) activity, raising questions about its functional relevance in sequential NME/NTA, though the authors proposed a place-holder function of Ebp1.

For NatA-NatA-80S: The positioning of the proximal NatA within this complex would sterically hinder the binding of both NAC-MAP1 and MAP2, preventing coordinated NME and NTA. The authors propose a potential "NatA-NatA-MAP1-80S" complex to overcome this, but crucially, they did not pursue structural validation as previously recommended by Reviewer #1. Furthermore, while functional assays probing acetylation activity were suggested, mechanistic details confirming how NatA-NatA achieves NTA are still lacking.
In summary, while progress has been made in addressing some previous concerns, this revised manuscript still lacks sufficient experimental evidence to substantiate the functionality of the two in vitro complexes.

Reviewer #5

(Remarks to the Author)
In this articles Klein and colleagues analyze the interplay of NatA with other ribosome-associated factors (RAFs) at the ribosome and apart from it.
N-terminal acetylation (NTA) is a major protein modification in eukaryotes, mainly carried out by the NatA complex after N-terminal methionine excision (NME). This study reveals that NatA not only acts at the ribosome but can also form ribosome-independent assemblies with several ribosome-associated factors (RAFs). At the ribosome, NatA can form ternary complexes with either the pseudoenzyme Ebp1 or another NatA copy, enabling flexible substrate coordination. The findings suggest that NatA serves as an interaction hub, organizing multiple factors to coordinate co-translational protein maturation.
The authors mainly use an in vitro approach, but during the revision added CoIP experiments to corroborate their findings also in cellulo.
I have specifically been asked to assess the comments of reviewer 1, who apparently was not available anymore for the reviewing process.
In my opinion, the following comments appear to be satisfactorily addressed in full – I have no further comments
• Fig 1 effect size
• Proximal vs Distal NatA, Function Issues 2, 3, 4 and 5
• Early Discussion – this should have never been a major issue
• Figure 4 representation of contact helix (cH) binding surface
• Discussion - NAC coordinates co-translational enzymatic activity
• Minor comments
In my opinion, the following comments appear to be partially addressed – see my further comments
• Proximal vs Distal NatA, Function Issue 1 – I agree with the authors and I am also not convinced that “an acetylation experiment would allow (us) to distinguish the activity of distal and proximal NatA”. I am fine with the response of the authors and I am convinced that the authors would also like to figure out the functional role of the distal NatA molecule.
• Abundancy and “central coordination” – I fully agree with the reviewer that this is a critical point, but it is also wishful thinking to answer these complex questions in vivo. I do agree with the response of the authors that the presented study provides suitable models that need to be tested in the future. The low abundance indeed makes it hard to associate such a central role, but this rather also asks for a quantification of RAFs in cells under different conditions. The study does show that eventually NatA does play a more central role than previously acknowledged and that NAC is not the only coordinating center. In my opinion, authors did work on the respective sections and down toned some of their statements.

Overall assessment – the authors provided several new insights into these complex regulatory layer at the ribosome that is central to a large fraction of our proteomes. Furthermore, the authors provide clear biochemical and structural evidence that supports their main claims. I do agree with reviewer 1 (and reviewer 3) that some parts of the study will still require additional validation in vivo. In my opinion, the authors addressed these issues by performing CoIP experiments at a satisfactorily level – however, I am aware that many more in vivo experiments are needed to clarify these RAF networks (at the ribosome and apart form it). This does leave a taste of uncertainty, but this is most often the case for studies that are mainly focusing on revealing the mechanistic reasons behind cellular machines.

For all of the above reasons, I would support publication of the work in Nature Communications. Not because, this study provides all the final answers, but because it points the field into directions of several important links between RAFs that need to be investigated in the future (under different cellular conditions). I think that most of the overstatements have been removed during the revision process – I did not find outrageous statements any more.

Open Access This Peer Review File is licensed under a Creative Commons Attribution 4.0 International License, which permits use, sharing, adaptation, distribution and reproduction in any medium or format, as long as you give appropriate credit to the original author(s) and the source, provide a link to the Creative Commons license, and indicate if changes were made.
In cases where reviewers are anonymous, credit should be given to 'Anonymous Referee' and the source.
The images or other third party material in this Peer Review File are included in the article's Creative Commons license, unless indicated otherwise in a credit line to the material. If material is not included in the article's Creative Commons license and your intended use is not permitted by statutory regulation or exceeds the permitted use, you will need to obtain permission directly from the copyright holder.
To view a copy of this license, visit https://creativecommons.org/licenses/by/4.0/

REVIEWER COMMENTS

NCOMMS-24-85546

Reviewer #1 (Remarks to the Author):

In the current manuscript, Klein et al. perform several in vitro experiments to study the interaction of HsNatA with 80S ribosomes and various RAFs as well as the regulatory protein HypK, both independent and in the context of the ribosome. The majority of the work presented relies on the structural analysis of complexes reconstituted in vitro and their comparison with previously published work, as well as in vitro experiments assaying binding kinetics. The principal advance of the study is represented by the observation that Ebp1-NatA and NatA-NatA complexes can exist on the ribosome in vitro. Moreover, the authors identify a shared site on the surface of Naa15 contacted by a conserved binding helix of Ebp1, Naa10, and Nacα similar to that identified for HypK.
In cryo-EM studies the authors show the newly described assembly of the two NatA complexes co-existing on 80S ribosomes and discuss the potential physiological relevance of this finding.
Overall, it is novel and interesting to see that NatA can in principle bind the ribosome in the presence of Ebp1 and also together with a second copy of NatA, in each case engaging the second protein via a contact helix binding the same site on the Naa15 surface. However, the study suffers from a lack of in vivo evidence supporting the idea of a physiological relevance of these in vitro findings. Further, based on the presented data, the authors' conclusion that NatA may serve as a central coordinator at the ribosome, is rather speculative. In conclusion, the manuscript presents interesting new insights on the interplay of the ribosome-interacting factors NatA, Ebp1 and NAC which would be of interest to publish in Nature Comms. However, before publication it would be highly desirable to probe the significance of the observed interactions by mutational/biochemical experiments and, more importantly, to provide better evidence that the suggested interactions and functions actually occur in vivo.

We are grateful for the detailed assessment of our work and finding it novel and interesting. Many of the reviewers' comments helped us strengthen and refine our manuscript. For this study, we focused our efforts on the question of "What can happen at the tunnel exit?". Our findings reveal novel interactions with intriguing implications on co-translational acetylation and overall RAF coordination. For the field of ribosome biology our findings are of high value as they allow for the design of targeted follow-up studies. We agree with the reviewer that our structures spark many new questions around the *in vivo* function of NatA-containing complexes. While we feel that addressing these questions all within this study would be beyond the scope of this manuscript, we made an effort to incorporate the reviewers' main suggestions including supporting Co-IP-WB experiments.

<u>Major comments:</u>

In vivo IP

Page 11,15: The authors conclude that Ebp1 interacts with Naa15 in vivo based on co-IP experiments. However, Ebp1 is enriched only very mildly and with low significance in the results obtained with Naa15. Moreover, in their co-IP with Naa10, Ebp1 is not enriched at all. If in vivo Ebp1 indeed forms a complex with NatA, which consists of Naa15 and Naa10, one would expect clear enrichment in both cases. These data can thus not be interpreted in support of the author's hypothesis of a physiological role of the NatA-Ebp1 interaction on the ribosome.
Further, there is the question as to whether these results can indicate the joint presence of NatA and Ebp1 on the same ribosome at all, or rather the presence on different ribosomes in the context of a polysome. Here, it would be necessary to perform the co-IP experiments after a mild nuclease treatment to make sure that individual ribosomes and not also polysomes are purified, in which NatA and Ebp1 may interact individually with different ribosomes.

We agree with the reviewer's comment, and have repeated the co-immunoprecipitation experiments using cell lysates pre-treated with RNase T1 and RNase I. These experiments confirmed that both NAA15 and NAA10 interact with EBP1 following RNase digestion, indicating that the interaction is not polysome-dependent. The absence of EBP1 enrichment in the co-IP MS experiment may be due to interference from the anti-NAA10 antibody disrupting the NAA10–EBP1 interaction. We therefore performed these new experiments with tagged proteins and used anti-tag antibodies for co-immunoprecipitation. (see Supplementary Figure 13).

Fig 1 effect size

Figure 1c/d. Page 8: The subtitle of Fig1c/d is misleading as it implies a similar effect of Naa50 and HypK, while the measured effect differs greatly. In contrast to HypK, Naa50 shows barely any effect. Similarly, the authors state in line 200-202 that "Naa50 or HypK revealed a decrease in NatA binding to NAC" without taking into account the dramatic difference in magnitude. This should be pointed out in the text in a clear way.

We thank the reviewer for pointing to this possible source of confusion. Indeed, Naa50 only marginally affects NatA binding to NAC, compared to HypK. We have rephrased the paragraph accordingly.

In the figure caption we made note of this by writing:

- *"While Naa50 only has a slight negative effect on NatA binding to NAC (left graph), HypK severely diminishes NAC binding to NatA."*
- *"The presence of Naa50 displaced a small amount of NatA from the biosensors, while HypK drastically diminished NAC binding, consistent with the observations made in (a) and (c)."*

Proximal vs Distal NatA, Function

1. Line 287: The authors compare the positions of proximal and distal NatA and the gap emerging substrates would need to bridge from the PTE to Naa10. In this context, a functional experiment such as an acetylation assay would be interesting.

   We agree that acetylation assays can be useful experiments to address the timing of enzyme function at the ribosome. Such a setup was used i.e. by Lentzsch and colleagues (2024) in context of distal NatA acetylation. However, it merely confirmed what was already visible in the cryo-EM structure. While applicable in this particular case, we are not convinced that an acetylation experiment would allow us to distinguish the activity of distal and proximal NatA. We believe that binding of two NatA copies at the ribosome might represent a specialized complex assembled under currently unknown conditions rather than the default state. Quantitative western blotting with an anti-Ac antibody may not allow us to clearly detect the function of proximal NatA, if this binding site happens to be under-represented under the given experimental conditions. Without high odds of resolving the timing of distal and proximal NatA, an acetylation assay may not provide the information needed to conclusively answer this question.

2. The authors describe the sorting scheme as first removing all particles that do not contain distal NatA but don't comment on these particles further. Did the dataset contain any particles exhibiting density for just the proximal NatA

   No, we did not observe NatA in the proximal site without first classifying on distal NatA. We did look into this by subjecting the particle subset that does not have distal NatA to the same sub-classification as shown in supplementary figure 15 (Mask 2). However, none of the resulting classes showed a signal for proximal NatA. This could either mean that without distal NatA, proximal NatA cannot bind, or it means that without the stabilizing interaction with distal NatA, proximal NatA is simply too flexible to allow a 3D reconstruction.

3. The speculation on the positioning of Naa50 in a hypothetical HsNatE-80S complex takes up a lot of space while not being based on any experimental data. This should be shortened considerably and discussed after the findings on the interaction in trans between proximal and distal NatA.

   We shortened this paragraph and moved it below the NatA-NatA interaction, as requested.

4. The authors argue that the presence of Naa16 in co-IP experiments with Naa15 indicates the existence of a NatA-NatA complex in vivo. However, also here, nuclease treatment of the samples before IP would be required at minimum to rule out a co-purification via polysomes.

   We agree with the reviewers point and have performed supporting Co-IP-WB experiments with prior nuclease treatment. We are pleased to report that we still clearly observe the Naa16-Naa15 interaction, as in our original experiment.

5. Considering that the dimerization of NatA in DLS can be measured only at very high concentrations (mg/ml range), this should be mentioned in the text (line 362).

   Done.

Early Discussion

Beginning at line 367, no new findings are introduced. Results are merely compared and illustrated. The following paragraphs should ideally be restructured and shortened to better fit the nature of a Results section, or be moved to Discussion.

We shortened this paragraph.

Abundancy and "central coordination"

The authors suggest a role for (distal) NatA as a central coordinator of RAFs in vivo. However, in light of other studies (by the groups of Ban and others) and the rather low abundance of NatA compared to NAC in human cells, it is unclear to what degree NatA is actually capable of fulfilling this role. To this end, further results solidifying the involvement in the postulated

complexes in vivo would be helpful.

The binding space around the PTE is very limited, and RAFs (especially enzymes) that need to function while the nascent chain is still short do not do so independently of each other. Instead, their binding at the PTE will be modulated by other RAFs (as seen for e.g. NatA-NAC-MAP1, NatA-MAP2, NatA-Ebp1, NAC-SRP).

By their presence, factors that bind the PTE early during translation will change the properties of the PTE. By offering their binding surface as scaffolding for subsequent RAFs, or by blocking a mutually exclusive binding site, they assume a coordinating role at the ribosome.

The Ban lab and others attribute such a role exclusively to the nascent polypeptide-associated complex (NAC). With its termini, NACα and NACβ can coordinate MAP1, NatA, SRP, and perhaps other RAFs. However, from the current data we are convinced that NAC is not the only RAF to play such a role.
Naa15 is a much larger protein than NAC and its TPR structure serves as a universal binding hub for NAC, Naa10, Naa50, HypK, Ebp1 and proximal NatA. Work on the mammalian ribosome-associated complex (mRAC) (Otto, 2005) also revealed binding of NatA to this co-translationally functioning chaperone. With NatA present in the off-center distal binding site, it will influence all of these factors.

Surprisingly and unfortunately, systematic studies in human cells that quantify NatA and other RAFs for different tissues, under different growth conditions and in the face of disease or cellular stress are missing. Even assuming a much lower abundance of NatA, compared to NAC, Naa15 may still have a coordinative role on ribosomes that have this factor bound. We therefore feel that our description of NatA as a coordinator is in agreement with the current data. However, we have tuned our argumentation.

Figure 4 representation of contact helix (cH) binding surface
In Fig 4c, the authors show a representation of the cH of different factors binding Naa15. Here, the representation of the latter should be reconsidered: Labels for residues 130 and 177 are in the exact same position, despite the surface representation obviously being viewed from different angles. This also makes it more difficult to judge the actual similarity of the binding modes. I suggest re-creating the figure 4c with Naa15 in the same orientation for all ligands.

Fig4c shows the Naa15 α8-10 interaction with different contact helices. For preparing these figures, we used the actual structures of the NatA containing complexes. The viewing angle is the same for all figure elements. However, atom coordinates of Naa15 are not 100% identical between the individual structures. Therefore, the surface representation is also not identical, even though all images were taken from the same viewing angle.
Nevertheless, we did our best do address the reviewer's remark and have made improvements to Fig4c. We added the cartoon representation of Naa15 α8-10 to the figure to function as a common reference point, to better highlight that the viewing angle is identical.

More general, it is one of the key conclusions of the manuscript that the contact helices (cHs) of different RAFs (including NAC, Ebp1 and NatA itself) can interact with the same surface patch of NatA, which is then suggested to be in the position to act as a central coordinator on the ribosome. While the authors themselves do not provide any clear biochemical/genetic evidence that this suggested function exists in vivo, they should acknowledge/discuss previous data regarding the NatA-NAC interaction, showing that rather the UBA domain of NAC makes an essential contribution to NatA binding whereas mutations in the cH have comparably smaller effects.

We agree that for interactions of NAC and NatA, as well as HypK and NatA, the UBA domain is important for the high-affinity interaction. We have previously shown this for HypK and NatA (Weyer et al. 2017) and Lentzsch et al. 2024 observed the same for NAC and NatA. Both papers are cited in the current study. However, it is important to note that while mutations in the UBA domain of NAC had a greater effect than mutations in its cH, the latter still reduced NatA binding 3-4-fold (Lentzsch et al. 2024 Fig. 2g) and almost completely abolished NTA activity (Lentzsch et al. 2024 Fig. 4c).

Even in the absence of NAC, NatA can still bind the PTE with a Kd of ~50 nM (Lentzsch et al Fig. 2g). To aid in NatA recruitment, NAC may simply require the UBA interaction to correctly position itself at the PTE relative to NatA so that its cH can be placed in parallel to Naa15 α8-10. Without the UBA interaction, NAC may no longer be positioned correctly to form the cH contact with Naa15. Mutating the UBA therefore potentially also disrupt the cH interaction. The ~100-fold decrease in NatA binding to the ribosome that Lentzsch et al. 2024 observe when mutating the UBA of NAC may therefore not be attributed only to the UBA domain.

In contrast to NAC, Ebp1 binds much more robustly at the PTE. Ebp1 may not require a UBA domain to stabilize NatA at the distal site. Unfortunately, binding affinities between NatA and the ribosome in the presence of Ebp1 are not available.

In the case of Ebp1 and proximal NatA, the cH is the only contact point to distal NatA. The common feature shared between NAC, HypK, Ebp1 and Naa10 is the cH, and not the UBA domain. Therefore, we favor the idea that while the UBA provides affinity for binding in two cases (NAC and HypK), cH binding adds allosteric regulation in all four cases. For us, this qualifies NatA as a universal RAF binding hub.

Discussion

NAC coordinates co-translational enzymatic activity

Overall, the discussion is overly long and repetitive. It would benefit from inclusion of some of the information (e.g. comparing the effect size of Naa50 and HypK on NatA-NAC binding) in the Results and a removal of much of the redundant statements discussed in other places.

As suggested by the reviewer, we have refined our discussion and condensed potentially repetitive sections to make the manuscript more compact.

1. L427: Lentzsch et al recently showed N-acetylation in dependance on the length of the nascent chain in reticulocyte lysate. This should at least be cited here. → L427 refers to the acetylation of the nascent chain by Naa50 and its possible coordination from the proximal site. Lentzsch et al did not show N-acetylation in dependence of the length of the nascent chain for Naa50.
   However, we have added a citation to this acetylation assay, where we compare the distance of distal Naa10 and proximal Naa10 to the PTE.
2. L463-468: These lines can be omitted or should be moved to the introduction. → Done
3. Fig5a and b) The choice of star-shaped arrangement of RAFs does not add to the readability of the figure and should be replaced with e.g. a table or matrix.→ We would prefer to keep these Venn-like diagrams as they are, as we would like to stay consistent with a previous paper (Klein et al. 2024). However, we made an effort to improve the figure caption, to improve readability.
4. L535: Why not reconstituting this complex (NatA-NatA-MAP1-80S)? The factors should all be available. → We agree that this could be interesting. However, even with the factors available, much time would have to be spent on the grid-preparation and optimization procedure, on screening, preliminary data acquisition and processing, large scale data acquisition and extensive processing, model building and refinement, analysis, interpretation and discussion of results, as well as the data deposition and incorporation into our manuscript. Even assuming that everything works perfectly on the first try, this would be very time demanding.
5. L541: This is redundant information that has been discussed multiple times so far in the paper. → We agree and removed the redundant sentence from the manuscript.
6. L553: Here, the same information is restated in different ways at least three times. → To avoid unnecessary repetition, we have condensed the referenced section and removed repetitive statements.
7. L572: The discussion of the regulation involving HypK appears to be too long, especially since it is based on very limited data. → We slightly disagree here and want to provide a thorough analysis of the structure we describe. We tried to shorten this section, but we maintain that all statements add value to the discussion. We are worried that further shortening of these paragraphs would leave some questions unaddressed.

Minor comments:

Figure 1a: The choice of a striped bar for the non-existent binding of NatA-HypK to NAC is misleading and should be corrected. If no binding could be measured, the measurement should be shown as zero.
Figure 1b/d: A different color for the currently gray curves would improve contrast and readability.
Figure 1 Legend: “demolish” -> “abolish”
Figure 2 Legend: “fixate”
Figure 2 Legend: For (b) and (d) it should be clarified that these are partial models based on the experimental data, which omit NatA (b) and Ebp1 (d).
Figure 2d: For consistency, the arrow should point towards the “new” (teal) conformation observed in the Ebp1-NatA complex.
L 297: “touches down”?
L269: Omit “down” just write “towards the PTE”
L285: Add citation to previous work
Fig 3c: Here, too, the authors show a model of proximal NatA binding to the ribosome which is derived from their data on the ternary NatA-NatA-80S complex by omission of the distal NatA. This should be noted in the legend.
Supp Fig 15: Labels “remove classes without….” Should both be one level higher.
Fig 3c: The cH of Naa10 should be colored red as in (a) and (b) for consistency.

We thank the reviewer for the attention to detail and agree with the recommended minor changes, all of which we have implemented in our Manuscript.

Reviewer #2 (Remarks to the Author):

Recommendation: publish with minor revision

In this manuscript, Klein et al. demonstrate that NatA serves as a central coordinator of co-translational protein maturation by dynamically assembling with multiple ribosome-associated factors (RAFs) at the polypeptide tunnel exit. Biochemical assays—including SEC-MALS and biolayer interferometry (BLI)—reveal that NAC can form ribosome-independent complexes with MAP1, NatA, and NatE. In particular, BLI "order-of-addition" experiments confirm that NAC mediates the interaction between NatA and MAP1. The authors further show that while Naa50 modestly impairs NatA's binding to NAC, HypK completely blocks this interaction. This is in particular surprising because a recent NatA-HypK-NAC-80S cryo-EM structure showed that NAC can bind to NatA in presence of HypK at the ribosome, but a possible structural explanation is discussed. Cryo-electron microscopy analysis uncovers that NatA can form a ternary complex with Ebp1 on the ribosome. In this assembly, Ebp1 binding remodels rRNA elements and competes with factors such as NACα and HypK for a conserved hydrophobic site on Naa15. Mass spectrometry experiments confirm that endogenous Naa15 interacts with Naa10, NACα, HypK, and Ebp1. Moreover, reanalysis of a NatA–80S dataset reveals a second, proximal binding site at the ribosome that positions the catalytic subunit Naa10 closer to emerging nascent chains, suggesting a mechanism for rapid substrate acetylation. Notably, proximal Naa10 contacts distal Naa15 at a conserved surface—centered on a critical tryptophan residue—similar to the binding sites used by NAC, MAP1, and Ebp1, indicating competitive interactions at the tunnel exit. Together, these findings position NatA as a versatile interaction hub that orchestrates multiple co-translational processing events critical for proteostasis.

This manuscript is well written and provides significant mechanistic insights into the co-translational regulation of protein maturation—a fundamental process in eukaryotic biology with broad implications for proteostasis and disease. By integrating high-resolution structural data with rigorous biochemical and biophysical analyses, the study not only expands our understanding of the multifaceted roles of NatA but also unveils novel mechanisms at the ribosomal tunnel exit. The discovery of a second, proximal binding site for NatA that enhances substrate accessibility is particularly innovative and could reshape how we view the spatial organization of co-translational modifications. Given its broad relevance to cell biology, structural biology, and biochemistry, this work is suitable for publication in Nature Communications.

We thank the reviewer for the detailed assessment, and positive reception of our work. We agree with this comprehensive summary of our manuscript and are pleased that the reviewer finds it suitable for publication in Nature Communications.

Weakness of the manuscript:
The major weakness is that the study relies on in vitro biochemical assays and structural data and cellular experiments would strengthen the physiological relevance of the observed assemblies. While the discovery of distal and proximal binding sites for NatA is intriguing, the manuscript does not fully clarify how these sites differentially contribute to co-translational acetylation and overall proteostasis in cells.
The competitive binding observed among factors like NAC, HypK, and Ebp1 is well demonstrated in vitro. However, the mechanisms that modulate this competition in a cellular context remain somewhat speculative.
Furthermore, some flexible regions or extensions in the cryo-EM reconstructions are less well resolved, which may leave uncertainties regarding the complete interaction interfaces.
Since these questions open up further exciting directions of investigations and cannot be addressed in short periods of time, these points should not detract from the overall high quality of the work. Many groups working on the topic would as well benefit from these data, therefore the manuscript should be published as it is as an in vitro structural work.

While cellular experiments that put our structures in an *in vivo* context would be valuable in order to attain a deeper understanding of NatA regulation, we agree with the reviewer that such investigations would be very time demanding and beyond the scope of this manuscript.

Suggestions:
N-terminal acetylation is predominantly a co-translational modification. Might the assembly of NatA complexes in vitro in the absence of ribosomes reflect potential post-translational Nt-processing events like Nt-acetylation? Would immunoprecipitiations from Naa15 or Naa10 from ribosomal and cytoplasmic fractionations show the same RAF interactions partners?

We designed an experimental setup independent of the ribosome to learn more about the molecular details of these RAF interactions. At the ribosome, NAC may remain ribosome-associated via its NACβ anchoring helix and use its long C-terminal extension of NACβ to interact with MAP1 to the PTE. In such a scenario, it may be advantageous that NAC can form strong protein-protein interactions with MAP1 to allow stabilization at the PTE. The substrates of these assemblies are the N-termini of nascent chains, and to date there is no evidence for post-translational modification.
We believe that under "normal" cellular conditions, the majority of NatA is in complex with HypK (although this has not been clearly shown). This would prevent an interaction with NAC in the absence of the ribosome (as shown by our BLI

experiments).We show that NAC and the ribosome are both required to allow for NatA activation. By this mechanism, NatA activity should mostly be confined to the ribosome and prevent a post-translational NTA activity of NatA.

Immunoprecipitation of Naa15 or Naa10 from the cytoplasmic fractions will likely not reveal an interaction with NAC or Ebp1, due to HypK. In the case of Ebp1, we would also predict that the mostly hydrophobic interaction between the Ebp1-cH and Naa15 would be too weak to allow complex formation between NatA and Ebp1, in the absence of the ribosome.

Minor Point
Fig.1: It would be beneficial to add the single measured points or error bars where appropriate.

BLI affinity experiments were done in duplicates. We have added the two individual measurement points to figure 1a.

Reviewer #3 (Remarks to the Author):

In this manuscript, the authors investigate NAC interactions with NATs and MAPs in vitro, demonstrating that NatA can form high-affinity complexes with other ribosome-associated factors in the absence of ribosomes. Additionally, they resolve the structures of EBP1 and NatA in complex with the ribosome and revisit a previous dataset to identify a NatA dimer interacting at distinct positions near the tunnel exit.
While the study identifies interesting interactions, ribosome docking positions, and tertiary and quaternary complexes, the biological relevance of these findings remains unclear. The proposed functional roles of these complexes are largely speculative, and it is uncertain whether they exist in vivo under physiological conditions. Moreover, while the structural data provide valuable insights, they do not seem sufficient to support the proposed coordination mechanism. Strengthening the connection between these structures and a well-defined biological function would significantly improve the manuscript.

We thank the reviewer for careful assessment of our work, and helpful suggestions for improving our manuscript. We want to stress that co-translational protein maturation at the human ribosome is an immensely complex process and will require many interdisciplinary studies to unveil its *in vivo* workings. In our opinion, attempts to implement targeted *in vivo* experiments to unveil the physiological significance of our structures are beyond the scope of this manuscript.

We focused our efforts on the question of "What can happen at the tunnel exit?" Our co-immunoprecipitation/MS experiments (now refined, see below) clearly underline the in vivo relevance of the described interactions. Our discoveries of novel NatA interactions, including a second binding site and the interplay with Ebp1, provide a new angle on how we view co-translational N-terminal acetylation.

Major comments:
1) While NAC can form a ribosome-independent complex with MAP1 and NatA in vitro, the biological relevance of this interaction remains unclear. It has been proposed that NAC facilitates the release of HypK-mediated inhibition of NatA on the ribosome, thereby localizing NatA activity at the ribosomal tunnel exit. However, the formation of a ribosome-independent complex appears to contradict this model; a ribosome-independent NAC-NatA complex may activate NatA, disrupting such NAC-mediated localization system and being detrimental for the cell.
In agreement with the potential danger of NatA-NAC ribosome-independent complexes, the authors report that HypK prevents the NAC-NatA interaction off the ribosome. This suggests that HypK may actively prevent off-the-ribosome NatA interactions with NAC in the cell. Could this mechanism explain the observed data? Addressing this question would help clarify the functional implications of the reported interactions.

We absolutely agree with the reviewer that under normal circumstances NatA activity should be confined to the ribosome. We still found it useful to perform BLI and SEC-MALS experiments in the absence of the ribosome for numerous reasons:

- We observed contacts between NAC and MAP1, and between NatA and NAC in our cryo-EM map. We wanted to test whether these contacts are strong enough to allow complex formation without the ribosome, and quantify the interaction strength. This allowed us to compare the strength of binding between NAC and MAP1, to the strength of binding between NAC and NatA.
- In addition, our experimental setup allowed us to test how other RAFs (HypK, Naa50) influence the protein-protein interaction between NatA and NAC.
- Building on previous NatA-NAC (Klein et al. 2024, Lentzsch et al, 2024) and NatA/HypK data (Weyer et al, 2017, and Gottlieb and Marmorstein 2018) we were able to attain a deeper understanding of how NatA activation is accomplished on a molecular basis. In our BLI experiments we observed that NAC is unable to bind to NatA-HypK. In our cryo-EM studies, we meanwhile showed that NatA-HypK is unable to bind to ribosomes without NAC. However, when NAC is bound at the tunnel exit, NatA-HypK can bind the ribosome and form a quaternary assembly (NatA-HypK-NAC-80S), as shown by Lentzsch et al. (2024). The restructuring of HypK and activation of NatA therefore requires both the ribosomal surface and NAC at the same time. BLI experiments in the absence of the ribosome were therefore critical in obtaining a mechanistic understanding of the allosteric modulation of NatA.

2) The authors use mass spectrometry (Co-IP-MS) to assess the biological relevance of the observed interactions. However, cellular lysates were not treated with RNases prior to immunoprecipitation, meaning that polysomes remained intact. As a result, immunoprecipitating a ribosome-associated factor is likely to recover entire polysomes, leading to the co-purification of ribosome-associated factors that may not coexist on the same ribosome but are instead present within the same polysome. To support their claims, the authors must demonstrate that their immunoprecipitation is specific to individual ribosomes rather than polysomes. This limitation significantly weakens the conclusions drawn from these experiments and, unfortunately, leaves the existence of these complexes in vivo unproven.

We have repeated the co-immunoprecipitation experiments using cell lysates pre-treated with RNase T1 and RNase I. These experiments confirmed that both NAA15 and NAA10 interact with EBP1 following RNase digestion, indicating that the interaction is not polysome-dependent (Suppl. Figure 13).

3) Along these lines, can the authors comment on why EBP1 is only significantly enriched in the immunoprecipitate of NAA15 but not of NAA10 (Supp. Fig. 13)? If EBP1 and NatA were in complex on the same ribosome, one would expect that immunoprecipitating NAA10 would also lead to EBP1 enrichment. Overall, EBP1 enrichment appears relatively low compared to other ribosome-associated factors. Together with the methodological limitations discussed earlier, these observations further weaken the Co-IP-MS data as support for the existence of the proposed complex in cells.

The absence of EBP1 enrichment in the co-IP MS experiment may be due to interference from the anti-NAA10 antibody disrupting the NAA10–EBP1 interaction. We therefore performed new co-IP experiments with RNase digested cell lysates and tagged proteins and used anti-tag antibodies for co-immunoprecipitation. The results showed that both NAA10 and NAA15 interact with EBP1 (and NAA16).

4) His-tags are known to enhance the affinity of proteins for ribosomes, as previously reported (Supplemental Fig. S6 in Pech et al., J Biol Chem, 2010). Can the authors exclude the possibility that this effect is influencing their conclusions regarding EBP1 affinity and the salt resistance of its ribosome interaction?

We agree that the addition of positively charged residues to a protein will increase its affinity for the negatively charged ribosomal surface (formed by the rRNA). However, in our Western blotting experiment, we were merely interested to deduce how much salt would be required to displace Ebp1, to obtain empty ribosomes, knowing that we could later confirm our findings by cryo-EM.

Indeed, our observation that 600 mM salt is required to displace Ebp1 was further validated by several cryo-EM structures. Previous cryo-EM data collected on human ribosomes that were treated only with 500 mM salt still retain endogenous co-purified Ebp1 (and NAC) (Klein et al. 2024). In contrast, 600 mM salt in the buffer fully displaced Ebp1 from the ribosome, as confirmed e.g. by the NatA-NatA-80S dataset, which no longer showed endogenous Ebp1 at the PTE (this study).

5) I am not convinced of the relevance of the described proximal site of NatA or whether this binding site—or a dimeric state—is significantly populated in vivo. First, the authors identified this site through reanalysis of their dataset, suggesting that it is occupied by only a minor fraction of ribosomes in their cryo-EM sample. Additionally, NatA is present at substoichiometric levels compared to EBP1 and NAC, and this proximal binding site would be mutually exclusive with these highly abundant factors. If the proximal site is already sparsely occupied in an in vitro reconstituted system, it seems unlikely that it could effectively compete with other ubiquitous ribosome-associated factors in vivo. Furthermore, the functionality of a NatA dimer remains unclear. While the authors propose multiple potential mechanisms, these remain speculative.
It is therefore possible that the observed dimeric state is an artifact of the in vitro reconstitution conditions used for cryo-EM. This concern is further compounded by the limitations in the Co-IP-MS data discussed earlier. Addressing these issues, either through additional biochemical validation or functional assays, would be necessary to support the proposed mechanisms. So far, the authors' data demonstrate that these factors are able to simultaneously bind a ribosome, this does not imply that they represent a functional state or a coordination mechanism.

It is true that NatA occupies only a small fraction of the analyzed ribosomes in our dataset. While it may also be true that NatA is a much less abundant protein than Ebp1 and NAC, data on this are lacking in the human system. Systematic studies that thoroughly quantify the abundance of RAFs in different human tissues, under varying growth conditions and when facing disease, cellular stress and aging are missing. Also, studies are missing which elucidate how these RAFs may be dispersed throughout the cell. It is unlikely that RAFs would be evenly distributed.

Work from the Förster lab, for instance, found that ribosomes on the ER membrane that interact with the OSTA translocon or the multipass translocon cluster together in separate patches (Gemmer et al., 2023). As referenced in our manuscript, a recent study also found that ribosomes that have NAC bound typically do not have Ebp1, and vice versa, but Ebp1 and NAC bind to about the same fraction of ribosomes (Zheng, W. et al., 2024). Such observations corroborate the idea that RAFs are directed to distinct ribosome populations, rather than being randomly dispersed throughout the cell. While we agree that NAC and Ebp1 are higher in abundance compared to other RAFs , and that their interaction with NatA in the context of translation may represent more canonical mechanisms, we do not want to exclude the possibility of alternative mechanisms that might be relevant under specific conditions for distinct ribosomal populations. One of these may be the dimerization of NatA at the PTE, possibly even between different isoforms of NatA (Naa15, Naa16, Naa10, Naa11, Naa12).

When we first obtained NatA-NatA-80S structure, we critically assessed whether this is may be an *in vitro* artifact. However, we became convinced that it could represent a functionally relevant state for two main reasons:

- Naa10 has a contact helix of previously unknown function that structurally rearranges in the proximal site to contact distal Naa15 at its conserved cH binding site.

- Yeast NatB also dimerizes at the ribosome (with a completely different interaction interface than NatA), indicating that the presence of two NATs is a conserved mechanism employed for different NATs in different organisms.

We find it unlikely that the observed specific interactions and reoccurring features are merely a coincidence or artifact.

6) The authors claim that NatA plays a coordinating role in multifactor complexes at the ribosomal tunnel exit, as reflected in the title of the manuscript. However, the presented data do not support this conclusion. The authors observe that EBP1 shares the same binding site that NAC uses to displace HypK-cH for NatA activation. This raises the possibility that EBP1, like NAC, could facilitate NatA activation at the ribosome. Given the ubiquitous presence of both NAC and EBP1 on ribosomes, these findings suggest that NatA activation does not depend on a specific ribosome-associated factor but may instead occur through a factor-independent mechanism. This interpretation seems plausible but does not contradict the notion that NatA acts as a coordinator of other factors.

We do not believe that NatA actively recruits either NAC or Ebp1. We agree that our data suggest that many factors, including NAC, Ebp1 and proximal NatA might allow the displacement of HypK and activation of distal NatA via their contact helices (cHs).
The proposed coordinating role of NatA stems from its ability to form multi-protein assemblies with so many other RAFs, including Naa10, Naa50, HypK, NAC, Ebp1 and possibly others (see also our response to reviewer #1, regarding the role of NatA as a central coordinator). At the PTE, the TPR structure of Naa15 acts as a universal adapter to bind and coordinate other RAFs.

7) The authors' interpretation hinges on the assumption that a pool of HypK-free NatA exists in the cell. While they acknowledge that this is not established, their own mass spectrometry data show that HypK co-purifies with NAA15 and NAA10 at similar levels. Although Co-IP-MS is not strictly quantitative, this observation suggests that most cellular NatA exists in complex with HypK. This would agree with the need of keeping NatA inactive until it associates with the ribosome. Can authors comment on this?

We agree that most NatA is likely regulated by HypK, and that it makes sense to keep NatA inactive until it reaches the ribosome.
However, as mentioned before, thorough studies that quantify NatA and its ratio to HypK are lacking. Naa15 and its isoform Naa16, as well as Naa10 and its isoforms Naa11 and Naa12 may form diverse assemblies with Naa50 and/or HypK. If, when, and how these different modules come together at the ribosome remains to be shown. Until such studies are conducted, we want to remain open to the possibility that a pool of NatA may exist without HypK under certain conditions.

In summary, while the structural data presented in this study are valuable for understanding ribosome-associated multicomplex interactions, I find that they are overinterpreted to propose mechanisms that remain hypothetical. Due to technical limitations, particularly in the Co-IP-MS experiments, the biological relevance of the described interactions is not convincingly demonstrated.

We are happy that the reviewer finds the structural data presented in our study valuable. We have addressed the technical limitations regarding the Co-IP-MS experiments and hope that this alleviates his remaining concerns.

Page 11: "HypK-contact helices (cH) do".
Page 26: "…MAP2-like pseudoenzyme".

We thank the reviewer for catching these typos, which have now been removed from the manuscript.

REVIEWER COMMENTS

Reviewer #1 (Remarks to the Author):

Reviewer comments revised version
NCOMMs-24-85546

In the revised version of their manuscript Klein et al. addressed several of our concerns satisfactorily. Nevertheless, some of the old and new data are still inconclusive and partially not appropriately interpreted and represented in the main text.

In vivo IP:
1. It should be clearly stated in the figure legend (Suppl. Fig 13) and the main text, that the co-immunoprecipitation Mass Spectrometry experiments are done without RNase treatment, whereas the newly added subsequent Co-IP Western blotting experiments were done with (or control without) RNase treatment.

We followed the reviewer's advice and have added this information to the figure legend and to the main text as requested.

2. Line 252 states in the revised version:

"To rule out that the Co-IP was polysome dependent (NAA15 and Ebp1 bound to separate ribosomes), lysates were treated with RNaseI or T1. However, subsequent Co-IP Western blotting experiments still clearly show the NAA15-EBP1 interaction."

However, the data presented in the new Suppl Fig. 13 b) and c) are rather inconclusive and this statement does not acknowledge the fact that after treatment with both RNases much less NAA15 co-immunoprecipitated with EBP1 than in the non-treated control indicating that both proteins were at least with very substantial amounts located on different ribosomes. In the case of RNaseI treatment surprisingly much less NAA15, but much more Naa10 precipitates with EBP1 than in the untreated control. This imbalance may indicate that Naa10 indeed associates but perhaps together with NAA16 and not NAA15. Moreover, in the case of RNase T1 treatment hardly any NAA15 or NAA10 co-immunoprecipitated with EBP1 even though the lysates nicely show similar levels of expression for all three tagged components. This should be clearly described in the main text (not only in the supplement) and critically discussed.
In the V5 –IP /Western blot experiment presented in the new Suppl. Fig 13 c the authors address the question whether an interaction between two distinct

endogenous NatA complexes (namely, NAA15/NAA10 and NAA16/NAA10) might occur in vivo inside HeLa cells.
Here again, NAA15-V5 with or without RNase treatment co-immunoprecipitated only a minor fraction of Naa16, whereas NAA10-V5 co-immunoprecipitated a 2- to 3-fold excess of NAA16 (over NAA10) even though the lysates nicely show similar levels of expression for all three components.
Taken together Suppl Fig 13 b) and c) could also imply that EBP1 rather interacts with a NAA16/NAA10, than with a NAA15/NAA10 complex in vivo.
These data require a better description in the main text than the highly simplified statements in the revised main text:

Line 254
However, subsequent Co-IP Western blotting experiments still clearly show the NAA15-EBP1 interaction."
and
Line 351
"However, subsequent Co-IP Western blotting experiments still clearly show NAA15-NAA16 interaction."

We thank the reviewer for the detailed assessment of our Co-IP experiments. To accommodate these suggestions, we have made changes to the main text and supplementary materials.

Proximal versus distal NatA function
Our former request was (5.):
"Considering that the dimerization of NatA in DLS can be measured only at very high concentrations (mg/ml range), this should be mentioned in the text."
Previous main text:
"In order to test whether NAA15 composed NatA can dimerize in vitro and independent of the ribosome, we measured dynamic light scattering (DLS) of increasing NatA concentrations. Indeed, we observe that dimerization also occurs in the absence of the ribosome (Supplementary Fig. 20)."
Revised main text:
"In order to test whether NAA15 composed NatA can dimerize in vitro and independent of the ribosome, we measured dynamic light scattering (DLS) of increasing NatA concentrations (0.03-8 mg/ml). Indeed, we observe that dimerization also occurs in the absence of the ribosome (Supplementary Fig. 20)."
This is a misleading statement, since up to a concentration as high as 0.5mg/ml an average hydrodynamic radius of 5-6 nm representative of a single NatA heterodimer is observed. These results should also be described in the main text in a more complete and accurate way.

We have added to this sentence to make it clearer that we only observe a dimerization of NatA at higher concentrations (above ~0.5 mg/ml).

Taken together, the authors made a considerable effort to address the main points, however, without getting clear and consistent results. Therefore, we are not entirely convinced that the findings observed in vitro are indeed of physiological significance in the cell. We thus feel that the authors must tone down the text with respect to the in vivo data. After being completer and more transparent about the in vivo data in the main text (as suggested above), the authors should clearly state latest in the discussion that it is not yet clear to what end their interesting in vitro findings represent physiological behaviour. Nevertheless, even without a totally convincing in vivo support for their data, I find their findings interesting and solid enough as to warrant publication in Nature Comms as soon as the data are represented in a more cautious way.

We thank the reviewer for their assessment of our revised manuscript.
As requested, we have further toned-down points relating to the *in vivo* data to make it clear that further experiments are required to assign a physiological function to our complexes and validate their *in vivo* relevance.

With our more complete and transparent description of the *in vivo* data, we hope that reviewer #1 now supports publication of our data.

Reviewer #2 (Remarks to the Author):

I have only a few minor remaining points:

In Figure 1, replicates were added for panel a, but not for panel c. Replicates, SD or at least clear indication of n should be included consistently.

The spiking experiment was done once (n=1), as it only serves to validate the observations already made in Figure 1a and Figure 1d. We have added this to the figure description for clarity.

In Figure 2d, the “Naa15 rotated” label is barely legible

We have improved the readability of this label.

In the main text (line 252), please reference Supplementary Fig. 13a and at line 255, Supplementary Fig. 13b and c.

Done!

There is no textual reference to Supplementary Fig. 12a and b, please check.

There is a reference to Supplementary Fig 12a and b in line 304 (underneath figure 2).

Overall, the manuscript is much improved and suitable for publication after addressing these small points.

We thank the reviewer for the positive feedback on our revised manuscript.

Reviewer #3 (Remarks to the Author):

I thank the authors for their response, which addressed some of my initial concerns. I appreciate the effort to clarify points raised in the first round, and I continue to value the structural insights presented in this study. That said, a few key issues remain unresolved.

Critical points

1. Title of the manuscript and claim of NatA coordinating tunnel exit complexes
I remain unconvinced by the title and the claim that NatA coordinates multi-factor complexes at the ribosomal tunnel exit. Authors show that NatA can form multiple complexes with different factors at the ribosomal tunnel exit. Yet, evidence showing that NatA regulates or coordinates the formation and activity of these complexes is not provided.
On the contrary, the data suggest that NAC, and possibly EBP1, regulate NatA activity at the ribosome. For this reason, the title should be revised to more accurately reflect the findings.

We have changed to title from “NatA coordinates…” to “NatA engages in…” to address the reviewers suggestion.

2. Co-IP-MS experiments
First, from a technical perspective, my concern regarding the Co-IP-MS data remains (point 2 of my previous review). Although the authors attempted to validate the Co-IPs under RNase-treated conditions using WB, the Co-IP-MS itself must also be repeated with RNase-pretreated lysates to ensure the data reflect direct ribosomal interactions. Without this, the results represent proteins co-existing in polysomes rather than on individual ribosomes, which is misleading. This experiment must either be repeated using RNase-treated polysomes or removed from the manuscript.

Second, the new western blot evidence presented to support a direct NatA-EBP1 interaction on individual ribosomes (generated by RNase treatment) is not convincing. Instead, it shows a marked reduction in the co-immunoprecipitation of Naa10 and Naa15 upon RNase treatment, especially with RNase T1. The levels of co-immunoprecipitated Naa10 after RNase T1 treatment approach those of the control (supplementary Fig 13, lane 3) rather than the untreated condition. This raises concerns that the NatA - EBP1 interaction strongly depends on polysome integrity, making its in vivo co-existence on the same ribosome uncertain. While I acknowledge that RNase digestion itself can affect ribosome binding and that the NatA-EBP1 complex might be of low abundance, the authors should address this issue directly and temper their claims regarding in vivo relevance.

In summary, I strongly recommend that all Co-IP experiments performed without prior RNase treatment either be repeated under proper conditions or removed. Claims derived from these experiments must be aligned with the actual data.

We thank the reviewer for these comments and have revised the manuscript to avoid any misleading interpretation. Based on the feedback from the other reviewers and the Editor, we decided to keep the original IP-MS data without RNase treatment, but now clearly stating which experiments are done with and without RNase treatment both in the main text and the Suppl. Figure 13 legend.

Moreover, we have revised the description of Supplementary Fig. 13b and c in the main text to provide a more detailed and balanced interpretation of the RNase-treated Co-IP Western blot experiments and their caveats.

Other comments

3. Proximal NatA binding site
It remains unclear whether the proximal NatA binding site exists in vivo and what its proposed function might be. So far, this conformation has only been observed in vitro under restricted conditions and in the absence of HypK. Since the authors show that NatA-HypK cannot self-promote ribosome binding, this pathway seems unlikely to relief HypK inhibition. The suggestion that free NatA may serve this role is possible but remains speculative. Providing evidence that such a mechanism is plausible would strengthen this interpretation. At present, any functional assignment to the NatA dimer is speculative and the claims should be toned down accordingly.

We remain convinced of the significance of the NatA dimer for three reasons: **(1)** The specific conformational change in the proximal Naa10-cH that contacts the conserved hydrophobic patch on distal Naa15. **(2)** The fact that a dimerization of

NatB (different NAT and different organism) has also been observed, suggesting NAT dimerization to be a well-conserved mechanism to facilitate N-terminal acetylation at the ribosome. **(3)** We can detect a NatA-NatA dimer in our Co-IP experiments even after RNAse treatment.

However, we agree that further *in vivo* data will be required to fully understand the role of the NatA dimer. In addition, quantitative data on NatA vs. NatA-HypK in different cell types, growth conditions, and perhaps under stress would be helpful to shed light on the relevance of a NatA dimer.

Nonetheless, we have made an effort to tone down any statements that might suggest a physiological role of a NatA dimer.

Overall assessment
Despite these reservations, I want to emphasize that the structural data are of high value to the community and making them available will be useful for the field. However, the lack of a clearly defined biological context or demonstrated function reduces the overall impact of the study.
I would support publication in Nature Communications provided that (i) the Co-IP-MS experiments are repeated with RNase-treated lysates (or else removed) and (ii) the manuscript title is adjusted to better reflect the actual findings.

We thank the reviewer for assessing our revised manuscript. We have changed the manuscript title to comply with the reviewer's suggestion. However, we still find that our Co-IP-MS data has value, especially in combination with our additional Co-IP-WB data. In our opinion, removing the Co-IP-MS data would not improve the manuscript.

However, to also address the suggestions of reviewer #1, we have made changes to the main text to be clearer and more transparent about the results of these IP experiments. We hope that this will alleviate any remaining concerns.

Reviewer #4 (Remarks to the Author):

The authors have addressed the majority of concerns raised by the previous Reviewers. However, considering that the manuscript's novel findings center on the Ebp1-NatA-80S and NatA-NatA-80S complexes, the most critical issues (three reviews all had the same concerns) regarding the biological relevance of these two complexes remain unresolved. In the revision the authors provide additional evidence (via co-immunoprecipitation after RNase I/T treatment) for the existence of Ebp1-NatA and Naa15-Naa16 interactions in vivo, the claim that Ebp1-NatA-80S and NatA-NatA-80S complexes function in vivo lacks convincing experimental support.

Moreover, both two complexes are not organized in a manner that could achieve successive NME and NTA.
For Ebp1-NatA-80S: This complex lacks N-terminal methionine excision (NME) activity, raising questions about its functional relevance in sequential NME/NTA, though the authors proposed a place-holder function of Ebp1.
For NatA-NatA-80S: The positioning of the proximal NatA within this complex would sterically hinder the binding of both NAC-MAP1 and MAP2, preventing coordinated NME and NTA. The authors propose a potential "NatA-NatA-MAP1-80S" complex to overcome this, but crucially, they did not pursue structural validation as previously recommended by Reviewer #1. Furthermore, while functional assays probing acetylation activity were suggested, mechanistic details confirming how NatA-NatA achieves NTA are still lacking.
In summary, while progress has been made in addressing some previous concerns, this revised manuscript still lacks sufficient experimental evidence to substantiate the functionality of the two in vitro complexes.

We thank the reviewer for taking the time to assess our revised manuscript. We agree that there are open questions that remain to be addressed to allow a full understanding of N-terminal acetylation by NatA. Nonetheless, we strongly agree with the statements of reviewer #5 and are convinced that our solid structural description of highly specific NatA-NatA and NatA-Ebp1 interactions at the human ribosome will direct the field for follow-up investigations. With our manuscript we tried to answer the question of “What molecular interactions can take place at the PTE in the presence of NatA”. In our opinion, the questions of when, where and why these interactions take place would require many new experiments and are beyond the scope of this manuscript.

Reviewer #5 (Remarks to the Author):

In this articles Klein and colleagues analyze the interplay of NatA with other ribosome-associated factors (RAFs) at the ribosome and apart from it.
N-terminal acetylation (NTA) is a major protein modification in eukaryotes, mainly carried out by the NatA complex after N-terminal methionine excision (NME).
This study reveals that NatA not only acts at the ribosome but can also form ribosome-independent assemblies with several ribosome-associated factors (RAFs). At the ribosome, NatA can form ternary complexes with either the pseudoenzyme Ebp1 or another NatA copy, enabling flexible substrate coordination. The findings suggest that NatA serves as an interaction hub, organizing multiple factors to coordinate co-translational protein maturation.
The authors mainly use an in vitro approach, but during the revision added CoIP experiments to corroborate their findings also in cellulo.
I have specifically been asked to assess the comments of reviewer 1, who apparently was not available anymore for the reviewing process.

In my opinion, the following comments appear to be satisfactorily addressed in full – I have no further comments
• Fig 1 effect size
• Proximal vs Distal NatA, Function Issues 2, 3, 4 and 5
• Early Discussion – this should have never been a major issue
• Figure 4 representation of contact helix (cH) binding surface
• Discussion - NAC coordinates co-translational enzymatic activity
• Minor comments
In my opinion, the following comments appear to be partially addressed – see my further comments
• Proximal vs Distal NatA, Function Issue 1 – I agree with the authors and I am also not convinced that "an acetylation experiment would allow (us) to distinguish the activity of distal and proximal NatA". I am fine with the response of the authors and I am convinced that the authors would also like to figure out the functional role of the distal NatA molecule.
• Abundancy and "central coordination" – I fully agree with the reviewer that this is a critical point, but it is also wishful thinking to answer these complex questions in vivo. I do agree with the response of the authors that the presented study provides suitable models that need to be tested in the future. The low abundance indeed makes it hard to associate such a central role, but this rather also asks for a quantification of RAFs in cells under different conditions. The study does show that eventually NatA does play a more central role than previously acknowledged and that NAC is not the only coordinating center. In my opinion, authors did work on the respective sections and down toned some of their statements.

Overall assessment – the authors provided several new insights into these complex regulatory layer at the ribosome that is central to a large fraction of our proteomes. Furthermore, the authors provide clear biochemical and structural evidence that supports their main claims. I do agree with reviewer 1 (and reviewer 3) that some parts of the study will still require additional validation in vivo. In my opinion, the authors addressed these issues by performing CoIP experiments at a satisfactorily level – however, I am aware that many more in vivo experiments are needed to clarify these RAF networks (at the ribosome and apart form it). This does leave a taste of uncertainty, but this is most often the case for studies that are mainly focusing on revealing the mechanistic reasons behind cellular machines.

For all of the above reasons, I would support publication of the work in Nature Communications. Not because, this study provides all the final answers, but because it points the field into directions of several important links between RAFs that need to be investigated in the future (under different cellular conditions). I think that most of the overstatements have been removed during the revision process – I did not find outrageous statements any more.

We thank the reviewer for taking the time to thoroughly assess our work and the positive reception of our revised manuscript. We are pleased that reviewer #5 supports publication of our work in Nature Communications.
